# Visuomotor Transformations Underlying Hunting Behavior in Zebrafish

**DOI:** 10.1016/j.cub.2015.01.042

**Published:** 2015-03-30

**Authors:** Isaac H. Bianco, Florian Engert

**Affiliations:** 1 Department of Molecular and Cellular Biology and Center for Brain Science, Harvard University, Cambridge, MA 02138, USA

## Abstract

Visuomotor circuits filter visual information and determine whether or not to engage downstream motor modules to produce behavioral outputs. However, the circuit mechanisms that mediate and link perception of salient stimuli to execution of an adaptive response are poorly understood. We combined a virtual hunting assay for tethered larval zebrafish with two-photon functional calcium imaging to simultaneously monitor neuronal activity in the optic tectum during naturalistic behavior. Hunting responses showed mixed selectivity for combinations of visual features, specifically stimulus size, speed, and contrast polarity. We identified a subset of tectal neurons with similar highly selective tuning, which show non-linear mixed selectivity for visual features and are likely to mediate the perceptual recognition of prey. By comparing neural dynamics in the optic tectum during response versus non-response trials, we discovered premotor population activity that specifically preceded initiation of hunting behavior and exhibited anatomical localization that correlated with motor variables. In summary, the optic tectum contains non-linear mixed selectivity neurons that are likely to mediate reliable detection of ethologically relevant sensory stimuli. Recruitment of small tectal assemblies appears to link perception to action by providing the premotor commands that release hunting responses. These findings allow us to propose a model circuit for the visuomotor transformations underlying a natural behavior.

## Highlights

• Zebrafish hunting responses are triggered by conjunctions of visual features
• Tectal neurons show non-linear mixed selectivity for prey-like visual stimuli
• Tectal assemblies show premotor activity specifically preceding hunting responses

## Teaser

By combining a virtual hunting assay with functional calcium imaging, Bianco and Engert identify highly selective neurons in the zebrafish optic tectum that are likely to underlie visual prey recognition. The recruitment of tectal assemblies appears to link perception to action by providing the premotor commands that release hunting responses.

## Introduction

To generate visually guided behavior, the nervous system extracts task-relevant information from the retinal image to select and control an appropriate response. Over 50 years ago, neuroethologists introduced the idea that specific behaviors can be triggered by “key stimuli,” delivered under appropriate conditions [1, 2]. In this context, individual neurons have been discovered in visual pathways that are proposed to function as “feature detectors.” Such neurons are selective for specific spatiotemporal patterns within the visual scene and include neurons tuned to visual features that define key stimuli. Notably, stimulus-response pathways are subject to various modulating influences, and consequently “key stimuli” do not always trigger a response. Motivational state, arousal, attention, recent experience, and long-term memory can influence response probability, stimulus preference, and the choice of motor outputs (e.g., [3, 4]). Therefore, to understand how sensorimotor circuits link perception to action, it is necessary to monitor neural activity and behavior simultaneously. In larval zebrafish, the small size and optical transparency of the nervous system allows functional imaging of neural activity at cellular resolution and throughout the brain, during behavior [5–7]. In this study, we used two-photon (2P) calcium imaging to examine how perception of prey-like visual cues leads to initiation of hunting.

In larval zebrafish, prey catching is a visually guided behavior [8–10]. Several studies have examined the locomotor and oculomotor components of hunting routines including the kinematic features of orienting turns (described as J-turns in [9]), capture swims [8, 11], and coordinated pectoral fin movements [12]. Of particular relevance to this study, zebrafish larvae perform a specialized oculomotor behavior, eye convergence, specifically during hunting. A convergent saccade defines the onset of all hunting routines, and the eyes maintain a high vergence angle until after the strike at prey [13]. After the initial convergent saccade, vergence angle further increases during prey tracking, in relation to target proximity [11]. By increasing the extent of the binocular visual field and advancing it close to the nose of the animal, eye convergence might enable a stereopsis mechanism for judging target distance and triggering the final capture event [13].

The optic tectum (OTc) is the largest retinorecipient structure in the brain of teleost fish and is likely to be of central importance for hunting behavior. Visual space is retinotopically mapped across the OTc in register with the tectal motor map and as such the OTc is well suited to control goal-directed behaviors toward specific points in space [14]. These include orienting and avoidance behaviors [15], saccadic eye movements [16], and prey-catching behaviors including striking at prey [17]. Indeed, neural activity in the OTc of larval zebrafish was recently observed in response to live prey [18]. Zebrafish hunting is greatly reduced by ablating the retinal input to the tectum [10], silencing a specific population of tectal interneurons [19], or a genetic mutation that disrupts the spatial and temporal fidelity of retinotectal transmission [20]. Larvae respond to prey located within the frontal region of visual space (the “reactive perceptive field” [13]), which is represented in the anterior portion of the visuotopic tectal space map [14, 21]. Notably, optogenetic stimulation of the anterior-ventral OTc is sufficient to evoke convergent saccades and J-turns [22]. By contrast, projection neurons in the posterior tectum have been reported to be dispensable for prey catching [19].

In this study, we performed functional imaging in the anterior tectum of tethered larval zebrafish, while the animal engaged in virtual hunting behavior that was evoked by presentation of artificial visual cues [13]. By systematically varying four features of the visual stimuli, we found that prey-catching behavior was selectively evoked by specific conjunctions of visual features. Unbiased clustering of visual response vectors revealed that tectal neurons show mixed selectivity for multiple stimulus features. Furthermore, we could identify cells that showed non-linear mixed feature selectivity that closely matched the stimulus tuning of hunting responses. To investigate how activation of these feature-analyzing neurons might be linked to initiation of prey-catching behavior, we compared neural activity in response trials versus non-response trials. This enabled us to uncover tectal population activity that was specifically associated with hunting responses. Assemblies of tectal neurons produced bursts of activity in advance of, or concurrent with, the initiation of behavior, were confined to the left or right tectal hemisphere and their laterality correlated with asymmetries in the oculomotor parameters of convergent saccades. Consequently, these population dynamics likely represent premotor activity controlling the release of hunting responses. In summary, by imaging neural activity at cellular resolution during naturalistic behavior, we have functionally identified circuit components that are likely to mediate the perceptual recognition of ethologically relevant stimuli and the release of an adaptive behavioral response.

## Results

### Functional Calcium Imaging during Tethered Hunting Behavior in Larval Zebrafish

**Figure 1:**
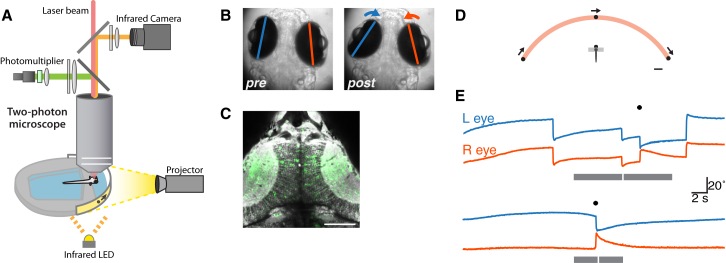
2P Functional Calcium Imaging during Virtual Hunting Behavior in Larval Zebrafish (A) Schematic of experimental setup. Larval zebrafish are tethered in agarose gel but able to freely move their eyes and tail. Visual stimuli are presented by projection onto a miniature screen in front of the animal. A 2P microscope is used to image fluorescent calcium signals, and eye position is monitored simultaneously through the microscope objective using an infrared camera. (B) Eye position recorded before (left) and after (right) a predatory convergent saccade, during 2P imaging. (C) Neural activity recorded in the optic tecta of a Tg(elavl3:GCaMP5G) transgenic larva. The fractional change in fluorescence (Δ F/F) is shown in green (arbitrary color scale) overlaid on an anatomical projection of the focal plane (gray). This field-of-view corresponds to FOV2 as shown in Figure 3A. Dorsal view, anterior top. Scale bar, 50 μm. (D) Schematic of the behavioral assay (viewed from above). The animal is presented with visual stimuli that are projected onto a screen covering ∼200° visual space. Scale bar, 2 mm. (E) Examples of eye-position records from two trials in two different larvae. The gray bar indicates the period during which the visual cue sweeps across visual space from +100° right to −100° left (top) or left-right (bottom). The different lengths of the bars correspond to different speeds of stimulus motion. The white tick indicates the time when the cue is at 0°, directly in front of the animal. The black symbol indicates the automatic detection of a convergent saccade. Downward deflection of eye position traces corresponds to clockwise eye rotation.

To monitor neural activity during the recognition of prey-like visual cues and the initiation of hunting routines, we combined a virtual hunting assay for tethered larval zebrafish [13] with in vivo 2P functional imaging in transgenic larvae expressing a genetically encoded fluorescent calcium indicator under the control of a pan-neuronal promoter, Tg(elavl3:GCaMP5G)a4598 [23] (Figure 1).

In our assay, larval zebrafish were tethered in agarose gel but able to freely move their eyes and tail, and visual cues were projected onto a diffusive screen in front of the animal (Figures 1A and 1D). We previously showed that larvae respond to simple moving spots with hunting-associated oculomotor and locomotor behaviors [13]. Specifically, larvae perform a convergent saccade and an orienting turn, comprising multiple unilateral tail bends directed toward the visual target. The kinematics of these behaviors closely match those observed for freely swimming fish hunting live prey. Because every hunting routine (for both free swimming and tethered larvae) commences with eye convergence, and the spontaneous rate of convergent saccades is very low, we could use eye tracking alone to determine that the animal has initiated hunting behavior in response to a visual target (Figures 1B and 1E). Furthermore, the relatively high failure rate of stimulus-evoked hunting responses (5%–10% response rate for the best stimuli) allowed us to disambiguate visually evoked activity from neural activity related to the release of a behavioral response.

During the virtual hunting assay, we performed 2P calcium imaging to monitor neural activity in the optic tecta (Figure 1C). Hunting responses generated only small motion artifacts that could be corrected during post-processing (see the Experimental Procedures), and 2P imaging had no apparent detrimental impact on larval health or behavior. This approach therefore enabled us to monitor neural activity during the sensorimotor transformations linking the recognition of prey-like visual objects to the initiation of a hunting response.

### Virtual Hunting Assay

To examine the stimulus tuning of hunting responses, we presented a panel of moving spot stimuli that differed in terms of four stimulus features: direction, size, speed, and contrast polarity. For each feature, we tested two values, for a total of 16 unique stimuli. Specifically, moving spots could differ in direction (left-right or right-left motion), speed (fast 30°/s or slow 15°/s), size (small 3.5°, large 13.2°), or contrast polarity (a bright spot on a dark background, or a dark spot on a bright background) (see the Experimental Procedures). Stimuli were presented in the frontal portion of visual space, spanning the region where freely swimming larvae attend to live prey (approximately −60° (left) to +60° (right) [13]). Thus, moving spots appeared at 100° to the left or right of the animal’s extended midsagittal plane and then swept 200° to the right, or left, respectively.

**Figure 2:**
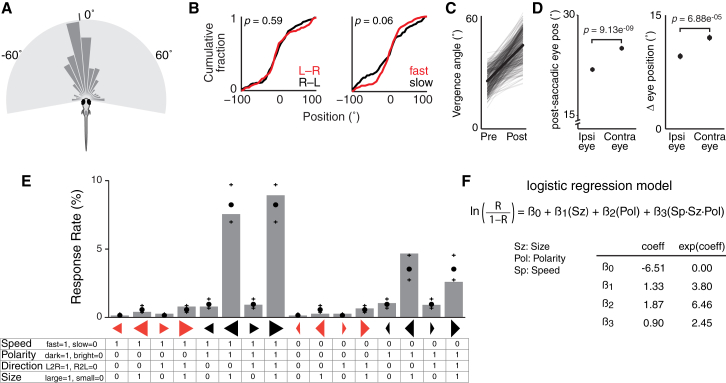
Hunting Responses Show Mixed Selectivity for Stimulus Feature Compounds (A) Distribution of spot locations at time of convergent saccade (n = 361 events in 48 fish). Tick indicates median location, −5.56°. (B) Distribution of spot locations at time of convergent saccade did not differ for left-right versus right-left stimuli (left) nor for slow versus fast stimuli (right). Note that, to compare slow versus fast stimuli, all spot locations were simulated as moving left-right. (C) Change in ocular vergence angle during convergent saccades. Thick line shows mean. (D) The eye contralateral to the visual hemifield in which the spot was located at the time of the convergent saccade showed a greater change in eye position (nasal rotation). Left: post-saccadic eye position. Greater values indicate more nasal position. Right: change in eye position. Data are shown as mean ± SEM. (E) Response rates for the 16 moving spot stimuli (236 events in 27 fish). Black spots indicate response rates predicted by the logistic regression model (crosses indicate 95% confidence interval [CI]). Symbols below the x axis indicate the features of each moving spot stimulus: leftward arrow, right-left; rightward arrow, left-right; large symbol, large; small symbol, small; elongated arrow, fast; short arrow, slow; red, bright; black, dark. The table indicates the binary coding scheme by which each stimulus is coded in terms of four feature values. (F) Logistic regression model that best explained the variance in response rate, *R*, as a function of stimulus features. The inset table shows the estimated values of the coefficients as well as the exponentiated coefficients (also known as odds ratios). The fit coefficients (with 95% CI and p values) were β_0_ = −6.51 [−7.10, −5.92], p = 3.13 × 10^−127^; β_1_ = 1.33 [0.87, 1.80], p = 2.72 × 10^−10^; β_2_ = 1.87 [1.34, 2.39], p = 1.48 × 10^−14^; β_3_ = 0.90 [0.54, 1.24], p = 3.22 × 10^−8^.

Behavioral responses indicating the initiation of hunting routines were defined as convergent saccades in which both eyes rotated nasally (Experimental Procedures) (n = 361 events in 48 fish). Larvae responded to stimuli most frequently when they were almost directly ahead (median azimuth, −5.56° [left], Wilcoxon signed rank test versus median of 0°, p = 0.19; Figure 2A). There was no significant difference in the spatial location of targets at the time of convergent saccades for left- versus rightward-moving spots (p = 0.59, Kolmogorov-Smirnov test; Figure 2B), nor for slow- versus fast-moving spots (p = 0.06).

Convergent saccades increased ocular vergence angle by 19.03° ± 0.49° (mean ± SEM), with mean vergence angle after saccade of 44.4° ± 0.43°, similar to our previous study [13] (Figure 2C). The eye contralateral to the stimulus at the time of the saccade tended to show a larger nasal rotation and adopted a more nasal post-saccadic position (in agreement with [24]). For example, convergent saccades triggered by visual cues located on the right, usually involved greater rotation of the left eye (p = 9.13 × 10^−9^, contralateral versus ipsilateral eye position after the saccade; p = 6.88 × 10^−5^, contralateral versus ipsilateral change in eye position, paired t tests; Figure 2D).

In summary, our behavioral assay allowed us to present visual cues to tethered larval zebrafish to evoke oculomotor responses associated with the initiation of natural hunting routines, during 2P functional imaging.

### Hunting Responses Are Selective for Visual Feature Compounds

The probability of evoking hunting responses varied substantially across our panel of visual stimuli. We quantified response rate (*R*) as the proportion of stimulus presentations that evoked a convergent saccade (Figure 2E). The most effective stimuli were spots for which polarity was inverse (dark spots) and size was large. Fast-moving large, dark spots were also more effective than otherwise identical slow-moving stimuli. These results suggest that hunting responses are sensitive to multiple stimulus features.

We used logistic regression to model the relationship between response rate and the four visual features. For each type of feature, we used a binary coding scheme to represent the two feature levels (e.g., fast [1], slow [0]) such that each stimulus was described by a vector of four binary values (Figure 2E, bottom table). Using stepwise regression, we identified the model described in Figure 2F as producing the most accurate description of the data. To compare alternative models, we used a cross-validation approach in which we fit model coefficients on half the data set (training set) and assessed model predictions against the other unseen half (test data set) to estimate a cross-validated R^2^ (Experimental Procedures). The model in Figure 2F had a cross-validated R^2^ of 0.82 and indicates that hunting responses are strongly modulated by size and contrast polarity. Large stimuli increase the odds of response by 3.8-fold (given by e^β1^) and dark stimuli by 6.5-fold (e^β2^). In addition, the interaction term in the model indicates that, when the stimulus is both dark *and* large *and* fast, the odds of a response are increased by 2.5-fold (e^β3^).

We conclude that larval zebrafish respond differentially to moving visual cues as a function of multiple stimulus features and are sensitive to the coincidence of particular feature values (feature compounds). Specifically, size, contrast polarity and speed of motion interact, such that stimuli that are large, dark, and fast are most effective in triggering hunting responses.

### Visual Response Properties in the Optic Tectum and Adjacent Regions

**Figure 3:**
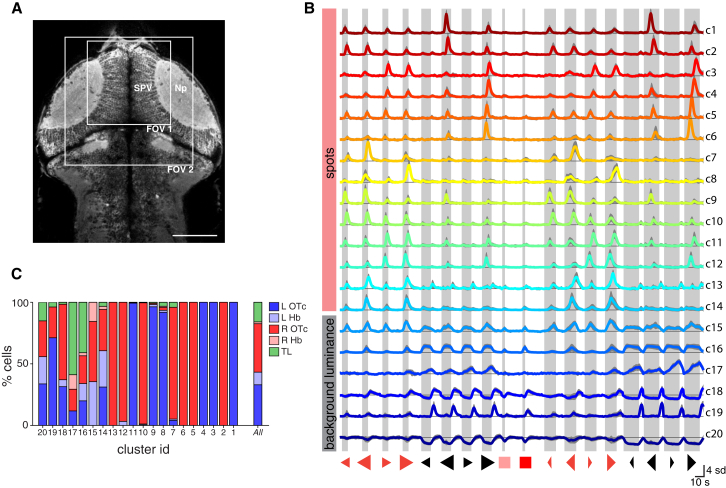
Clustering of Visual Response Properties (A) 2P focal plane showing a dorsal view of the brain of a 5 dpf Tg(elavl3:GCaMP5G) larva. Boxes indicate size and approximate locations of fields of view for functional imaging of the anterior optic tecta. The stratum periventriculare and synaptic neuropil regions of the right OTc are labeled. Anterior top. Scale bar, 100 μm. (B) Cluster centroids (mean visual response vectors) of 20 clusters of visually responsive neurons from 14 fish. For each cluster, the visual response vectors of constituent cells were divided by their SD (to normalize responses across cells with varying magnitudes of signal modulation), and the mean visual response vector was computed (colored lines). Thin black lines indicate zero ΔF/F. Gray shading indicates SD across cells. Numbers on the right are cluster IDs. Shaded bars indicate visual stimulus presentation periods. (C) Anatomical distribution of cells from each cluster. L, left; R, right; Hb, habenula; OTc, optic tectum; SPV, stratum periventriculare; Np, tectal neuropil; TL, torus longitudinalis. See also Figures S1–S3 and Table S1.

To investigate how different stimuli—and individual stimulus features—are encoded by neural activity, we performed 2P calcium imaging in the rostral portion of the optic tecta (and adjacent regions) (Figure 3A). In addition to the 16 moving spot stimuli, we included two control stimuli, which were 3 s “whole-field” light flashes at two different intensities. We imaged activity at ten to 15 dorsoventral levels and at each focal plane presented five to eight repetitions of each of the 18 stimuli, in a pseudo-random sequence, while simultaneously monitoring behavior.

To characterize the visual response profiles of individual neurons, we computed a visual response vector for each cell as follows. First, imaging planes were automatically segmented to define regions-of-interest that corresponded well to single somata (Figure S1). Regions of interest (ROIs) localized to the synaptic neuropil layers of the OTc were excluded. Next, we computed the mean fluorescent calcium signal (ΔF/F) across the repeated presentations of each visual stimulus and finally concatenated these average responses to produce a visual response vector (VRV). The VRV therefore summarizes the visual responses of each neuron in the form of the full response time course to the 18 visual stimuli (684 time points per cell).

To examine the diversity of visual response profiles, we used an unbiased clustering method to group visually responsive cells from 14 fish based on the similarity of their VRVs, as measured by correlation (Experimental Procedures). Our method produced 20 clusters, each of which contained cells from a minimum of six fish (Figure 3B; Figure S2; Table S1). These clusters contained neurons with more coherent visual tuning properties than we could obtain using *k*-means clustering. From a total of 169,371 ROIs (14 fish), our method clustered only 5,092 visually responsive cells (∼3%). This relatively small sample set is most likely not exhaustive but allowed us to identify groups of neurons with feature selective visual tuning that were found consistently across multiple fish. Notably, an alternative clustering approach based on Gaussian mixture modeling identified very similar clusters but also isolated only a relatively small number of cells (1,035 cells from 101,656 in 10 fish, ∼1%; Figure S3; Experimental Procedures). Figure 3B shows the 20 clusters identified using our correlation-based clustering approach to measure the similarity of VRVs, at a minimum correlation coefficient threshold of 0.75 (see Figure S2 and Table S1 for additional cluster details).

**Figure 4:**
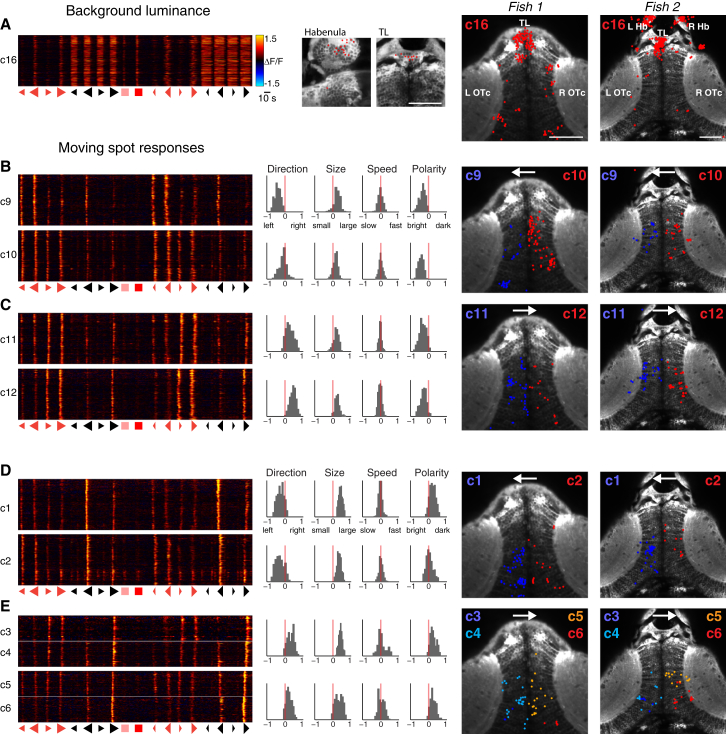
Clusters Respond to Background Luminance Changes and Show Mixed Selectivity to Multiple Features of Moving Spot Stimuli (A) Cluster 16 shows positive modulation in response to increases in whole-field luminance. The left panel shows VRVs for every cell in the cluster (each row corresponds to one cell). Neurons show increased calcium signals in response to the increased background luminance that occurs in conjunction with presentation of dark, moving spots, as well as in response to large bright spots and whole-field light flashes. Middle panels show locations of cluster 16 cells in the habenula and torus longitudinalis, and right panels show anatomical locations of all detected cluster 16 neurons in two representative larvae. Neuron locations are marked as x-y centroids (colored spots) overlaid on a single anatomical image from the dorsoventral mid-point of the imaging volume. Many cluster 16 cells are located in the torus longitudinalis and the habenulae. Scale bars, 50 μm. (B and C) Mirror-symmetric pairs of clusters showing a net preference for large, bright, moving spots. (B) Clusters 9 and 10 are tuned to leftward-moving stimuli and localize to the left and right optic tecta, respectively. (C) Clusters 11 and 12 are tuned to rightward-moving stimuli. Histograms show distributions of feature selectivity indices for cells in each cluster. Arrows on anatomical maps indicate preferred direction of motion. (D and E) Clusters responding to large, dark, moving spots. (D) Clusters 1 and 2 are tuned to leftward-moving large, dark spots. (E) Clusters 3–6 prefer rightward-moving stimuli. These clusters were divided based on differential response times, corresponding to different spatial receptive field locations. Accordingly, they occupy different positions in the tectal space map.

Clusters could be broadly divided into those modulated by changes in background luminance and clusters selective for moving spots. A step increase in background luminance occurs during presentation of negative polarity (dark) moving spots (starting 2 s before spot appearance and ending 2 s after spot disappearance; Experimental Procedures), as well as during the control light-flash stimuli. The six clusters responsive to changes in background luminance (c15–20) showed a diversity of response properties and temporal dynamics. These include negative modulation (a decrease in fluorescence signal, which we presume corresponds to a decrease in tonic firing rate) in response to an increase in luminance (cluster 20): constituent cells were found in the habenulae and torus longitudinalis (TL) as well as the optic tecta (Figure 3C). Cluster 19 showed positive modulation in response to decreases in luminance (“dimming detectors”), and cluster 16, which contained the largest number of neurons of any cluster, displayed positive modulation in response to increasing whole-field luminance (“ON” response). This was evident in the response to changes in background light level during control stimuli and dark spot presentations and in response to large, bright, moving spots. A large proportion of these neurons (41%) were located in the TL, specifically at its rostral pole (Figure 4A).

### Tectal Neurons Are Selective for Multiple Stimulus Features

We identified 14 clusters that were responsive to moving spots and showed minimal modulation to changes in background luminance. Inspection of cluster centroids (the average VRV of cells within the cluster) revealed that clusters respond differentially across the panel of 16 moving spot stimuli and show direction, size, and polarity selectivity (Figure 3B; Table S1). We quantified feature tuning by computing, for each cell, four selectivity indices (for direction, speed, size, and polarity) based on the maximal mean calcium signal across the panel of 16 stimuli (Experimental Procedures).

Mirror-symmetric clusters could be identified in the left and right tectal hemispheres, with similar feature tuning. For example, clusters 9–12 show size, polarity, and direction selectivity, with a net preference for large, bright spots moving either leftward or rightward (Figures 4B and 4C). Clusters 9 and 10 prefer right-left-moving spots. Despite otherwise similar tuning, clusters 9 and 10 were segregated because they respond at different times to spots sweeping across the visual field, from +100° (right) to −100° (left). The retinotectal projection is entirely crossed in larval zebrafish such that the left OTc is innervated by retinal ganglion cells deriving from the right eye and the right OTc receives input from the left eye. Accordingly, cluster 9 is exclusively located in the left OTc and responds earlier during presentation of right-left visual cues (moving tail-nose), whereas cluster 10 is confined to the right OTc and responds later, after the cue has crossed to the left visual hemifield (nose-tail motion; Figure 4B). Clusters 11 and 12 show the opposite direction selectivity (preferring left-right motion) and are similarly located in the left and right OTc, respectively (Figure 4C). Consequently, clusters 9 and 12 form a mirror-symmetric pair tuned to tail-nose motion, located on the left and right, respectively. Clusters 10 and 11 form a second pair tuned to nose-tail-moving spots.

Hunting responses were evoked most frequently by large, dark, fast-moving spots. Our unbiased clustering procedure identified tectal neurons tuned to large, dark spots, which additionally showed direction selectivity. Clusters 1 and 2 show a preference for leftward-moving large, dark spots and are located in the left and right OTc, respectively (Figure 4D). Clusters 3–6 show the opposite direction selectivity, preferring rightward motion. These four clusters have similar tuning and were divided not only by tectal laterality, but also by rostrocaudal tectal location, based on the differential timing of their calcium responses (Figure 4E). In accordance with the retinotopic mapping of visual space, clusters at more caudal positions (clusters 3 and 6) responded when visual cues were at more peripheral locations. Our expectation is that other clusters (e.g., clusters 1 and 2) could be similarly subdivided if temporal resolution was higher or the correlation threshold of our clustering procedure was increased.

In conclusion, we find that tectal neurons show mixed selectivity and are sensitive to combinations of visual features (feature compounds). These include direction-selective cells with a preference for large, dark, moving spots that we found to be among the most effective stimuli in evoking hunting responses.

### Non-linear Mixed Selectivity Neurons Are Tuned to Optimal Prey-like Visual Stimuli

Hunting responses displayed mixed feature selectivity and were most effectively triggered by large, dark, fast-moving spots. Based on these behavioral observations, we developed an approach to specifically search for neurons that could mediate detection of preferred prey-like visual targets. In addition, we sought to quantitatively describe neural activity as a function of the four visual features to determine if individual neurons are selective for the conjunction of multiple features in a similar way to whole animal behavior.

**Figure 5:**
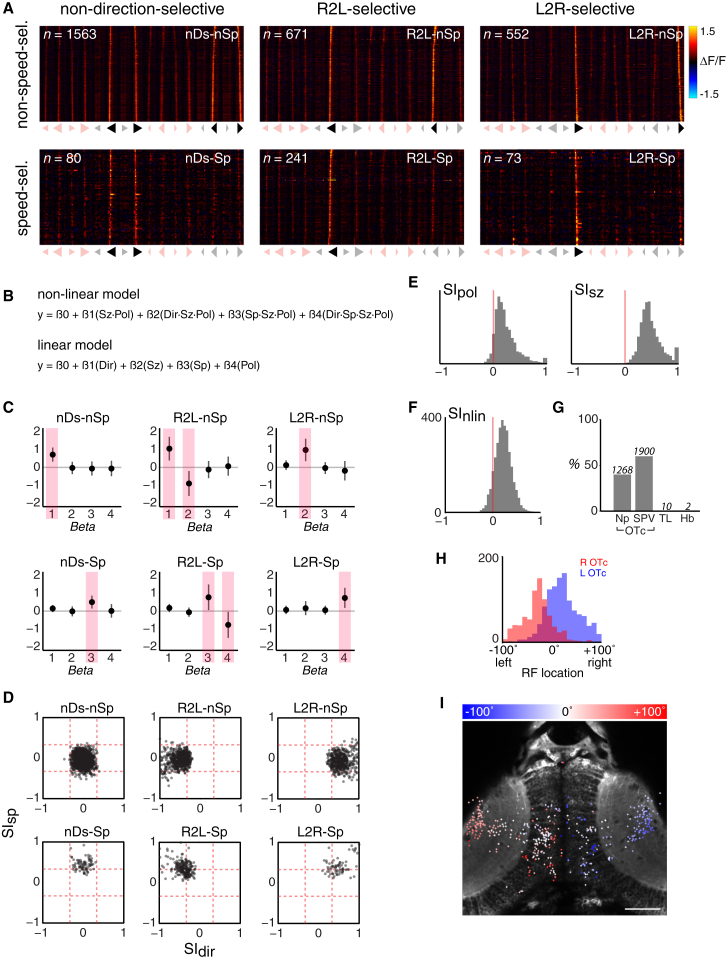
Tectal Neurons Show Non-linear Mixed Selectivity for Best Prey-like Stimuli (A) Visual response vectors (VRVs) of ROIs isolated using six regressors that were designed based upon the stimulus tuning of behavioral response rates. Symbols below each panel indicate the stimuli coded 1 in the binary vector defining the regressor. Stimuli coded 0 are shown in light shading. ROIs were associated with the regressor that produced the highest correlation coefficient, when that coefficient was 0.75 or greater. (B) Non-linear and linear models used to fit the response profiles of individual ROIs. *y* represents the fluorescence response (peak ΔF/F during stimulus presentation). Each stimulus is coded as a set of four binary feature values, as shown in Figure 2E. (C) Coefficients (β’s) derived from fitting the non-linear model in (B) to ROIs associated with each regressor. Shaded bars indicate coefficients expected to show significant non-zero values. Data are shown as mean ± SD. (D) Direction- and speed-selectivity indices (*SI*_*dir*_*, SI*_*sp*_) for ROIs associated with each regressor. (E) Polarity- and size-selectivity indices (*SI*_*pol*_*, SI*_*sz*_). (F) Comparison of model fits (*R*^2^) obtained with non-linear versus linear mixed selectivity models, quantified as a selectivity index, *SI*_*nlin*_. The majority of ROIs show a positive index, indicating the non-linear model provides a more accurate description of the variance of their responses. (G) Anatomical distribution of ROIs. Values above each bar indicate number of ROIs. (H) Distribution of receptive field (RF) centers for NLMS neurons in the left (blue) and right (red) optic tecta. Only ROIs localized to the tectal SPV are shown. (I) Anatomical map of all NLMS ROIs in one example fish. Each spot indicates the centroid of an ROI, color-coded according to estimated RF location. ROIs located in both the tectal SPV and neuropil regions are shown. Note that for presentation, ROIs from the entire imaging volume are overlaid on a single anatomical image from the dorsoventral mid-point of the volume. Scale bar, 50 μm. See also Figure S4.

In the first part of our approach, we designed a panel of simple binary “regressors” to search for cells with visual tuning profiles similar to the behavioral tuning. All regressors were selective for large, dark stimuli. Although behavioral response rates do not show net direction selectivity, we recognized that this could result from the combined action of two or more populations of direction-selective neurons that mediate responses to prey moving in different directions. Importantly, we found the clusters with preference for large, dark spots to be direction selective (see above). Therefore, we included regressors that either did or did not include this property. In the logistic regression model describing response rate in terms of stimulus features, the interaction term involving speed had the smallest coefficient. For this reason, as well as the fact that the unbiased clustering did not isolate speed-tuned cells, we designed regressors with and without speed tuning. The six regressors were as follows: non-direction selective, non-speed selective (nDS-nSp); non-direction selective, speed selective (nDS-Sp); leftward selective, non-speed selective (R2L-nSp); leftward selective, speed selective (R2L-Sp); rightward selective, non-speed selective (L2R-nSp); and rightward selective, speed selective (L2R-Sp) (Figure 5A).

Next, we used these regressors to identify ROIs with similar stimulus tuning. We considered ROIs located in the tectal neuropil as well as those corresponding to cell bodies in the stratum periventriculare (SPV) and regions adjacent to the OTc. For every ROI, we computed the correlation coefficient between a vector describing the peak average response to each of the 16 moving spot stimuli and each of the regressors. ROIs were associated with the regressor that yielded the highest correlation coefficient when that coefficient was 0.75 or greater.

Figure 5A shows the VRVs for all of the ROIs that were associated with each of the six regressors, from ten fish. Visual inspection of the VRVs indicates that the isolated cells are tuned to large, dark, moving spots. More ROIs were isolated by the non-speed-selective regressors and the largest number was associated with the nDS-nSp regressor. However, an appreciable number of ROIs showed selectivity for fast motion, responding most strongly to the large, dark, fast-moving stimuli. Notably, these highly stimulus-selective tuning profiles were apparent for single voxels from such ROIs (Figure S4).

To quantitatively describe these tuning profiles, we used generalized linear regression to model the responses of each ROI as a function of stimulus features. We used cross-validation to compare two models: a linear mixed selectivity model and a non-linear mixed selectivity model with interaction terms designed to capture the responses of neurons tuned to large, dark, moving spots, which may or may not also display direction selectivity and/or speed tuning (Figure 5B). For the majority of ROIs, stimulus tuning was better described by the non-linear mixed selectivity model (for 86% ROIs, cross-validated R^2^_nlin_ was greater than R^2^_lin_, *p* « 0.0001, Wilcoxon signed rank test). We further quantified this by computing an index, SI_nlin_, based on the relative ability of the linear and non-linear models to explain the variance of the responses (Experimental Procedures). The superior performance of the non-linear model was true for the majority of ROIs associated with each of the six regressors (Figure S4B) as well as for the entire population pooled across regressors (Figure 5F). Responses to the 16 stimuli predicted by the non-linear model showed good agreement with measured values (Figure S4A).

Figure 5C shows the non-linear model coefficients obtained for ROIs associated with each regressor. Coefficients for the model terms that are expected to define the response properties of each group show non-zero values of the expected sign. For example, neurons selective for large, dark spots but which are neither speed nor direction selective, should be well described by the *size^∗^polarity* interaction term. Indeed, for the population of nDS-nSp cells, only this coefficient shows a non-zero value. By comparison, for right-left-tuned cells that also show a preference for fast spots (R2L-Sp), the *speed^∗^size^∗^polarity* term should have a positive coefficient (such that the cells respond to fast (1), large (1), dark (1) stimuli that are encoded as 1’s in our binary coding scheme; Figure 2E). So that direction selectivity can be conferred, the *direction^∗^speed^∗^size^∗^polarity* term should also be non-zero, with a negative coefficient, to suppress responses to rightward-moving stimuli (rightward motion is coded as 1). This is what we observed. We conclude that our method identified ROIs showing non-linear mixed selectivity (NLMS), tuned to conjunctions of multiple feature values. Almost all of these ROIs (99.6%) were located in the tectal cell body layers or neuropil, with very few in adjacent structures (habenula, TL) (Figure 5G).

Selectivity indices, for the four individual stimulus features, confirmed that NLMS ROIs preferentially respond to large, dark, moving spots (Figure 5E), and ROIs associated with each regressor showed the expected pattern of speed- and direction selectivity (Figure 5D). After correcting for eye position, we estimated the spatial receptive fields (RFs) of individual NLMS neurons in the tectal SPV and found that they spanned the frontal region of visual space, with a high density of RF centers in the central region where visual cues evoked the highest proportion of hunting responses (Figures 5H and 5I).

In summary, the OTc contains highly tuned neurons displaying non-linear mixed selectivity for specific conjunctions of visual features. We identified NLMS neurons that preferentially respond to large, dark, fast-moving spots, which are the visual stimuli that were most effective in evoking hunting responses. These neurons are therefore candidates for mediating the perceptual recognition of optimal prey-like visual objects.

### Tectal Activity Associated with Initiation of Hunting Behavior

What are the neural substrates linking sensory perception of prey-like cues to the release of prey-catching behavior?

**Figure 6:**
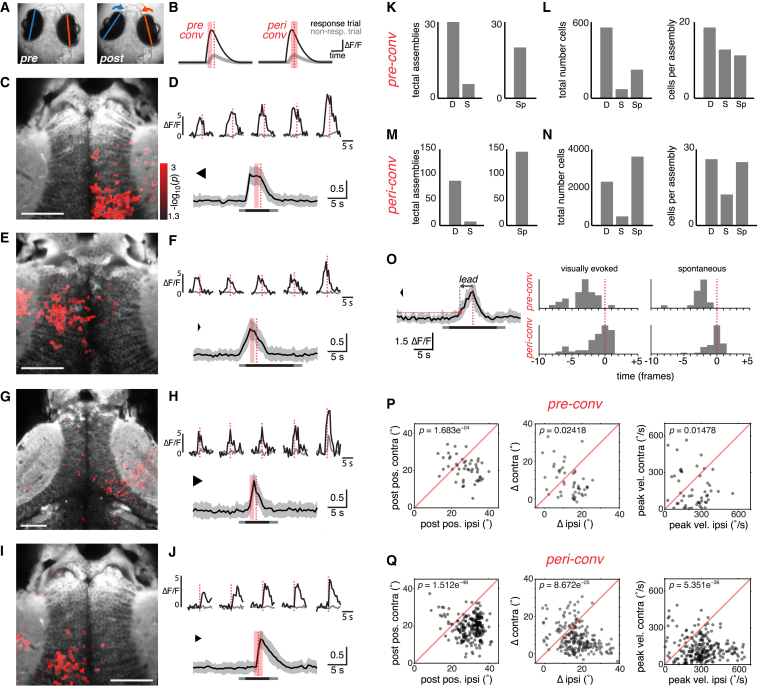
Assemblies of Tectal Neurons Show Premotor Activity Associated with Convergent Saccades (A) Eye position before and after a convergent saccade. (B) Schematic indicating time windows used to identify ROIs with a significantly greater GCaMP response immediately prior to a convergent saccade (“pre-conv”, left) or around the time of the saccade (“peri-conv”, right). Dashed red line indicates time of eye convergence in response trial. Shaded red bars indicate time windows during which activity (ΔF/F) was compared (t test) between response (black) versus non-response trials (gray). (C) Example of right tectal assembly that was active in advance of a convergent saccade (“pre-conv”). Response-modulated ROIs (red, color map indicates log-transformed p value from t test) are overlaid on an anatomical projection (gray). (D) Top: five cells from the assembly in (C). Activity in response trial in black, mean activity in non-response trials in gray. Bottom: activity of all cells in assembly in (C) during the response trial. To facilitate comparison of cells with different response amplitudes, we normalized ΔF/F time courses to the maximum value for each cell before computing the population average, shown as mean ± SD. Bar indicates visual stimulus presentation (light gray, change in background luminance; dark gray, moving spot presentation). (E–H) Two more examples of “pre-conv” assemblies that were active in advance of convergent saccades. Assemblies are from two different fish that are also different from (C) and (D). (I) Left tectal assembly that was active around the time of a convergent saccade (“peri-conv”). Same fish as (C) and (D). (J) Responses of individual cells (top) and the whole assembly (bottom) shows activity coincident with, or immediately following, the convergent saccade. (K) Left: number of pre-conv assemblies identified in original data, “D” and after circular permutation of the timebase (shuffling, “S”). We estimated false discovery rate to be 19%. Right: number of pre-conv assemblies associated with spontaneous convergent saccades, “Sp”. (L) Total number of cells (left) and mean number of cells per assembly (right) for pre-conv assemblies. (M and N) Data for peri-conv assemblies, as per (K) and (L). False discovery rate was 9%. (O) Left: example illustrating detection of onset of assembly activity. Population activity of a pre-conv assembly is shown (mean ± SD). Horizontal red line indicates threshold. The population response crosses threshold at the time indicated by a red dot, which precedes the saccade by a certain lead time (arrow). Right: histograms of lead times for pre-conv (top) and peri-conv (bottom) assemblies associated with visually evoked (left) or spontaneous (right) convergences. x axis is marked in imaging frames, relative to time of saccade (frame zero). (P and Q) Oculomotor parameters associated with assembly activity. Post-saccadic eye position (left), change in eye position (middle), and peak nasal eye velocity (right) are compared for the eye ipsilateral to the assembly (ipsi) versus the eye contralateral to the assembly (contra). Positive values correspond to more nasal eye positions/rotations. For both types of assembly, the eye ipsilateral to the tectal assembly shows a larger post-saccadic eye position, a larger change in horizontal position and a larger peak velocity. p values obtained by paired t tests comparing ipsilateral versus contralateral eye. Data for visually evoked and spontaneous convergences are combined. See also Figures S5–S7.

We investigated whether tectal activity was associated with execution of the first motor output that defines initiation of hunting behavior, namely, a convergent saccade. To do this, we took advantage of our online behavioral data, which allowed us to track eye movements during 2P functional imaging (Figure 6A). We compared the activity of individual tectal neurons in response trials versus non-response trials to identify cells that showed a significant increase in fluorescence signal associated with the release of hunting responses (Figure 6B). Specifically, we considered two eye convergence-triggered time windows: the “pre-conv” window compared activity during 1.65 s immediately prior to the convergent saccade to activity at corresponding times in non-response trials. This window was designed to identify tectal neurons with premotor activity, which might be involved in initiating hunting responses. The second window, “peri-conv,” compared activity during 2.75 s centered on the convergent saccade. This enables identification of neurons that modulate activity coincident with an eye convergence. For both windows, we used t tests to compare activity (ΔF/F) in response versus non-response trials. Furthermore, we evaluated the maximum signal-to-noise ratio (SNR) of the cell’s response within the same window. We considered cells to show response modulation when p < 0.05 and SNR >3.

### Tectal Assemblies Represent an Activity Motif Associated with Convergent Saccades

Maps of response-modulated cells revealed spatially grouped clusters, or *assemblies*, of tectal neurons located in discrete regions of the stratum periventriculare (SPV) and confined to either the left or right tectal hemisphere. Three examples of assemblies identified from the pre-conv analysis are shown in Figures 6C–6H (see also Figure S6). The assembly shown in Figures 6C and 6D comprises a spatially clustered population of neurons in the right OTc, which shows a burst of activity after visual stimulus onset and immediately preceding execution of a convergent saccade. Many of the neurons showed no detectable activity in response to the same visual stimulus in non-response trials (see examples in Figure 6D). However, all the constituent cells were classed as visually responsive, indicating response modulation to at least one of the 18 stimuli in our test set (Experimental Procedures). Cells within the assembly showed correlated patterns of activity during the response trial. We quantified this by calculating the average correlation of each cell’s fluorescence time course with the mean of the assembly, *r*_avg_ = 0.93 [0.89, 0.95] (median, interquartile range).

We identified 30 pre-conv assemblies from a total of 304 convergence events in 19 larvae. Assemblies were automatically detected by fitting an ellipse to the spatial distribution of response-modulated cells and were defined as unilateral clusters containing a minimum of six cells at a minimal density of 533 μm^2^/cell (Experimental Procedures). Only cells in the tectal SPV were considered for this analysis, and ROIs in the neuropil were excluded. Note, however, that response-modulated ROIs were often observed in the neuropil adjacent to active assemblies (Figures 6C–6J). At the population level, pre-conv assemblies increased activity in advance of convergent saccades by an average of 1.65 s [1.1, 2.2] (median, interquartile range) (Figure 6O). The average intra-assembly correlation of individual cells with the mean assembly response was 0.71 [0.65, 0.81] (median, interquartile range), and the percentage of constituent cells classified as visually responsive was 82% [71, 100] (median, interquartile range) (Figure S5). Notably, pre-conv assemblies contained few, if any, NLMS cells (median = 0, mean = 0.6, n = 223 cells in 14 assemblies) suggesting prey detection and the initiation of predatory responses are mediated by largely non-overlapping neuronal populations in the OTc.

The peri-conv analysis identified tectal neurons that showed significantly elevated activity during response trials within a time window centered on convergent saccades and revealed that such cells were also grouped into anatomically discrete assemblies (88 assemblies from 304 convergent events in 19 larvae). The example in Figures 6I and 6J shows such an assembly in the left OTc. Cells in this assembly predominantly increased activity in the same imaging frame as the convergent saccade, with activity peaking in the subsequent frame. Indeed, this peri-conv analysis allowed us to discover assemblies in which population activity started concurrent with, or subsequent to, the convergent saccade. By manually examining individual imaging movies, we were careful to ensure that this did not result from motion artifacts that escaped our registration procedure. Such post-saccadic activity might represent an efference copy of a saccadic motor command. For peri-conv assemblies, population activity preceded the convergence event by an average of 0.55 s [0, 1.1] (median, interquartile range), with many assemblies showing onset of activity concurrent with or in the first frame after eye convergence (Figure 6O). As with pre-conv assemblies, constituent neurons showed highly correlated activity during the response. The average intra-assembly correlation with the mean response was 0.76 [0.68, 0.82] (median, interquartile range). Again, the majority of constituent cells were classified as visually responsive: 89% [69, 100] (median, interquartile range).

To assess whether tectal assemblies are specifically associated with convergent saccades or could represent spontaneous ongoing activity that occasionally coincides with hunting responses, we estimated a false discovery rate. To do this, we constructed artificial response and non-response epochs by circularly permuting the fluorescence time-series data and detected assemblies using identical criteria to those used for the original data. From the average of five permutations, we estimate false discovery rate to be approximately 10%–20% (18.7% pre-conv and 8.9% peri-conv assemblies) (Figures 6K and 6M). We conclude that the vast majority of tectal assemblies we detect are associated with convergent saccades.

Larval zebrafish perform “spontaneous” convergent saccades, in the absence of an obvious visual stimulus, at very low frequency (1.89 ± 0.46/hr, mean ± SEM, range, 0–7.1/hr). However, over the full course of our imaging experiments, we collected data for a number of spontaneous convergences. This allowed us to examine whether these events were also associated with activation of OTc assemblies, which might be expected if assembly activity represents premotor activity upstream of oculomotor outputs. Using the same convergence-triggered time windows (pre-conv and peri-conv), we compared the fluorescence response of individual cells within the window to the mean signal during the remainder of the 32 s epoch. Although this analysis differs to that used for visually evoked convergences (where we could compare activity within corresponding time windows in response versus non-response trials), we found that spontaneous events were also associated with tectal assemblies (20 pre-conv assemblies and 145 peri-conv assemblies from 278 spontaneous convergences in 19 larvae; Figures 6K–6N). These assemblies had similar properties to those identified for visually evoked hunting responses. For spontaneous convergences, pre-conv assemblies increased activity in advance of convergent saccades by 1.1 s [1.1, 1.65], contained 81% [72, 100] visually responsive cells and average intra-assembly activity correlation was 0.68 [0.56, 0.82]. For spontaneous peri-conv assemblies, activity increased 0 s [0, 0.55] before eye convergence, 89% [67, 100] were visually responsive and intra-assembly activity correlation was 0.72 [0.65, 0.78] (see also Figure S5). In summary, these data suggest that assembly activation represents motor-correlated activity that is not directly, or obligately, downstream of visual input.

As expected from the design of the windows, pre-conv and peri-conv assemblies tended to overlap. We detected a greater number of assemblies with the wider peri-conv window, and the majority of convergences for which we identified a pre-conv assembly were also associated with a peri-conv assembly (77% and 85% for visually evoked and spontaneous convergences, respectively). Moreover, for visually evoked convergences, there was substantial overlap in constituent neurons: 66% of cells in pre-conv assemblies were also components of peri-conv assemblies (Figure S5).

### Locations of Tectal Assemblies Are Related to Motor Parameters of Convergent Saccades

During the convergent saccade that initiates hunting routines, eye movements are often asymmetric, with the eye contralateral to the stimulus showing a greater nasal rotation on average (Figure 2D). We examined whether the laterality of tectal assemblies was associated with asymmetries in oculomotor parameters. For both pre-conv and peri-conv assemblies, the laterality of the assembly (left or right tectal location) corresponded to the eye that showed the more nasal post-saccadic position, the larger change in eye position (nasal rotation), and the greater peak eye velocity (Figures 6P and 6Q). Thus, activation of a left tectal assembly is associated with the left (ipsilateral) eye showing a larger, faster rotation and adopting a more nasal eye position than the right (contralateral) eye. This result is compatible with tectal assemblies causing asymmetric activation of extraocular motoneurons, so as to produce greater convergence of the ipsilateral eye.

The alignment of sensory and motor maps in the OTc is a characteristic of all vertebrate species studied such that activation of distinct tectal sites can produce goal-directed movements toward spatially localized sensory cues [25]. Different points along the anterior-posterior axis of the OTc correspond to different points in visual azimuth, and so we predicted that assemblies at more caudal tectal locations, corresponding to peripheral target locations, might be associated with more asymmetric convergent saccades. Specifically, vergence of the left eye should be greatest when assembly activation occurs at caudal locations in the left OTc (corresponding to the peripheral right visual field) and decline in the sequence left-caudal > left-rostral > right-rostral > right-caudal. The opposite relationship is expected for the right eye. We estimated the location of each assembly by measuring the distance of its center of mass from the posterior commissure and observed the expected trends in oculomotor parameters as a function of assembly location (Figure S7). Independent straight line fits to data for each tectum usually showed the expected positive (right eye) or negative (left eye) slope, but in the majority of cases did not achieve statistical significance. This is likely due to variation between animals producing an additional source of unexplained variance; we did not detect sufficient assemblies to assess motor mapping within individual fish. However, these trends support the possibility that assemblies conform to a motor map that directs convergent saccades toward target locations.

In summary, by imaging neural activity during behavior we were able to identify a reproducible pattern of population activity in the OTc that is associated with the release of prey-catching behavior.

## Discussion

### Model for Initiation of Zebrafish Hunting Behavior

**Figure 7:**
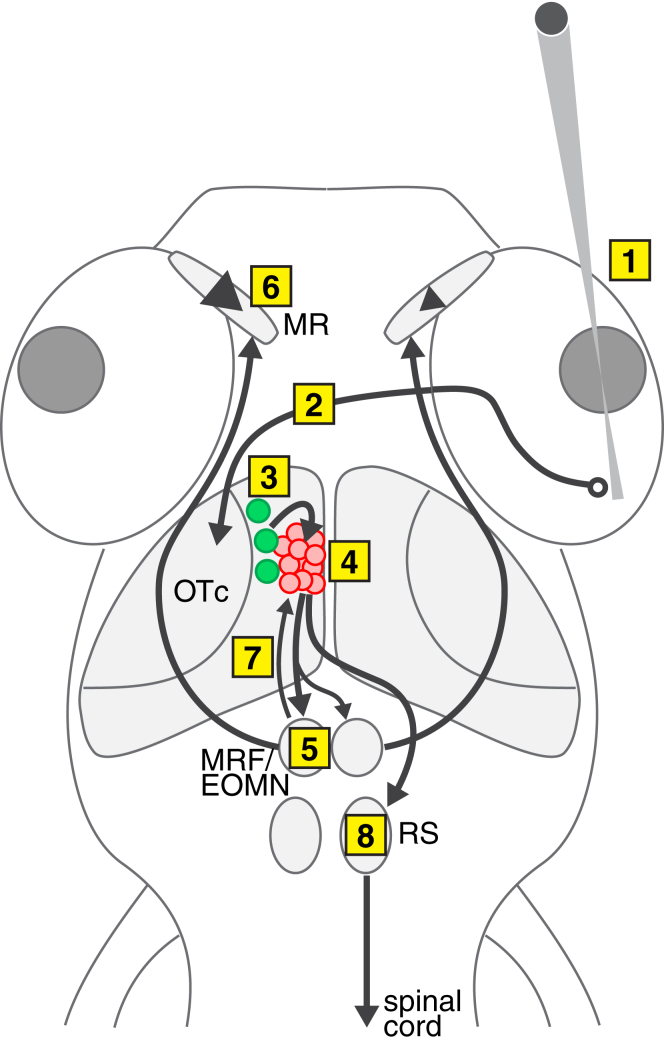
Model Circuit for Visual Prey Recognition and Release of Hunting Responses (1) An image of a prey-like visual stimulus, in this case in the right visual hemifield, is cast on the right temporal retina. (2) Visual information is transmitted to retinal ganglion cell arborization fields in the diencephalon and midbrain of the contralateral (left) hemisphere. (3) Non-linear mixed selectivity neurons (green), selective for combinations of visual features that characterize optimal prey-like stimuli, are activated in the (left) rostral tectum. (4) NLMS neurons recruit the activity of tectal assemblies (red). (5) Correlated bursting of tectal assemblies activates circuits in the mesencephalic reticular formation (MRF), which control a saccadic motor program involving activation of extraocular medial rectus motoneurons (EOMNs) in the oculomotor nucleus. OTc efferents project to the MRF with an ipsilateral (in this case left) bias. (6) EOMNs control the ipsilateral medial rectus to produce a convergent saccade. The eye ipsilateral to the tectal assembly (Figure 6), and contralateral to the visual stimulus (Figure 2), shows the larger amplitude and velocity of eye rotation (in this case left eye). (7) Reciprocal connections from the MRF to the tectum may underlie peri-conv activity. (8) Tectal assemblies activate reticulospinal (RS) neurons, which, in turn, recruit spinal circuits to produce an orienting turn toward the visual target.

Figure 7 shows a working model of the neural pathway that controls the initiation of zebrafish hunting behavior. We propose that (1) hunting responses are evoked by visual objects characterized by conjunctions of visual features. Under our experimental conditions, size, speed, and contrast polarity interacted to trigger prey-catching behavior. (2) Visual information is transmitted to retinal ganglion cell arborization fields in the diencephalon and midbrain of the contralateral hemisphere [26, 27]. (3) Non-linear mixed selectivity (NLMS) neurons in the OTc function as feature-analyzing cells that mediate prey recognition. NLMS neurons may receive afferent input from retinal ganglion cells, tectal interneurons, and extra-tectal regions. (4) We propose that the activity of single, or multiple, NLMS neurons contributes to the activation of small populations of premotor tectal neurons (*assemblies*). Modulatory inputs to the tectum may also influence the recruitment of assemblies, providing a mechanism for gating the sensorimotor pathway that links prey recognition to behavioral output. Correlated bursting of a tectal assembly provides the premotor command for the release of a hunting response. Motor outputs are mediated by downstream circuits that control saccadic eye convergence and orienting turns/swims. (5) A key efferent target of premotor tectal assemblies is expected to be saccade-generating circuitry in the mesencephalic reticular formation (MRF) [28]. Tectal innervation of the MRF shows an ipsilateral bias [29], which might account for assembly activity being associated with larger, faster movements of the ipsilateral eye. (6) A circuit involving the anterior MRF and extraocular motoneurons (EOMNs) in the oculomotor nucleus, which innervate the medial rectus, would produce a convergent saccade. (7) Reciprocal projections from the MRF to the tectum [29] could underlie peri-conv assembly activity and function in feedback control of eye movements [30] or an efference copy mechanism that contributes to stable perception of prey during oculomotor and locomotor responses. (8) Tectal assemblies are also expected to establish efferent connections with reticulospinal (RS) neurons, which, in turn, control spinal cord circuits to produce goal-directed orienting turns.

### Hunting Responses Are Triggered by Visual Feature Compounds

Using an assay in which prey-catching behavior can be evoked in tethered larvae using synthetic visual cues [13], we found that the features size, speed, and contrast polarity all modulated response rate, and appeared to interact. Our logistic regression model indicates that large stimuli increase the odds of a response by at least 3.8-fold compared to an otherwise identical small spot. This effect of size was unexpected because we previously showed that for freely swimming larvae, stimuli ≤5° produced orienting responses, whereas those ≥10° triggered aversive turns [13], which was subsequently confirmed in tethered larvae [24]. One possible explanation may relate to the absolute size and distance at which the cues are presented. In both previous studies, the screen was substantially further from the animal, and the absolute size of the aversive stimulus was 2- to 8-fold larger than the “large” spot used in this assay. Although it is not known whether larval zebrafish can evaluate absolute size of visual objects, size constancy has been reported in goldfish, including under conditions of monocular viewing [31], and tectal neurons, including those with monocular receptive fields, are sensitive to absolute object size [32]. In our assays, visual cues appear within, or move through, the binocular visual field, potentially enabling the animal to use horizontal disparity information to estimate target distance (and therefore size). Certainly after the onset of hunting, sustained eye convergence suggests larval zebrafish use a simple form of stereopsis for prey range finding [13]. Notably, both angular sizes we tested fall within the range that can initiate natural hunting routines directed toward live prey.

Contrast polarity had the greatest effect on response rate of any of the features we tested, with dark spots increasing response odds by at least 6.5 times compared to bright spots. Furthermore, unbiased clustering identified tectal neurons that were highly selective for dark spots (and showed no detectable modulation to changes in background luminance). Many years ago, Horace Barlow suggested that retinal “off” units, concentrated in the posterior retina of the frog, are well suited to provide accurate information about the position of a fly [33], and RGCs that are responsive to small dark objects (but not bright ones) were proposed to function as “bug perceivers” [34]. In dragonflies, small target motion detector (STMD) neurons only respond to dark, negative-polarity objects [35], and it has been suggested that this selectivity is compatible with dragonflies swooping upward to capture prey that will appear dark against a bright sky. During their final capture swim, larval zebrafish also show dorsal flexion to strike their prey from below, and so selectivity for dark targets might represent an adaptive feature of the visual system for discriminating prey against a relatively bright background.

Larval zebrafish responded most strongly to moving spots that were large, dark, and fast. The interaction term in the model indicates that this conjunction of three characteristics more than doubled the odds of a response (2.5× increase). Thus, prey recognition in larval zebrafish is sensitive to feature compounds. In toads, prey-catching behavior is most effectively evoked by moving objects defined by the conjunction of multiple features, notably size, geometry, and orientation with respect to direction of motion (reviewed in [36]). This has led to the concept that computations that link visual features are central to the visual system’s ability to derive the signal value of potential prey from visual input. It is worth noting that the interaction between size and speed that we observe for larval zebrafish is compatible with natural hunting responses directed toward proximal prey, which will possess similar angular size and velocity. A *Paramecium* 135 μm in size located 0.5 mm away and moving at 0.5 mm/s would appear as a 15° stimulus moving at 50°/s, in decent agreement with our large (13.2°), fast (30°/s) condition.

### Visual Response Properties in the Tectum and Adjacent Regions

To examine how tectal circuits might represent visual features and feature compounds, we imaged neural activity using 2P calcium imaging and developed a clustering procedure that identified coherent groups of neurons with a range of visual tuning profiles.

Several clusters showed differing patterns of response to changes in luminance, including many cells in the habenula and torus longitudinalis (TL), in agreement with other recent imaging studies [7, 37]. In response to luminance increases, habenular cells showed sustained, excitatory responses (cluster 16) or sustained inhibition (cluster 20). In agreement with [37], clusters were lateralized, with more visually responsive neurons on the left.

In other fish species, neurons in the TL are primarily excited by dimming of the contralateral visual hemifield [38]. Surprisingly, the major visual response at the rostral pole of the TL in larval zebrafish was positive modulation after an increase in luminance. Notably, previous recordings in adult goldfish failed to detect visually evoked activity at the most rostral recording site in TL. Therefore, our observations might represent a developmental stage or species difference, or a specialized function of the anterior TL.

Visual response properties have been extensively studied in the superior colliculus/OTc, including in larval zebrafish. In agreement with previous studies [18, 19, 21, 39–42], our functional clusters exhibited direction and size selectivity. In addition, we identified neurons that preferentially responded to bright or dark spots. Although our dark spot condition included an increase in background luminance, neurons that preferred dark spots showed minimal modulation to changes in whole-field luminance and appeared to respond specifically to the dark moving spot. This suggests that neurons in the larval zebrafish tectum display contrast-polarity tuning.

By systematically varying four stimulus features, we discovered that tectal neurons show mixed selectivity to multiple features. We identified mirror-symmetric pairs of clusters with equivalent mixed feature tuning, including four clusters [9–12] that were direction selective with a net preference for large, bright spots and six direction-selective clusters [1–6] with an overall preference for large, dark stimuli. Our clustering procedure was sensitive to the timing of neural activity and consequently segregated cells on the basis of their spatial receptive fields (and anatomical locations in the retinotopic tectal map). The presence of symmetrical clusters having the same nose-tail or tail-nose tuning, but localized to left versus right tectum, implies similar perceptual sensitivity to stimuli moving in different directions in the left and right visual hemifields. This is compatible with our observations [13], and those of others [11], which show that larvae respond to live prey distributed throughout a frontally located cone of visual space. Notably, clusters 7 (right OTc) and 8 (left OTc) comprised a mirror-symmetric pair preferring large, bright spots moving nose-tail in the contralateral visual hemifield. This biased representation of stimuli moving “into” a visual hemifield is similar to observations in the posterior OTc, where a preference for tail-nose-moving spots has been reported [21]. These neurons may be involved in modulating visual processing to compensate for reafferent nose-tail motion signals produced by the animal’s forward swimming movements.

### Non-linear Mixed Selectivity Neurons May Underlie Visual Prey Recognition

Using a combined regression and modeling approach, we identified six groups of cells that were highly selective for the best prey-like stimuli. Their responses were better described by a non-linear model comprising interaction terms defining specific conjunctions of stimulus features as compared to a linear model with an equal number of free parameters. Therefore, we describe these highly selective cells as “non-linear mixed selectivity” (NLMS) neurons.

We suggest that NLMS neurons represent feature-analyzing cells that could underlie the ability of zebrafish to categorize visual objects as prey. How might the different types of NLMS neuron be involved in controlling hunting responses? The largest group was the non-direction-selective, non-speed-selective (nDS-nSp) type. This response property is well suited for detection of large, dark, moving spots but fails to account for the additional preference for fast stimuli that was evident in the behavior. One possibility is that activity of multiple types of NLMS neuron is read out by downstream neurons that, in turn, trigger hunting responses. With appropriate synaptic weights, these downstream readout circuits would have a stimulus tuning profile that matches behavioral response rates. Alternatively, individual NLMS cells might be sufficient to trigger hunting responses. In this case, the overall behavioral tuning would represent the summed contribution of the NLMS population over repeated hunting episodes. Our observation of direction-selective NLMS cells would fit with this second hypothesis. These neurons could evoke hunting responses to prey moving in opposite directions, but their summed activity over time would produce a behavioral tuning profile with no net direction selectivity. Future experiments will be required to test whether the activation of NLMS cells is sufficient to trigger hunting responses and whether the different cell types evoke distinct motor outputs.

The response properties of NLMS cells indicate that the visual system performs logical operations on visual input features, but where do these computations occur? One possibility is that retinal ganglion cell (RGC) afferents already show mixed selectivity that is transmitted to post-synaptic tectal neurons. Alternatively, NLMS might be an emergent property of local tectal processing (e.g., [41]). We could not distinguish between these possibilities because we used a transgenic line in which the tectal neuropil contains a dense mix of labeled RGC axons as well as the dendritic arbors of tectal neurons. In support of the possibility that NLMS emerges in the tectum, it has long been recognized that different feature selective classes of RGCs arborize in different layers within the tectal neuropil [34] and in larval zebrafish, direction-, orientation-, and size-selective RGC axons show laminar segregation [42, 43]. Tectal periventricular neurons (PVNs) in the SPV are monopolar cells that extend their dendrites through the neuropil laminae [44] enabling them to potentially combine inputs from different feature-selective RGCs. The non-linearity inherent to the spike generation mechanism could mediate the non-linear integration of visual features that characterizes NLMS responses. Notably, ROIs in the tectal neuropil were associated with all six types of NLMS. Although this might simply correspond to the activity of tectal neuron neurites, we do not exclude the possibility that mixed selectivity is in part or whole computed in the retina.

In summary, tectal NLMS neurons represent feature detectors that are selective for the conjunction of visual features that define optimal prey-like visual objects. We suggest that NLMS cells are therefore good candidates for mediating the perceptual recognition of prey and triggering the initiation of hunting responses.

### Tectal Assemblies Represent an Activity Motif Associated with Hunting Initiation

By imaging neural activity during behavior and comparing response and non-response trials, we could distinguish highly consistent visually evoked responses from activity specifically associated with the initiation of hunting.

We detected active tectal assemblies for ∼10% of convergence events. This rate of detection might reflect the probability of coincidence between our imaging plane and the locus of tectal activity, although we cannot exclude the possibility that only a subset of responses are associated with active assemblies. Notwithstanding, several lines of evidence support a close relationship between assembly activity and the execution of saccadic eye convergence. (1) Assemblies with similar characteristics were detected for stimulus-evoked and spontaneous convergences. (2) The laterality and anterior-posterior location of tectal assemblies correlated with oculomotor parameters of convergent saccades. (3) We occasionally observed repeated assembly activation when the animal performed very similar saccadic responses toward the same visual stimulus, and we were imaging the same focal plane (Figure S6). (4) Our false discovery rate analysis indicated that assemblies are unlikely to be a result of background spontaneous activity.

Tectal assemblies were active immediately prior to convergent saccades (pre-conv), and in many cases population activity began more than one second before the behavior. We suggest that coordinated burst firing of these assemblies provides the premotor signal that releases hunting responses. In support of this possibility, tectal activity has a well-established role in controlling goal-directed behaviors and saccadic eye movements [45] and direct stimulation of the anterior-medial tectum in fish evokes eye convergence [46] and J-turns [22]. Projections from the OTc to the mesencephalic reticular formation (MRF) provide the efferent pathway by which tectal activity can control saccadic eye movements (reviewed in [28]). Notably, tectal loci project bilaterally to the MRF but form a greater number of synapses on the ipsilateral side [29]. This asymmetry might provide the anatomical basis for our finding that assemblies were located ipsilateral to the eye that produced the larger, faster nasal rotation.

How might the activation of tectal assemblies be controlled? Following our hypothesis that NLMS cells mediate prey recognition, we propose that they provide a key afferent input to premotor assemblies. Notably, assemblies themselves contained few, if any, NLMS cells, suggesting that perception of prey and the release of predatory responses are mediated by non-overlapping populations of tectal neurons. NLMS cells could directly or indirectly provide excitatory input onto one or more assembly neurons, with local recurrent connections within the assembly contributing to sustained and synchronized population activity. One speculative possibility is that activity in assembly networks “ramps up,” or accumulates, during target viewing and triggers a behavioral response at a certain activity level corresponding to a perceptual threshold. Such a mechanism would be reminiscent of activity in cortical area LIP, where neurons show ramping activity that is thought to reflect the temporal integration of evidence during perceptual decision making [47]. Inter-hemispheric communication (for instance, via the tectal commissures) is likely to contribute to assembly recruitment because activity sometimes began while the visual cue was in the ipsilateral visual hemifield (with respect to the assembly), which is predominantly represented in the contralateral tectum. In addition to NLMS cells, other afferent inputs might modulate assembly activity (and thus response probability) in accordance with changes in internal state relating to arousal and motivation.

A significant fraction of peri-conv assemblies showed activation concurrent with, or subsequent to, convergent saccades. Although we cannot exclude the possibility of eye-movement associated activity (visual or proprioceptive sensory feedback, which could be tested by paralyzing the eye muscles), this may represent a motor efference copy of the saccadic command, which could derive from the reciprocal connections between the OTc and MRF [29]. Efference copy signals are thought to mediate saccadic suppression (a reduction in visual acuity during saccadic eye movements), allowing retinal movement signals due to external stimuli to be distinguished from reafferent signals generated during gaze shifts [48]. Such a mechanism could contribute to accurate perception of prey during hunting by suppressing self-generated motion signals during rapid eye and body movements. In support of this, zebrafish appear to show reduced sensitivity to visual stimuli during swim bouts [24].

### Conclusions

By combining functional calcium imaging with tethered virtual hunting behavior, we have functionally identified neuronal populations in the OTc that are likely key components in the sensorimotor transformations underlying a specific visually guided behavior. Our working model of the neural circuit for the initiation of hunting presents several testable hypotheses that could form the basis for future studies. In particular, determining how NLMS responses are generated and how NLMS neurons interface with premotor assemblies will be exciting challenges. High-speed volumetric imaging [23] presents the possibility to extend the analysis of neural activity from the OTc to the entire larval zebrafish brain and identify other regions that interact with and modulate core sensorimotor pathways. In addition, virtual reality hunting assays will allow circuit dynamics to be monitored during subsequent stages of hunting routines when larvae iteratively select goal-directed motor outputs to track, approach, and capture their prey.

## Experimental Procedures

### Animals

Zebrafish (*Danio rerio*) larvae homozygous for both the Tg(elavl3:GCaMP5G)a4598 transgene [23] and *mitfa*^w2/w2^ skin-pigmentation mutation [49] were used for all experiments. Larvae were raised in fish facility water on a 14/10-hr light/dark cycle and fed *Paramecia* from 4 days postfertilization (dpf). They were tested at 5–7 dpf. Animal handling and experimental procedures were approved by the Harvard University Standing Committee on the Use of Animals in Research and Training.

### Virtual Hunting Assay

The hunting assay for tethered larval zebrafish was performed as described in [13] and the Supplemental Experimental Procedures. Visual stimuli consisted of moving spots that appeared at 100° to the left or right of the midline and then moved 200° right or left across the frontal region of visual space at constant speed along the surface of the curved screen. Bright spots were maximum contrast red stimuli (pixel value 255, Weber contrast, *C*_*w*_ ∼370). For presentation of negative-polarity dark spots, there was first an increase in background luminance (red pixel value 25) and 2 s later a dark spot was presented (pixel value 0, *C*_*w*_ = −0.97). Whole-field light flashes were 3 s in duration at pixel values 15 (dim) or 25 (bright). Horizontal eye position was extracted at 60 Hz, and convergent saccades were detected as nasal rotations of both eyes within 150 ms of one another [13]. At each focal plane, we presented five to eight repetitions of each of the 18 visual stimuli (16 moving spots and two whole-field light-flashes) in pseudo-random order, with one stimulus presentation per 32 s “epoch.”

### 2P Functional Imaging

2P calcium imaging was performed using a custom-built microscope that included a 20× numerical aperture (NA) 0.95 Olympus objective and a Ti:Sapphire ultra-fast laser (Spectra-Physics MaiTai) tuned to 920 nm, with average laser power at sample of 5–10 mW. Images (500 × 500 pixels, pixel pitch 374 or 575 nm) were acquired by frame scanning at 1.8 Hz and for each larva, 10–15 focal planes were imaged with a z-spacing of 2 or 4 μm. Image acquisition, eye tracking, and visual stimulus presentation were controlled using software written in LabView and MATLAB.

### Data Analysis

Data analysis was performed using scripts written in MATLAB as described in the Supplemental Experimental Procedures. For all statistical tests, two-tailed p values are reported.

## Author Contributions

I.H.B. performed the experiments and analyzed the data. I.H.B. and F.E. conceived the project, interpreted the results, and wrote the manuscript.

## Supplemental Information

Document S1. Supplemental Experimental Procedures, Figures S1–S7, and Table S1

Document S2. Article plus Supplemental Information
