# Supplementary material for: Visuomotor Transformations Underlying Hunting Behavior in Zebrafish
Source: Curr Biol. 2015 Mar 30;25(7):831–46. doi: 10.1016/j.cub.2015.01.042 (PMC4386024; doi:10.1016/j.cub.2015.01.042)
Supplement: Document S1. Supplemental Experimental Procedures, Figures S1–S7, and Table S1 [file mmc1.pdf]

Current Biology
Supplemental Information

Visuomotor Transformations Underlying Hunting Behavior in Zebrafish

Isaac H. Bianco and Florian Engert

FIGURE S1

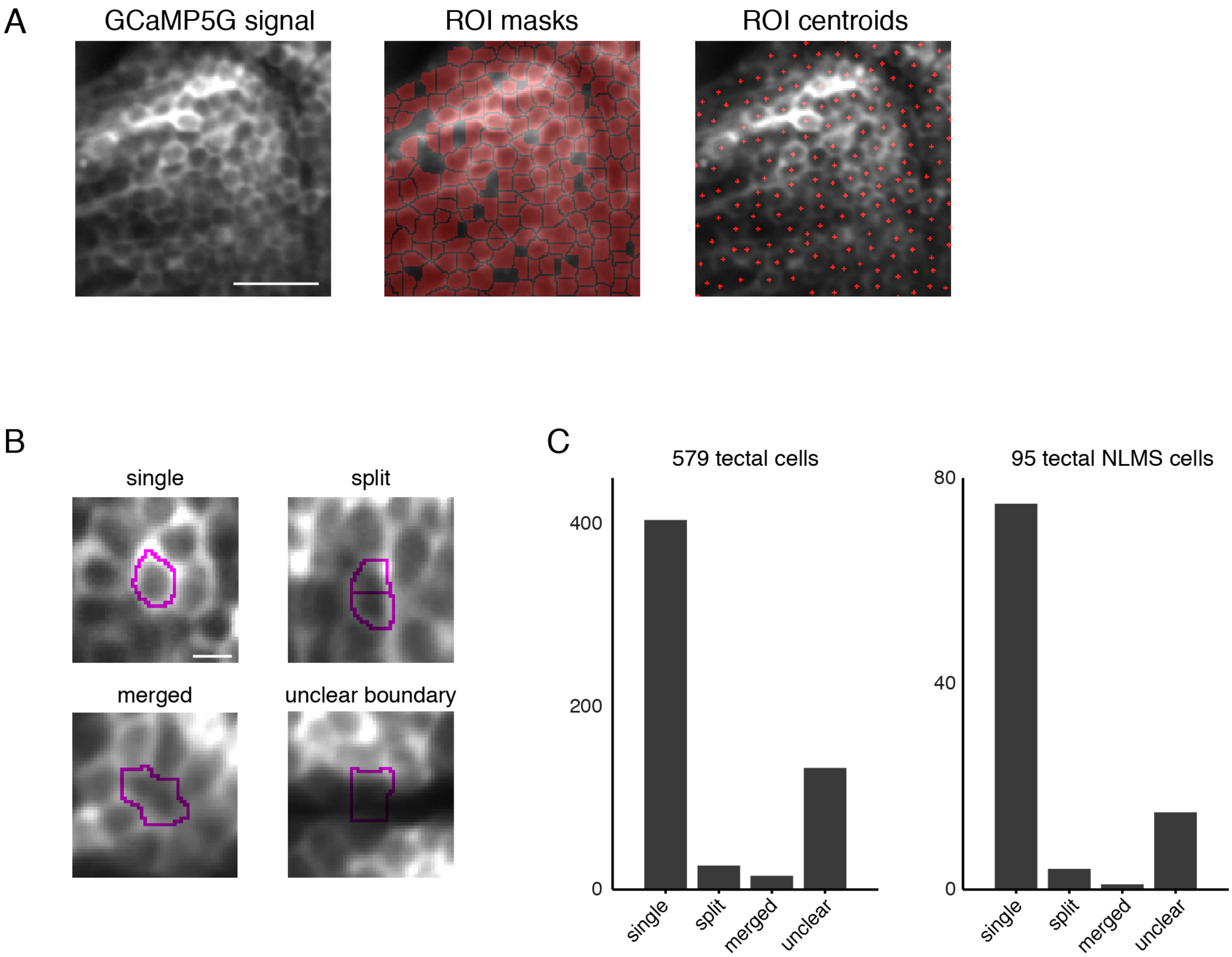
A
GCaMP5G signal
ROI masks
ROI centroids
B
single
split
merged
unclear boundary
C
579 tectal cells
95 tectal NLMS cells
400
200
0
80
40
single
split
merged
unclear

Figure S1 : Automatic identification of ROIs (related to Figure 3).

(A) Example of automated segmentation. Left panel shows ‘anatomical’ image obtained by taking the mean time-series projection of all motion-corrected images from a focal plane. Field-of-view shows a region of the anterior left OTc (SPV layer). Scale bar: 20 μm. Automatic segmentation was performed using a watershed-based algorithm [S1]. ROI masks (middle panel) or centroids (right panel) are shown overlaid on the anatomical image. The ’holes’ in the ROI masks correspond to ROIs that were rejected based on morphological criteria (see Experimental Procedures). ROIs corresponded well to individual somata and similar sized ROIs were detected in neuropil regions (not visible in this field-of-view). We refer to the former as cells, although it is possible these ROIs may include neurites of other neurons within the densely packed SPV of the OTc. For this fish, the average area of all ROIs was 15.9 μm^2 [11.5–20.7] (median, interquartile range). For ROIs assigned to cells body regions (i.e. excluding neuropil ROIs), average area was 12.7 μm^2 [6.4–19], equivalent to a circle of diameter 4.0 μm [2.9–4.9]. (B,C) We estimated error rates for the automated segmentation procedure by manually inspecting 579 randomly selected ROIs and classifying them as ‘single’, ‘split’, ‘merged’ or ‘unclear boundary’. (B) Examples of ROIs for each category. ‘Single’ are ROIs that clearly define a single cell soma. ‘Split’ ROIs correspond to part of a cell but where one or more additional ROIs are localized to other region(s) of the same cell. ‘Merged’ ROIs erroneously encompass more than one cell. ‘Unclear boundary’ ROIs did not show the typical ‘doughnut-shaped’ GCaMP signal localized to the cytoplasm/membrane surrounding a dark nucleus. These sometimes occurred at tissue boundaries (e.g. the midline as shown here) or corresponded to neurites. (C) Numbers of ROIs assigned to each category. Left panel: complete set of 579 ROIs. Right panel: 95 tectal NLMS cells, which were part of the random sample. Error rates for NLMS cells are comparable to the total sample.

FIGURE S2

A

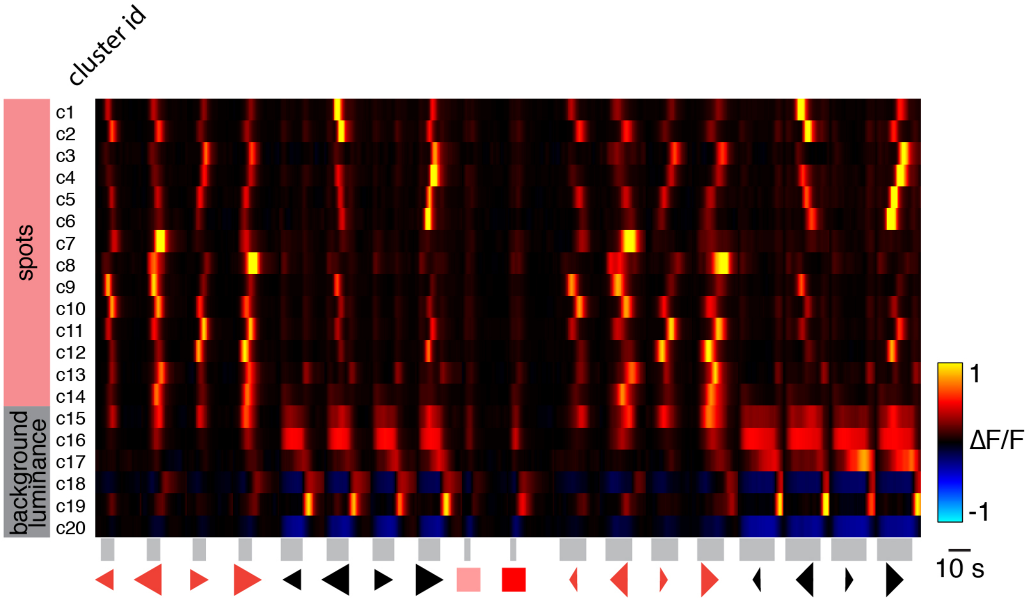

cluster id
spots
background luminance
c1
c2
c3
c4
c5
c6
c7
c8
c9
c10
c11
c12
c13
c14
c15
c16
c17
c18
c19
c20
1
ΔF/F
-1
10 s

B

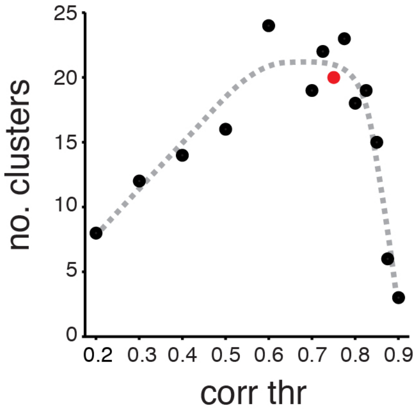

no. clusters
25
20
15
10
5
0
0.2 0.3 0.4 0.5 0.6 0.7 0.8 0.9
corr thr

Figure S2 : Additional cluster details (related to Figure 3).

(A) Heat-map representation of centroids of the 20 clusters of visually responsive neurons shown in Figure 3B. Centroids are shown as mean $\Delta F/F$ of all cells within each cluster. Gray bars and symbols indicate visual stimulus presentation periods. (B) Number of clusters identified by our clustering procedure as a function of correlation threshold. We chose a correlation threshold of 0.75, which produced 20 clusters (red datapoint, see also Experimental Procedures).

FIGURE S3

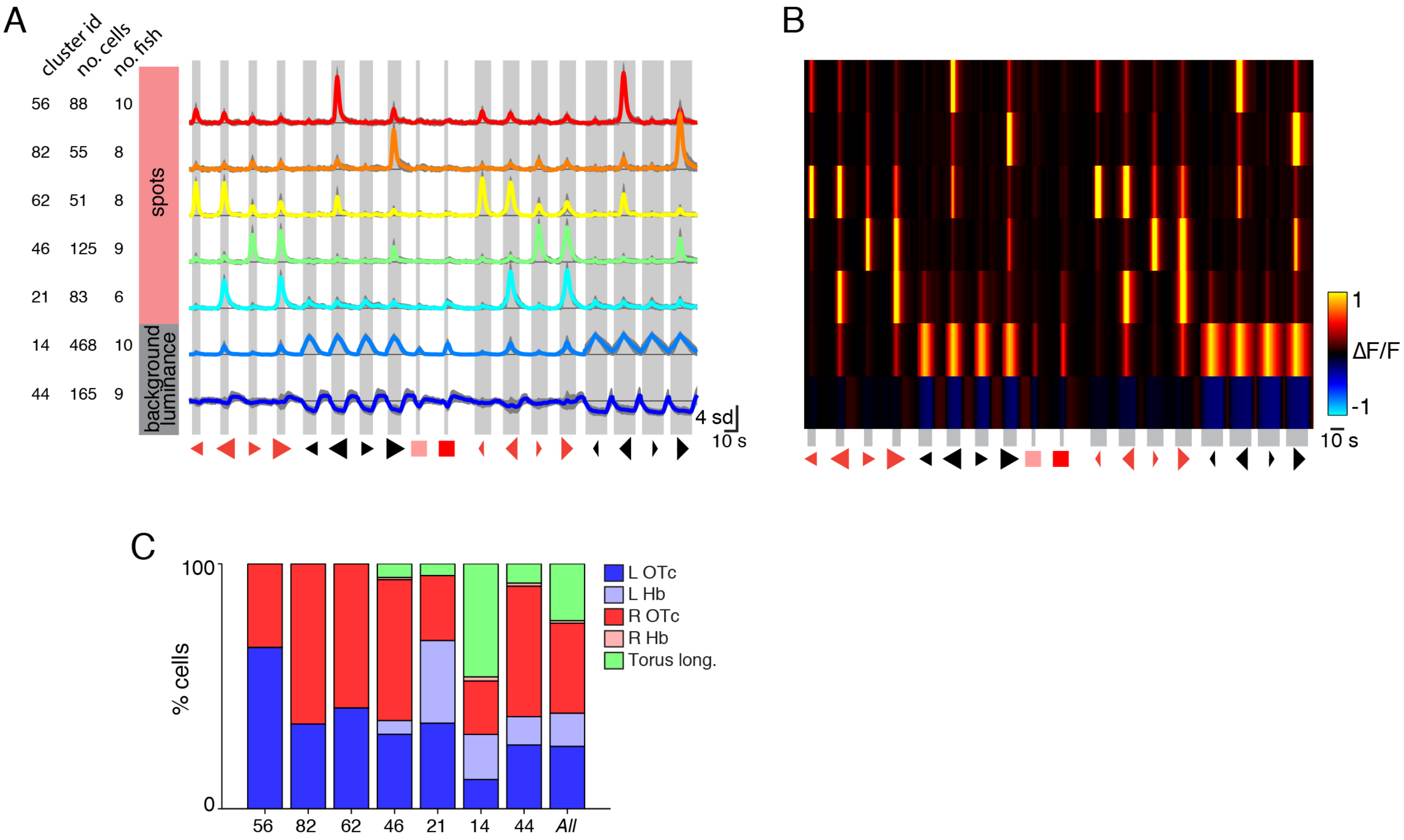
A
cluster id
no. cells
no. fish
56 88 10
82 55 8
62 51 8
46 125 9
21 83 6
14 468 10
44 165 9
spots
background luminance
4 sd
10 s
B
1
ΔF/F
-1
10 s
C
100
0
% cells
56
82
62
46
21
14
44
All
L OTc
L Hb
R OTc
R Hb
Torus long.

Figure S3 : Clustering using Gaussian Mixture Modeling (related to Figure 3).

(A,B) Neurons from 10 fish were clustered using a method based on Gaussian mixture modeling. The centroids of the 7 clusters that were identified are shown in (A) and alternatively as a heat-map representation in (B). Shading in (A) represents standard deviation across cells. Note that prior to clustering, the peak responses of all cells were aligned for each stimulus, such that they appear to respond maximally when the visual cues are at 0°. Consequently, cells are not segregated based on differential timing of their fluorescence responses and so fewer clusters are produced as compared to Figure 3B. However, clusters with comparable visual feature tuning are detected. Shaded bars indicate visual stimulus presentation. (C) Anatomical locations of the cells associated with each cluster.

FIGURE S4

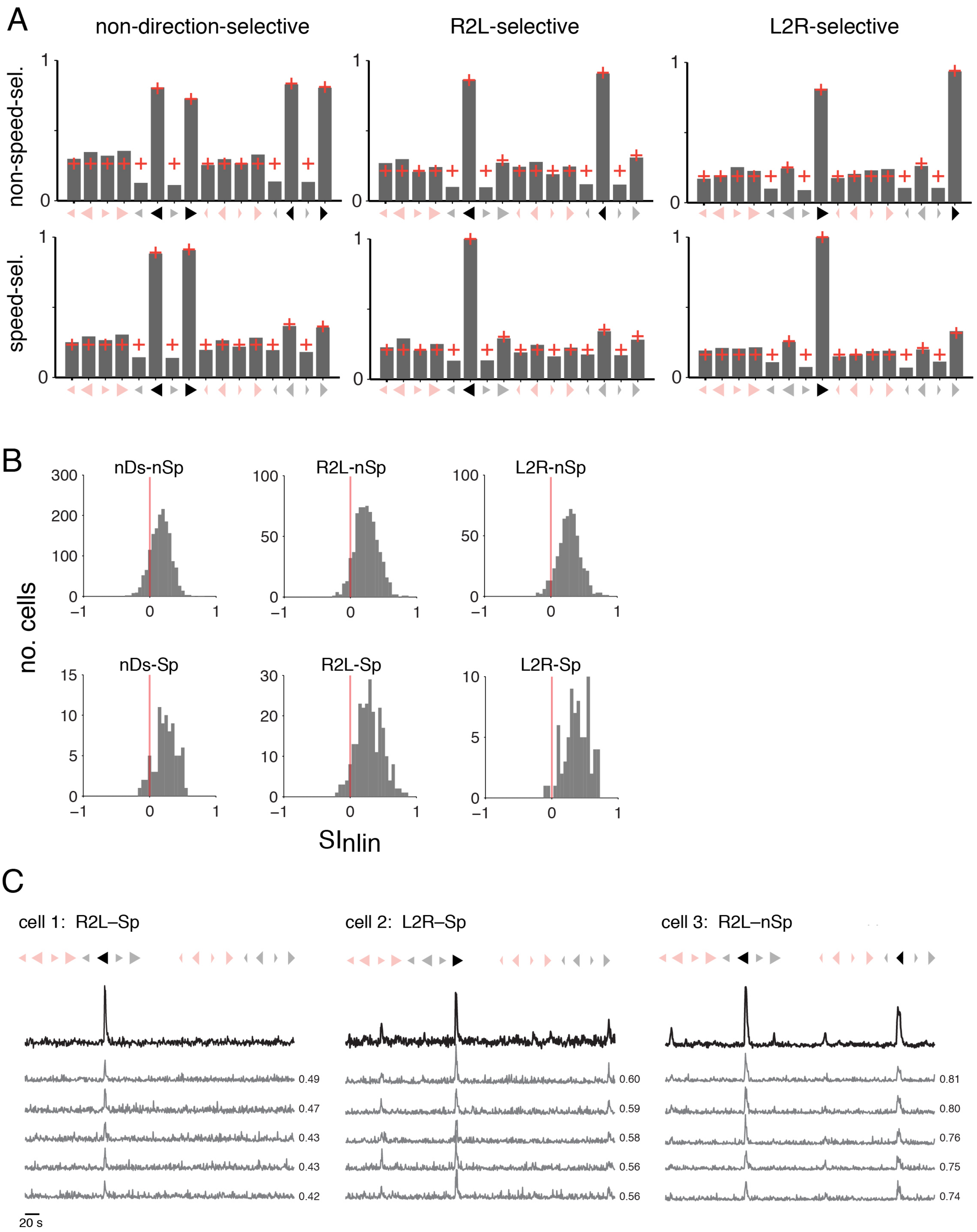
A
non-direction-selective
R2L-selective
L2R-selective
non-speed-sel.
speed-sel.
B
nDs-nSp
R2L-nSp
L2R-nSp
nDs-Sp
R2L-Sp
L2R-Sp
no. cells
SInlin
C
cell 1: R2L–Sp
cell 2: L2R–Sp
cell 3: R2L–nSp
0.49
0.47
0.43
0.43
0.42
0.60
0.59
0.58
0.56
0.56
0.81
0.80
0.76
0.75
0.74
20 s

Figure S4 : Non-linear model fits and single NLMS voxels (related to Figure 5).

(A) Comparison of observed stimulus responses to those predicted by the non-linear model in Figure 5B. Gray bars indicate mean normalized responses and red crosses indicate mean normalized responses predicted by the model. Model predictions were computed for each ROI using the fitted model coefficients. To facilitate comparison of tuning profiles between ROIs, responses were normalized to the maximal stimulus response. (B) Non-linear selectivity index (SI_{nlin}) for ROIs associated with each regressor. (C) Responses of single voxels from NLMS ROIs. Three examples are shown of NLMS ROIs corresponding to neuronal somata in the optic tectum. Schematics indicate the regressor with which each ROI is associated. Top traces show the visual response vectors (VRVs) for the ROI (average of all component pixels, black) and lower traces show VRVs for single voxels belonging to the ROI (gray). Numbers on the right indicate the Pearson's correlation coefficient between the voxel VRV and ROI VRV. Although traces for single voxels show reduced signal-to-noise, they clearly display similar stimulus selectivity to the parent ROI. All traces shown as unfiltered raw pixel values (arbitrary units).

FIGURE S5

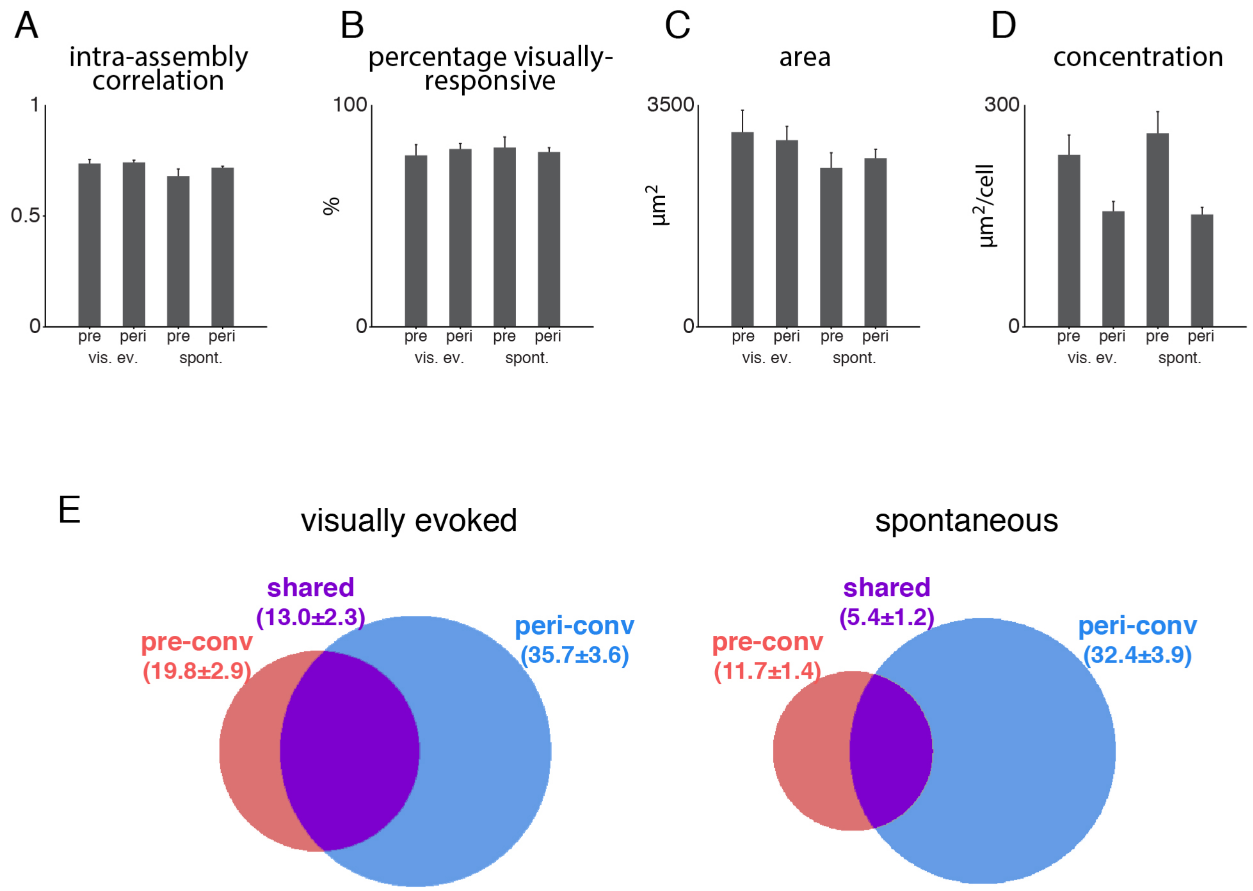

A
intra-assembly correlation
1
0.5
0
pre
peri
pre
peri
vis. ev.
spont.
B
percentage visually-responsive
100
%
0
pre
peri
pre
peri
vis. ev.
spont.
C
area
3500
μm2
0
pre
peri
pre
peri
vis. ev.
spont.
D
concentration
300
μm2/cell
0
pre
peri
pre
peri
vis. ev.
spont.
E
visually evoked
shared
(13.0±2.3)
pre-conv
(19.8±2.9)
peri-conv
(35.7±3.6)
spontaneous
shared
(5.4±1.2)
pre-conv
(11.7±1.4)
peri-conv
(32.4±3.9)

Figure S5 : Additional characteristics of tectal cell assemblies (related to Figure 6).

(A) Intra-assembly correlation: Mean correlation of activity of each cell in an assembly with the mean activity of the assembly. (B) Percentage visually responsive cells: Percentage of cells in the assembly classified as responsive to at least one of the 18 visual stimuli. (C) Area: Area of ellipse fit to the spatial distribution of response-modulated cells. (D) Concentration: Area of ellipse divided by number of cells enclosed by ellipse (number of cells in assembly). (E) Venn diagram showing overlap between pre-conv and peri-conv assemblies. Numbers in parentheses indicate cells belonging to each type of assembly and shared by both assemblies. Data presented as mean $\pm$ sem. Abbreviations: pre, pre-conv assembly; peri, peri-conv assembly; vis. ev., visually evoked convergence; spont., spontaneous convergence.

FIGURE S6

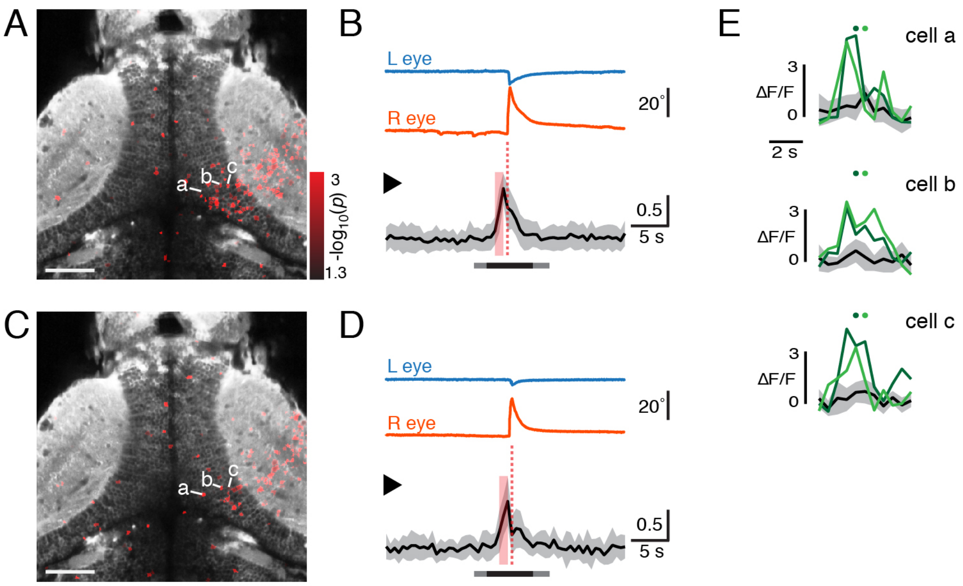
A
a
b
c
3
-log10(p)
1.3
B
L eye
R eye
20°
0.5
5 s
C
D
E
cell a
3
ΔF/F
0
2 s
cell b
cell c

Figure S6 : Repeated activation of a tectal assembly (related to Figure 6).

Example of two pre-conv assemblies that were detected in overlapping anatomical locations in the right OTc and associated with highly similar behavioral responses. (A,B) First pre-conv assembly (same example as Figure 6G,H). (A) Response-modulated ROIs (red) overlaid on an anatomical projection image (gray). Scale bar: 50 μm. (B) Top: Eye position traces. Bottom: Normalized activity of all cells within the assembly (mean $\pm$ sd). Notice that the right eye shows the greater nasal rotation and the assembly is located in the right OTc. (C,D) Second pre-conv assembly that was active approximately 1.5 min later in association with another prey-catching response. Notice that the larva performed a similar saccadic response to the same type of visual stimulus (large, dark, fast, left–right moving spot) and the assemblies occupy overlapping anatomical locations in the right OTc. (E) Three examples of response-modulated cells that were common to both assemblies (locations marked in (A,C)). Responses for the first and second convergence events are shown in dark and light green respectively. Dots above the traces indicate the imaging frame during which the convergent saccade occurred. Activity in non-response trials, for the same visual stimulus, is shown in gray (mean $\pm$ sd).

FIGURE S7

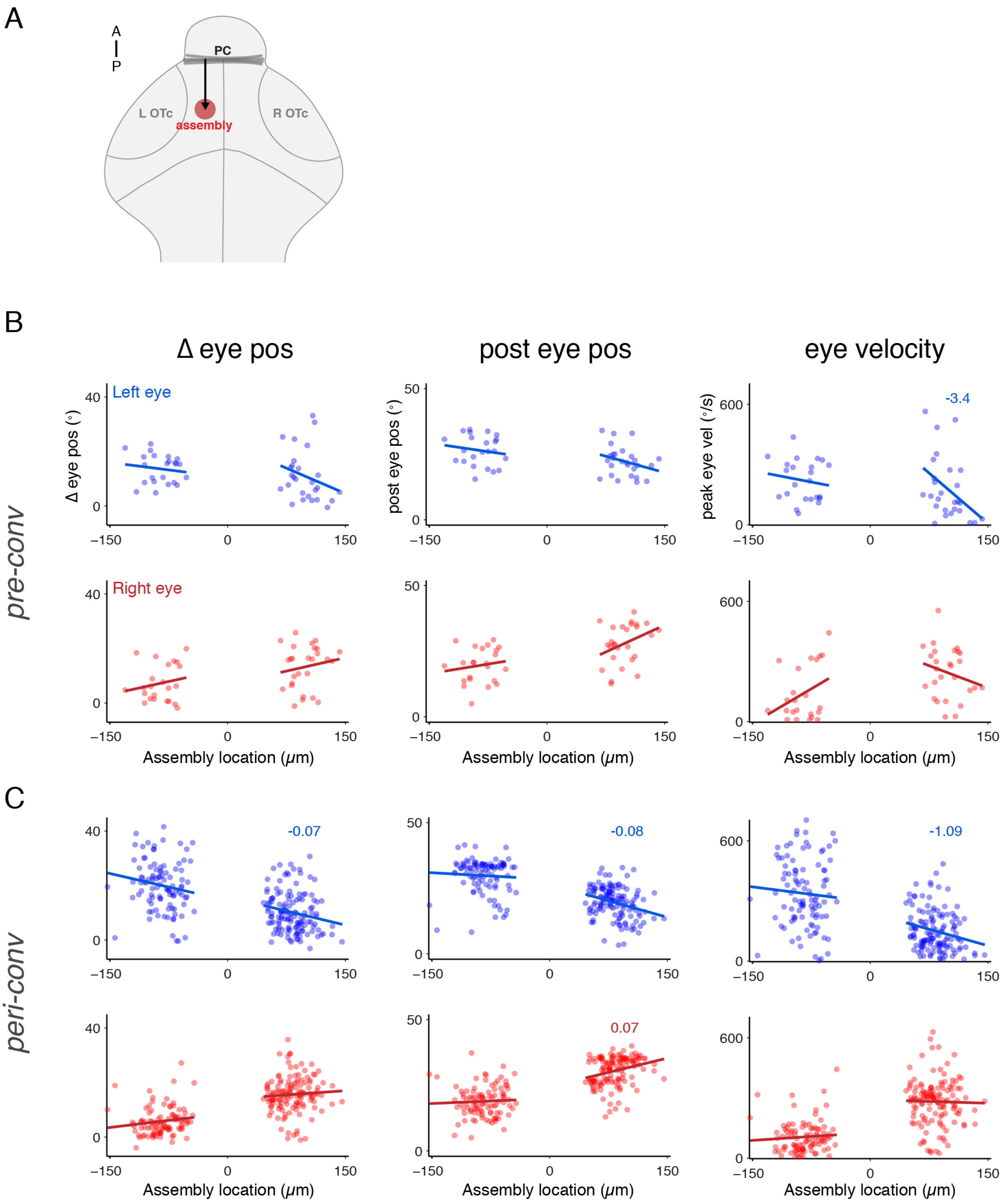
A
A
P
PC
L OTc
R OTc
assembly
B
Δ eye pos
post eye pos
eye velocity
Left eye
Right eye
Δ eye pos (°)
post eye pos (°)
peak eye vel (°/s)
-3.4
pre-conv
Assembly location (μm)
C
-0.07
-0.08
-1.09
0.07
peri-conv

Figure S7 : Comparison of oculomotor responses to anatomical location of tectal assemblies (related to Figure 6).

(A) Schematic illustrating how assembly location was measured. The distance from the center of the posterior commissure (PC) to the center-of-mass of the assembly was measured parallel to the anterior-posterior axis. The locations of left tectal assemblies are given as negative values (more negative values correspond to more caudal locations) whereas locations in the right tectum are given as positive values (greater values represent more caudal locations). (B) Comparison of oculomotor responses to locations of pre-conv assemblies. Upper row represents left eye, lower row is right eye. Left panels show change in eye position during convergent saccade, middle panels show eye position immediately following the convergent saccade and right panels show peak eye velocity during the saccade. For all parameters, positive values correspond to nasalward position or velocity. Lines indicate independent straight line fits for left or right tectal assemblies. Where the 95% confidence interval for the gradient of the line did not overlap with zero, the fit value of the gradient is shown. (C) Data for peri-conv assemblies, as per (B).

Table S1 : Details of visually responsive clusters

Cluster id	No. cells	No. fish	r mean ± sd	Type	SI Direction mean ± sd	SI Size mean ± sd	SI Speed mean ± sd	SI Polarity mean ± sd	Notes
1	216	11	0.78 ± 0.06	Spot	-0.27 ± 0.21	0.47 ± 0.12	-0.03 ± 0.12	0.23 ± 0.18	Tail–nose selective, L OTc
2	267	13	0.76 ± 0.07	Spot	-0.32 ± 0.26	0.38 ± 0.13	0.01 ± 0.12	0.05 ± 0.21	Nose–tail selective, R OTc
3	31	9	0.81 ± 0.04	Spot	0.48 ± 0.11	0.30 ± 0.11	-0.05 ± 0.07	-0.11 ± 0.13	Nose–tail selective, L OTc
4	135	9	0.77 ± 0.06	Spot	0.31 ± 0.21	0.45 ± 0.12	-0.04 ± 0.16	0.23 ± 0.16	Nose–tail selective, L OTc
5	74	8	0.79 ± 0.03	Spot	0.18 ± 0.13	0.36 ± 0.11	-0.12 ± 0.13	0.06 ± 0.15	Tail–nose selective, R OTc
6	71	9	0.79 ± 0.06	Spot	0.31 ± 0.19	0.55 ± 0.12	-0.11 ± 0.11	0.36 ± 0.16	Tail–nose selective, R OTc
7	108	11	0.77 ± 0.05	Spot	-0.37 ± 0.14	0.43 ± 0.13	0.01 ± 0.17	-0.49 ± 0.13	Nose–tail selective, R OTc
8	122	12	0.77 ± 0.06	Spot	0.24 ± 0.20	0.45 ± 0.12	-0.03 ± 0.14	-0.46 ± 0.16	Nose–tail selective, L OTc
9	215	12	0.75 ± 0.09	Spot	-0.45 ± 0.18	0.26 ± 0.19	-0.03 ± 0.15	-0.38 ± 0.18	Tail–nose selective, L OTc
10	429	13	0.76 ± 0.07	Spot	-0.13 ± 0.22	0.15 ± 0.14	0.00 ± 0.12	-0.47 ± 0.16	Nose–tail selective, R OTc
11	274	12	0.76 ± 0.06	Spot	0.29 ± 0.24	0.20 ± 0.16	-0.03 ± 0.09	-0.35 ± 0.21	Nose–tail selective, L OTc
12	244	13	0.78 ± 0.07	Spot	0.48 ± 0.18	0.26 ± 0.14	-0.05 ± 0.11	-0.32 ± 0.19	Tail–nose selective, R OTc
13	40	6	0.77 ± 0.06	Spot	0.03 ± 0.12	0.38 ± 0.14	-0.12 ± 0.11	-0.56 ± 0.12	Non-direction selective
14	701	14	0.71 ± 0.08	Spot	0.03 ± 0.17	0.45 ± 0.18	-0.06 ± 0.12	-0.48 ± 0.19	Non-direction selective
15	45	6	0.77 ± 0.05	Lum					Responsive to all stimuli
16	1839	14	0.77 ± 0.11	Lum					Positive modulation to luminance increase
17	17	7	0.81 ± 0.04	Lum					Slow positive modulation to luminance increase
18	126	10	0.82 ± 0.07	Lum					Negative modulation to luminance increase & positive modulation to luminance decrease
19	52	9	0.79 ± 0.06	Lum					Positive modulation to luminance decrease
20	86	9	0.78 ± 0.06	Lum					Negative modulation to luminance increase

Notes:

1. r : Pearson's correlation coefficient between each cell's visual response vector and the cluster centroid

2. Selectivity Indices (SI):
   Direction : Rightwards, positive; Leftwards, negative
   Size : Large, positive; Small, negative.
   Speed : Fast, positive; Slow, negative.
   Polarity : Dark, positive; Bright, negative.

3. Abbreviations:
   Spot : moving spot responsive
   Lum : background luminance responsive
   L : left
   R : right
   OTc : optic tectum

Supplemental Experimental Procedures

Virtual hunting assay

The virtual hunting assay was similar to that described in [S2]. Larval zebrafish were mounted in 2% low-melting temperature agarose (Invitrogen), dissolved in fish-facility water, in a 35 mm petri-dish lid. Once the agarose had gelled, an opthalmic scalpel was used to carefully remove the agarose anterior to the otic vesicle and caudal to the swim bladder. Consequently the animal was held around the head and body but was able to freely move its eyes and tail. A strip of diffusive filter (3026, Rosco Inc, CA) was fixed to the outside wall of the chamber and served as a screen. The mid-point between the fish's eyes was 7 mm from the screen. Visual stimuli were designed in Matlab (Mathworks, MA) using the Psychophysics toolbox [S3] and back-projected onto the screen using a laser pico-projector (SHOWWX, Microvision). A colored Wratten filter (no. 25, Kodak) was placed in front of the projector to minimize interference with GCaMP imaging; we detected no bleed-through of the visual stimulus to our imaging data. To monitor eye movements, a 720 nm LED was used to illuminate the larva from below through a diffuser and the eyes were imaged at 60 Hz using a charge-coupled device camera (Guppy F-033, Allied Vision Technology). Eye-tracking was performed using custom scripts written in LabView (National Instruments). Horizontal eye position was defined as the angle between the long axis of an ellipse fit to the eye and a line parallel to the midline of the head and convergent saccades were detected using custom scripts written in Matlab.

2P functional imaging

The resolution of the microscope was estimated as 370 nm laterally and 1.44 μm axially (FWHM) as per [S4].

Data analysis

We used logistic regression to model response rate as a function of four binary-coded stimulus features. To identify the model that best described the data, we used stepwise regression, which iteratively adds or removes model terms to optimize the model fit. Depending on starting conditions, the stepwise regression algorithm converged on one of the following two models:

Model A:

$$ln\left(\frac{R}{1-R}\right) = \beta_0 + \beta_1[Sz] + \beta_2[Pol] + \beta_3[Sp \cdot Sz]$$

Model B:

$$ln\left(\frac{R}{1-R}\right) = \beta_0 + \beta_1[Sz] + \beta_2[Pol] + \beta_3[Sp \cdot Sz \cdot Pol]$$

where R is response rate, $R/(1-R)$ is response odds and Dir, Sz, Sp and Pol are binary variables describing the features direction, size, speed and contrast-polarity with the coding scheme summarized in Figure 2E. As a principled means to compare these models we used cross-validation to evaluate model accuracy. We randomly segregated our dataset into two equal parts to produce a training and a test dataset. Both models were fit using the training dataset and the model predictions were then evaluated against the unseen test dataset by computing the proportion of the variance of the test dataset explained by the model (R^2). Model B had a slightly greater cross-validated $R^2 = 0.82$ compared to Model A, $R^2 = 0.79$. In Figure 2 we show the response rates predicted by Model B and the estimated model coefficients.

To correct for slow specimen drift and small movements of the animal, individual imaging frames were aligned to the mean image for that z-plane with sub-pixel precision [S5]. The typical displacement vector required to align an individual frame was less than one pixel-spacing. However, frames that required more than 3.7 μm correction (*ie.* approaching a cell body diameter, $\sim$ 5 μm) were replaced with the mean image for the epoch and epochs were discarded if they included more than one such frame. Larvae occasionally perform strong motor behaviors ('struggles') and these criteria eliminated such epochs from our analysis. Motion-corrected images were used for all subsequent analysis.

Neurons in GCaMP larvae appear as bright rings of cytoplasm enclosing darker central nuclei ('doughnuts'). Images were segmented to define ROIs corresponding to individual neurons using a watershed-based algorithm [S1]. A high-contrast image was obtained by taking the mean of the (motion-corrected) image time-series. This 'anatomical' image was gaussian-smoothed followed by local histogram stretching and finally a watershed algorithm was used to identify putative somata. ROIs were removed if they had very small (< 5 μm^2) or large (> 100 μm^2) areas. ROIs were manually discarded if they were located in regions of auto-fluorescent skin or outside the brain. Remaining ROIs, corresponding to somata or neuropil regions, were then manually assigned to anatomical structures. By inspecting a random sample of automatically segmented ROIs, we found that error rates (*eg.* merging or splitting of individual somata) were low (Figure S1). Furthermore, we estimated contamination of the fluorescence signals of ROIs by adjacent cells/neuropil as follows: For single voxels, we computed the correlation of that voxel's complete fluorescence time-series with that of the parent ROI versus the highest correlation with any directly adjacent ROI (neighboring cells/neuropil). The percentage of voxels that were better correlated with an adjacent ROI was less than 1% (0.38%, $n = 368,585$ pixels). Inspection of maps of these correlation coefficients suggested this small number of errors most frequently arose when a cell had been erroneously subdivided into more than one ROI.

In our experiments fish were free viewing. To estimate spatial receptive fields (RFs) we corrected for changes in eye position by assessing the difference in the mean eye position mea-

sured during each imaging frame from the median eye position recorded over the course of the experiment. The angular deviation was used to correct the angular position of the moving spot for that frame, allowing us to estimate activity in eye-fixed coordinates. This procedure assumes that eye movements are not associated with spatiotemporal dynamics in RF structure.

The time-varying fluorescence signal $F(t)$ for each cell was extracted by computing the mean value of all pixels comprising an ROI at each time-point (imaging frame). The proportional change in fluorescence ($\Delta F/F$) at time t was calculated as

$$\Delta F/F = \frac{F(t) - F_0}{F_0}$$

where F_0 is a reference fluorescence value, taken as the mean of $F(t)$ during 10 frames prior to visual stimulus presentation. To compute visual response vectors, the mean $\Delta F/F$ time-series was computed across the set of repetitions for each visual stimulus. Mean profiles were then smoothed with a 3-frame box-car filter and the first 20 frames (corresponding to baseline period with no stimulus presentation) were removed. Average responses were then concatenated to produce the visual response vector (VRV) for the cell. VRVs comprised 684 time-points, representing the average response of the ROI to the 18 stimuli in succession.

As a preliminary means to classify ROIs as visually responsive or not, we used t-tests to compare the value of the VRV during stimulus presentation versus during baseline (10 frames prior to stimulus onset). Note that this analysis was performed using VRVs from which we did not remove the baseline frames. For each ROI we performed two t-tests for each of the 18 visual stimuli. The first compared VRV values during baseline versus during stimulus presentation and the second compared baseline to a 2.75 s period immediately following stimulus presentation (as some cells responded to the disappearance of the stimulus). ROIs were classified as visually responsive if any of the 36 tests produced a significant result, at a p-value threshold of 0.02.

We clustered cells from 14 fish based on their VRVs using a method similar to [S6]. The Pearson's correlation between all pairs of VRVs was computed. The two cells with the highest correlation coefficient were then joined into a cluster and the process was repeated by progressively joining cells and clusters until no pairwise correlation exceeded threshold. The centroid of a cluster was taken as the mean of the VRVs within the cluster. We discarded clusters if they did not contain at least 5 cells from 5 different fish as we considered this to be a reasonable standard for identifying reproducible visual response properties. In this procedure, correlation threshold is a free parameter. We found that the number of clusters identified as a function of correlation threshold followed an approximately bell-shaped curve, where few clusters were identified at low threshold (large, poorly tuned clusters) or high threshold (few cells with sufficiently similar VRVs) (Figure S2B). However, there was a range ($\sim$ 0.6–0.8) where a similar number of clusters were

identified, suggesting a consistent partitioning of the data. We selected a correlation threshold of 0.75, which fell within this range.

In Figure S3 we tested an alternative clustering procedure, based on Gaussian Mixture Modeling. First, for each visual stimulus, the peak response of each cell was aligned such that all cells appear to respond at the same time. Principal components analysis was applied to the dataset and the data were projected onto the first 18 principal components. Next, cells were clustered by fitting a Gaussian Mixture Model with 100 components and requiring that for a cell to be assigned to a cluster, the posterior probability must exceed 99%. We discarded clusters containing less than 25 cells, from 5 different fish. Very similar clusters were identified even if we varied the number of components in the model, but clusters became poorly tuned if we relaxed the posterior probability requirement and/or the minimum cell content requirement.

Selectivity Indices for each of the four stimulus features were calculated as:

$$SI_{feature} = \frac{R_1 - R_0}{R_1 + R_0}$$

where R_1 is the sum of the maximum responses (maximum $\Delta F/F$ during spot presentation) for stimuli with feature coded 1 and R_0 is the sum of the maximum responses to the moving spot stimuli with feature coded 0 (refer to binary coding scheme in Figure 2E). Thus, a cell that responds only to leftwards moving spots (leftwards motion is coded 0) will have $SI_{dir} = -1$.

To identify mixed-selectivity neurons, we first computed a vector for each cell describing the peak mean response to each of the 16 moving spot stimuli. We then evaluated the Pearson's correlation coefficient, r, between each neuron's vector and six 16-dimensional binary 'regressors'. The values of the regressors are indicated in Figure 5A. Cells were associated with the regressor that produced the highest r and where $r > 0.75$. Generalized linear regression was used to model the responses of each cell that was associated with a regressor as a function of the four binary stimulus features. The maximum responses to all the individual presentations of the 16 moving spot stimuli were computed (*ie.* 80–128 responses/cell), and this response vector (y) was used to fit the following two models.

Non-linear:

$$y = \beta_0 + \beta_1[Sz \cdot Pol] + \beta_2[Dir \cdot Sz \cdot Pol] + \beta_3[Sp \cdot Sz \cdot Pol] + \beta_4[Dir \cdot Sp \cdot Sz \cdot Pol]$$

Linear:

$$y = \beta_0 + \beta_1[Dir] + \beta_2[Sz] + \beta_3[Sp] + \beta_4[Pol]$$

As a principled means to compare which model provided a more accurate description of the cell's activity we computed a cross-validated R^2 using the 'leave-one-out' method. For each

iteration, a single data point was excluded and the remaining 'training dataset' was used to fit the model parameters. The resulting model was then used to predict the missing datapoint. This process was repeated to predict the full set of responses, which were then compared to the data by computing the proportion of variance explained by the model predictions (cross-validated R^2). We compared the cross-validated R^2 obtained with the linear and non-linear models by computing:

$$SI_{nlin} = \frac{R^2_{nlin} - R^2_{lin}}{R^2_{nlin} + R^2_{lin}}$$

where positive values would indicate the non-linear model provided a more accurate description of the data.

To detect neurons that showed activity modulation associated with hunting responses, we compared activity between response trials and non-response trials during two convergence-triggered 'windows'. The *pre-conv* window was from -3 frames to -1 frames relative to the 'convergence frame' (frame 0, during which the convergent saccade occurred). The *peri-conv* window was from -2 frames to +2 frames relative to the convergence frame. For each cell, we used t-tests to compare $\Delta F/F$ during these windows in response versus non-response trials. We also estimated the maximum signal-to-noise ratio (SNR) during each window in response trials, defined as:

$$SNR = \frac{F(t) - F_0}{\sigma}$$

where σ is the standard deviation of F during the 10 frames prior to stimulus presentation in all epochs corresponding to that visual stimulus. For each window type (pre-conv and peri-conv) we considered response-modulated cells as those with $p < 0.05$ and $SNR > 3$. Note that we applied the SNR criterion to improve the detection of response-modulated cells only after we had determined that we did not detect cells showing negative modulation. To detect tectal assemblies, we fit an ellipse to the spatial distribution of response-modulated cells. The ellipse was centered at the center-of-mass of the cell population with long and short axes equal to the eigenvectors of the covariance matrix of the x, y positions of all modulated cells. Cells were considered part of the assembly if their centroid fell inside this ellipse. Note that this conservative method will often underestimate the number of cells within an assembly. We defined assemblies as having a minimum of 6 cells, with a minimum density of 533 μm^2/cell (ratio of area of ellipse to number of cells) and where at least 70% of cells were located in the same tectal hemisphere. These criteria were established empirically by inspection of maps of response-modulated cells and prevented the detection of assemblies comprising widely scattered cells that were clearly distinct from the coherent clusters of active neurons exemplified in Figure 6. Only ROIs within the tectal SPV were included in our analysis. We estimated onset of assembly activity by finding the first time-point where the mean calcium signal ($\Delta F/F$) of cells within the assembly (population

response) exceeded a threshold (Figure 6P). Threshold was determined by first defining baseline frames as those outside of a 9.35 s window centered on the saccade. The mean and standard deviation of the population response during baseline was computed and threshold was set at mean+4·sd. To estimate false discovery rate, we constructed artificial response and non-response epochs by circularly permuting the activity time-courses of all neurons in a *z*-plane after removing periods corresponding to convergent saccades (-3 to +3 frames with respect to convergence frame) and visual stimulus presentations. The same permutation was applied to all cells to preserve correlations between neurons as well as the higher order statistics of a single cell's activity. We then evaluated the number and characteristics of assemblies using identical criteria to those applied to the original data. We report false discovery rate as the ratio of the mean number of assemblies detected from 5 permutations to the number detected in the original data.

For all statistical tests, two-tailed *p*-values are reported.

Supplemental References

S1. Panier, T., Romano, S. A., Olive, R., Pietri, T., Sumbre, G., Candelier, R., and Debrégeas, G. (2013). Fast functional imaging of multiple brain regions in intact zebrafish larvae using selective plane illumination microscopy. Front Neural Circuits *7*, 65.

S2. Bianco, I. H., Kampff, A. R., and Engert, F. (2011). Prey capture behavior evoked by simple visual stimuli in larval zebrafish. Front Syst Neurosci *5*, 101.

S3. Brainard, D. H. (1997). The Psychophysics Toolbox. Spat Vis *10*, 433–6.

S4. Zipfel, W. R., Williams, R. M., and Webb, W. W. (2003). Nonlinear magic: multiphoton microscopy in the biosciences. Nat Biotechnol *21*, 1369–77.

S5. Nestares, O. and Heeger, D. J. (2000). Robust multiresolution alignment of MRI brain volumes. Magn Reson Med *43*, 705–15.

S6. Niell, C. M. and Smith, S. J. (2005). Functional imaging reveals rapid development of visual response properties in the zebrafish tectum. Neuron *45*, 941–51.
