# Supplementary material for: Visuomotor Transformations Underlying Hunting Behavior in Zebrafish
Source: Curr Biol. 2015 Mar 30;25(7):831–46. doi: 10.1016/j.cub.2015.01.042 (PMC4386024; doi:10.1016/j.cub.2015.01.042)
Supplement: Document S2. Article plus Supplemental Information [file mmc2.pdf]

Article

Current Biology

Visuomotor Transformations Underlying Hunting Behavior in Zebrafish

Highlights

- Zebrafish hunting responses are triggered by conjunctions of visual features
- Tectal neurons show non-linear mixed selectivity for prey-like visual stimuli
- Tectal assemblies show premotor activity specifically preceding hunting responses

Authors

Isaac H. Bianco, Florian Engert

Correspondence

i.bianco@ucl.ac.uk

In Brief

By combining a virtual hunting assay with functional calcium imaging, Bianco and Engert identify highly selective neurons in the zebrafish optic tectum that are likely to underlie visual prey recognition. The recruitment of tectal assemblies appears to link perception to action by providing the premotor commands that release hunting responses.

Bianco & Engert, 2015, Current Biology *25*, 831–846
March 30, 2015 ©2015 The Authors
http://dx.doi.org/10.1016/j.cub.2015.01.042

Current Biology
Article

CellPress

Visuomotor Transformations Underlying Hunting Behavior in Zebrafish

Isaac H. Bianco[1,2,*] and Florian Engert[1]
[1]Department of Molecular and Cellular Biology and Center for Brain Science, Harvard University, Cambridge, MA 02138, USA
[2]Present address: Department of Neuroscience, Physiology, and Pharmacology, University College London, London WC1E 6BT, UK
*Correspondence: i.bianco@ucl.ac.uk
http://dx.doi.org/10.1016/j.cub.2015.01.042
This is an open access article under the CC BY license (http://creativecommons.org/licenses/by/4.0/).

SUMMARY

Visuomotor circuits filter visual information and determine whether or not to engage downstream motor modules to produce behavioral outputs. However, the circuit mechanisms that mediate and link perception of salient stimuli to execution of an adaptive response are poorly understood. We combined a virtual hunting assay for tethered larval zebrafish with two-photon functional calcium imaging to simultaneously monitor neuronal activity in the optic tectum during naturalistic behavior. Hunting responses showed mixed selectivity for combinations of visual features, specifically stimulus size, speed, and contrast polarity. We identified a subset of tectal neurons with similar highly selective tuning, which show non-linear mixed selectivity for visual features and are likely to mediate the perceptual recognition of prey. By comparing neural dynamics in the optic tectum during response versus non-response trials, we discovered premotor population activity that specifically preceded initiation of hunting behavior and exhibited anatomical localization that correlated with motor variables. In summary, the optic tectum contains non-linear mixed selectivity neurons that are likely to mediate reliable detection of ethologically relevant sensory stimuli. Recruitment of small tectal assemblies appears to link perception to action by providing the premotor commands that release hunting responses. These findings allow us to propose a model circuit for the visuomotor transformations underlying a natural behavior.

INTRODUCTION

To generate visually guided behavior, the nervous system extracts task-relevant information from the retinal image to select and control an appropriate response. Over 50 years ago, neuroethologists introduced the idea that specific behaviors can be triggered by "key stimuli," delivered under appropriate conditions [1, 2]. In this context, individual neurons have been discovered in visual pathways that are proposed to function as "feature detectors." Such neurons are selective for specific spatiotemporal patterns within the visual scene and include neurons tuned to visual features that define key stimuli. Notably, stimulus-response pathways are subject to various modulating influences, and consequently "key stimuli" do not always trigger a response. Motivational state, arousal, attention, recent experience, and long-term memory can influence response probability, stimulus preference, and the choice of motor outputs (e.g., [3, 4]). Therefore, to understand how sensorimotor circuits link perception to action, it is necessary to monitor neural activity and behavior simultaneously. In larval zebrafish, the small size and optical transparency of the nervous system allows functional imaging of neural activity at cellular resolution and throughout the brain, during behavior [5–7]. In this study, we used two-photon (2P) calcium imaging to examine how perception of prey-like visual cues leads to initiation of hunting.

In larval zebrafish, prey catching is a visually guided behavior [8–10]. Several studies have examined the locomotor and oculomotor components of hunting routines including the kinematic features of orienting turns (described as J-turns in [9]), capture swims [8, 11], and coordinated pectoral fin movements [12]. Of particular relevance to this study, zebrafish larvae perform a specialized oculomotor behavior, eye convergence, specifically during hunting. A convergent saccade defines the onset of all hunting routines, and the eyes maintain a high vergence angle until after the strike at prey [13]. After the initial convergent saccade, vergence angle further increases during prey tracking, in relation to target proximity [11]. By increasing the extent of the binocular visual field and advancing it close to the nose of the animal, eye convergence might enable a stereopsis mechanism for judging target distance and triggering the final capture event [13].

The optic tectum (OTc) is the largest retinorecipient structure in the brain of teleost fish and is likely to be of central importance for hunting behavior. Visual space is retinotopically mapped across the OTc in register with the tectal motor map and as such the OTc is well suited to control goal-directed behaviors toward specific points in space [14]. These include orienting and avoidance behaviors [15], saccadic eye movements [16], and prey-catching behaviors including striking at prey [17]. Indeed, neural activity in the OTc of larval zebrafish was recently observed in response to live prey [18]. Zebrafish hunting is greatly reduced by ablating the retinal input to the tectum [10], silencing a specific population of tectal interneurons [19], or a genetic mutation that disrupts the spatial and temporal fidelity of retinotectal transmission [20]. Larvae respond to prey located within the frontal region of visual space (the "reactive perceptive

CrossMark

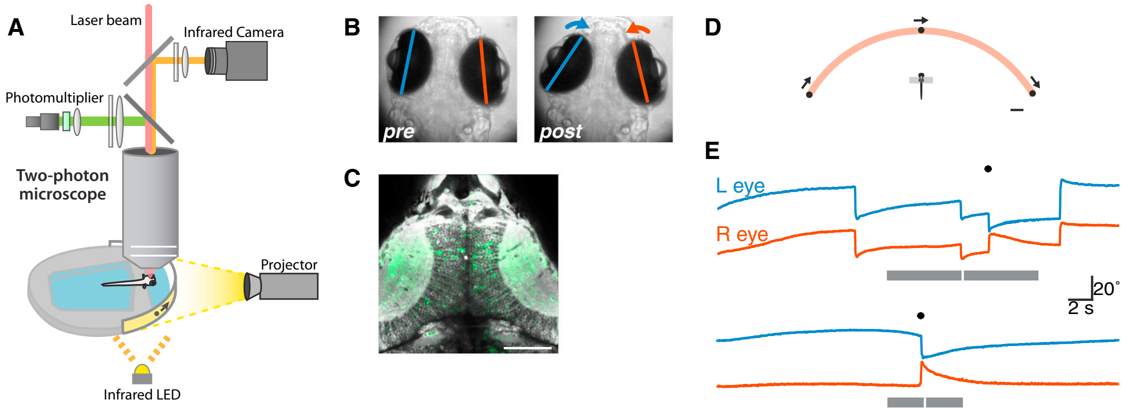

Figure 1. 2P Functional Calcium Imaging during Virtual Hunting Behavior in Larval Zebrafish

(A) Schematic of experimental setup. Larval zebrafish are tethered in agarose gel but able to freely move their eyes and tail. Visual stimuli are presented by projection onto a miniature screen in front of the animal. A 2P microscope is used to image fluorescent calcium signals, and eye position is monitored simultaneously through the microscope objective using an infrared camera.

(B) Eye position recorded before (left) and after (right) a predatory convergent saccade, during 2P imaging.

(C) Neural activity recorded in the optic tecta of a Tg(elavl3:GCaMP5G) transgenic larva. The fractional change in fluorescence (Δ F/F) is shown in green (arbitrary color scale) overlaid on an anatomical projection of the focal plane (gray). This field-of-view corresponds to FOV2 as shown in Figure 3A. Dorsal view, anterior top. Scale bar, 50 μm.

(D) Schematic of the behavioral assay (viewed from above). The animal is presented with visual stimuli that are projected onto a screen covering ~200° visual space. Scale bar, 2 mm.

(E) Examples of eye-position records from two trials in two different larvae. The gray bar indicates the period during which the visual cue sweeps across visual space from +100° right to −100° left (top) or left-right (bottom). The different lengths of the bars correspond to different speeds of stimulus motion. The white tick indicates the time when the cue is at 0°, directly in front of the animal. The black symbol indicates the automatic detection of a convergent saccade. Downward deflection of eye position traces corresponds to clockwise eye rotation.

field” [13]), which is represented in the anterior portion of the visuotopic tectal space map [14, 21]. Notably, optogenetic stimulation of the anterior-ventral OTc is sufficient to evoke convergent saccades and J-turns [22]. By contrast, projection neurons in the posterior tectum have been reported to be dispensable for prey catching [19].

In this study, we performed functional imaging in the anterior tectum of tethered larval zebrafish, while the animal engaged in virtual hunting behavior that was evoked by presentation of artificial visual cues [13]. By systematically varying four features of the visual stimuli, we found that prey-catching behavior was selectively evoked by specific conjunctions of visual features. Unbiased clustering of visual response vectors revealed that tectal neurons show mixed selectivity for multiple stimulus features. Furthermore, we could identify cells that showed non-linear mixed feature selectivity that closely matched the stimulus tuning of hunting responses. To investigate how activation of these feature-analyzing neurons might be linked to initiation of prey-catching behavior, we compared neural activity in response trials versus non-response trials. This enabled us to uncover tectal population activity that was specifically associated with hunting responses. Assemblies of tectal neurons produced bursts of activity in advance of, or concurrent with, the initiation of behavior, were confined to the left or right tectal hemisphere and their laterality correlated with asymmetries in the oculomotor parameters of convergent saccades. Consequently, these population dynamics likely represent premotor activity controlling the release of hunting responses. In summary, by imaging neural activity at cellular resolution during naturalistic behavior, we have functionally identified circuit components that are likely to mediate the perceptual recognition of ethologically relevant stimuli and the release of an adaptive behavioral response.

RESULTS

Functional Calcium Imaging during Tethered Hunting Behavior in Larval Zebrafish

To monitor neural activity during the recognition of prey-like visual cues and the initiation of hunting routines, we combined a virtual hunting assay for tethered larval zebrafish [13] with in vivo 2P functional imaging in transgenic larvae expressing a genetically encoded fluorescent calcium indicator under the control of a pan-neuronal promoter, Tg(elavl3:GCaMP5G) a4598 [23] (Figure 1).

In our assay, larval zebrafish were tethered in agarose gel but able to freely move their eyes and tail, and visual cues were projected onto a diffusive screen in front of the animal (Figures 1A and 1D). We previously showed that larvae respond to simple moving spots with hunting-associated oculomotor and locomotor behaviors [13]. Specifically, larvae perform a convergent saccade and an orienting turn, comprising multiple unilateral tail bends directed toward the visual target. The kinematics of these behaviors closely match those observed for freely swimming fish hunting live prey. Because every hunting routine (for both free swimming and tethered larvae) commences with eye convergence, and the spontaneous rate of convergent saccades is very low, we could use eye tracking alone to determine that the animal has initiated hunting behavior in response to a visual target (Figures 1B and 1E). Furthermore, the relatively high failure

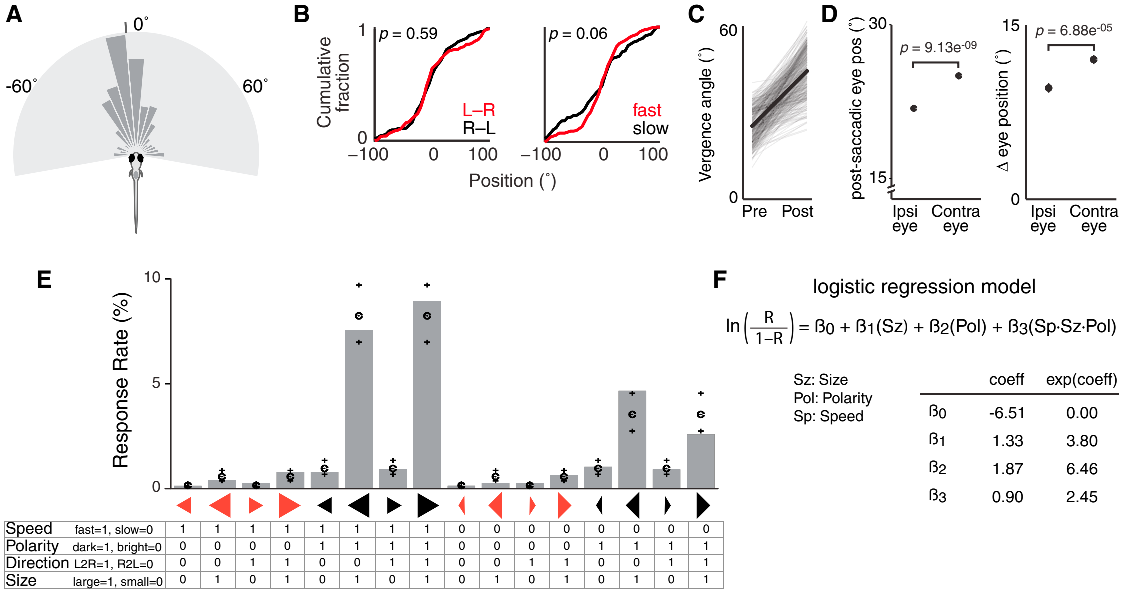

Figure 2. Hunting Responses Show Mixed Selectivity for Stimulus Feature Compounds

(A) Distribution of spot locations at time of convergent saccade (n = 361 events in 48 fish). Tick indicates median location, −5.56°.

(B) Distribution of spot locations at time of convergent saccade did not differ for left-right versus right-left stimuli (left) nor for slow versus fast stimuli (right). Note that, to compare slow versus fast stimuli, all spot locations were simulated as moving left-right.

(C) Change in ocular vergence angle during convergent saccades. Thick line shows mean.

(D) The eye contralateral to the visual hemifield in which the spot was located at the time of the convergent saccade showed a greater change in eye position (nasal rotation). Left: post-saccadic eye position. Greater values indicate more nasal position. Right: change in eye position. Data are shown as mean ± SEM.

(E) Response rates for the 16 moving spot stimuli (236 events in 27 fish). Black spots indicate response rates predicted by the logistic regression model (crosses indicate 95% confidence interval [CI]). Symbols below the x axis indicate the features of each moving spot stimulus: leftward arrow, right-left; rightward arrow, left-right; large symbol, large; small symbol, small; elongated arrow, fast; short arrow, slow; red, bright; black, dark. The table indicates the binary coding scheme by which each stimulus is coded in terms of four feature values.

(F) Logistic regression model that best explained the variance in response rate, *R*, as a function of stimulus features. The inset table shows the estimated values of the coefficients as well as the exponentiated coefficients (also known as odds ratios). The fit coefficients (with 95% CI and p values) were $\beta_0 = -6.51$ [−7.10, −5.92], $p = 3.13 \times 10^{-127}$; $\beta_1 = 1.33$ [0.87, 1.80], $p = 2.72 \times 10^{-10}$; $\beta_2 = 1.87$ [1.34, 2.39], $p = 1.48 \times 10^{-14}$; $\beta_3 = 0.90$ [0.54, 1.24], $p = 3.22 \times 10^{-8}$.

rate of stimulus-evoked hunting responses (5%–10% response rate for the best stimuli) allowed us to disambiguate visually evoked activity from neural activity related to the release of a behavioral response.

During the virtual hunting assay, we performed 2P calcium imaging to monitor neural activity in the optic tecta (Figure 1C). Hunting responses generated only small motion artifacts that could be corrected during post-processing (see the Experimental Procedures), and 2P imaging had no apparent detrimental impact on larval health or behavior. This approach therefore enabled us to monitor neural activity during the sensorimotor transformations linking the recognition of prey-like visual objects to the initiation of a hunting response.

Virtual Hunting Assay

To examine the stimulus tuning of hunting responses, we presented a panel of moving spot stimuli that differed in terms of four stimulus features: direction, size, speed, and contrast polarity. For each feature, we tested two values, for a total of 16 unique stimuli. Specifically, moving spots could differ in direction (left-right or right-left motion), speed (fast 30°/s or slow 15°/s), size (small 3.5°, large 13.2°), or contrast polarity (a bright spot on a dark background, or a dark spot on a bright background) (see the Experimental Procedures). Stimuli were presented in the frontal portion of visual space, spanning the region where freely swimming larvae attend to live prey (approximately −60° (left) to +60° (right) [13]). Thus, moving spots appeared at 100° to the left or right of the animal's extended midsagittal plane and then swept 200° to the right, or left, respectively.

Behavioral responses indicating the initiation of hunting routines were defined as convergent saccades in which both eyes rotated nasally (Experimental Procedures) (n = 361 events in 48 fish). Larvae responded to stimuli most frequently when they were almost directly ahead (median azimuth, −5.56° [left], Wilcoxon signed rank test versus median of 0°, p = 0.19; Figure 2A). There was no significant difference in the spatial location of targets at the time of convergent saccades for left- versus rightward-moving spots (p = 0.59, Kolmogorov-Smirnov test; Figure 2B), nor for slow- versus fast-moving spots (p = 0.06).

Convergent saccades increased ocular vergence angle by 19.03° ± 0.49° (mean ± SEM), with mean vergence angle after saccade of 44.4° ± 0.43°, similar to our previous study [13] (Figure 2C). The eye contralateral to the stimulus at the time of the saccade tended to show a larger nasal rotation and adopted a more nasal post-saccadic position (in agreement with [24]). For example, convergent saccades triggered by visual cues located on the right, usually involved greater rotation of the left

eye ($p = 9.13 \times 10^{-9}$, contralateral versus ipsilateral eye position after the saccade; $p = 6.88 \times 10^{-5}$, contralateral versus ipsilateral change in eye position, paired t tests; Figure 2D).

In summary, our behavioral assay allowed us to present visual cues to tethered larval zebrafish to evoke oculomotor responses associated with the initiation of natural hunting routines, during 2P functional imaging.

Hunting Responses Are Selective for Visual Feature Compounds

The probability of evoking hunting responses varied substantially across our panel of visual stimuli. We quantified response rate (*R*) as the proportion of stimulus presentations that evoked a convergent saccade (Figure 2E). The most effective stimuli were spots for which polarity was inverse (dark spots) and size was large. Fast-moving large, dark spots were also more effective than otherwise identical slow-moving stimuli. These results suggest that hunting responses are sensitive to multiple stimulus features.

We used logistic regression to model the relationship between response rate and the four visual features. For each type of feature, we used a binary coding scheme to represent the two feature levels (e.g., fast [1], slow [0]) such that each stimulus was described by a vector of four binary values (Figure 2E, bottom table). Using stepwise regression, we identified the model described in Figure 2F as producing the most accurate description of the data. To compare alternative models, we used a cross-validation approach in which we fit model coefficients on half the data set (training set) and assessed model predictions against the other unseen half (test data set) to estimate a cross-validated R^2 (Experimental Procedures). The model in Figure 2F had a cross-validated R^2 of 0.82 and indicates that hunting responses are strongly modulated by size and contrast polarity. Large stimuli increase the odds of response by 3.8-fold (given by $e^{\beta 1}$) and dark stimuli by 6.5-fold ($e^{\beta 2}$). In addition, the interaction term in the model indicates that, when the stimulus is both dark *and* large *and* fast, the odds of a response are increased by 2.5-fold ($e^{\beta 3}$).

We conclude that larval zebrafish respond differentially to moving visual cues as a function of multiple stimulus features and are sensitive to the coincidence of particular feature values (feature compounds). Specifically, size, contrast polarity and speed of motion interact, such that stimuli that are large, dark, and fast are most effective in triggering hunting responses.

Visual Response Properties in the Optic Tectum and Adjacent Regions

To investigate how different stimuli—and individual stimulus features—are encoded by neural activity, we performed 2P calcium imaging in the rostral portion of the optic tecta (and adjacent regions) (Figure 3A). In addition to the 16 moving spot stimuli, we included two control stimuli, which were 3 s "whole-field" light flashes at two different intensities. We imaged activity at ten to 15 dorsoventral levels and at each focal plane presented five to eight repetitions of each of the 18 stimuli, in a pseudorandom sequence, while simultaneously monitoring behavior.

To characterize the visual response profiles of individual neurons, we computed a visual response vector for each cell as follows. First, imaging planes were automatically segmented to define regions-of-interest that corresponded well to single somata (Figure S1). Regions of interest (ROIs) localized to the synaptic neuropil layers of the OTc were excluded. Next, we computed the mean fluorescent calcium signal ($\Delta F/F$) across the repeated presentations of each visual stimulus and finally concatenated these average responses to produce a visual response vector (VRV). The VRV therefore summarizes the visual responses of each neuron in the form of the full response time course to the 18 visual stimuli (684 time points per cell).

To examine the diversity of visual response profiles, we used an unbiased clustering method to group visually responsive cells from 14 fish based on the similarity of their VRVs, as measured by correlation (Experimental Procedures). Our method produced 20 clusters, each of which contained cells from a minimum of six fish (Figure 3B; Figure S2; Table S1). These clusters contained neurons with more coherent visual tuning properties than we could obtain using *k*-means clustering. From a total of 169,371 ROIs (14 fish), our method clustered only 5,092 visually responsive cells (~3%). This relatively small sample set is most likely not exhaustive but allowed us to identify groups of neurons with feature selective visual tuning that were found consistently across multiple fish. Notably, an alternative clustering approach based on Gaussian mixture modeling identified very similar clusters but also isolated only a relatively small number of cells (1,035 cells from 101,656 in 10 fish, ~1%; Figure S3; Experimental Procedures). Figure 3B shows the 20 clusters identified using our correlation-based clustering approach to measure the similarity of VRVs, at a minimum correlation coefficient threshold of 0.75 (see Figure S2 and Table S1 for additional cluster details).

Clusters could be broadly divided into those modulated by changes in background luminance and clusters selective for moving spots. A step increase in background luminance occurs during presentation of negative polarity (dark) moving spots (starting 2 s before spot appearance and ending 2 s after spot disappearance; Experimental Procedures), as well as during the control light-flash stimuli. The six clusters responsive to changes in background luminance (c15–20) showed a diversity of response properties and temporal dynamics. These include negative modulation (a decrease in fluorescence signal, which we presume corresponds to a decrease in tonic firing rate) in response to an increase in luminance (cluster 20): constituent cells were found in the habenulae and torus longitudinalis (TL) as well as the optic tecta (Figure 3C). Cluster 19 showed positive modulation in response to decreases in luminance ("dimming detectors"), and cluster 16, which contained the largest number of neurons of any cluster, displayed positive modulation in response to increasing whole-field luminance ("ON" response). This was evident in the response to changes in background light level during control stimuli and dark spot presentations and in response to large, bright, moving spots. A large proportion of these neurons (41%) were located in the TL, specifically at its rostral pole (Figure 4A).

Tectal Neurons Are Selective for Multiple Stimulus Features

We identified 14 clusters that were responsive to moving spots and showed minimal modulation to changes in background luminance. Inspection of cluster centroids (the average VRV of

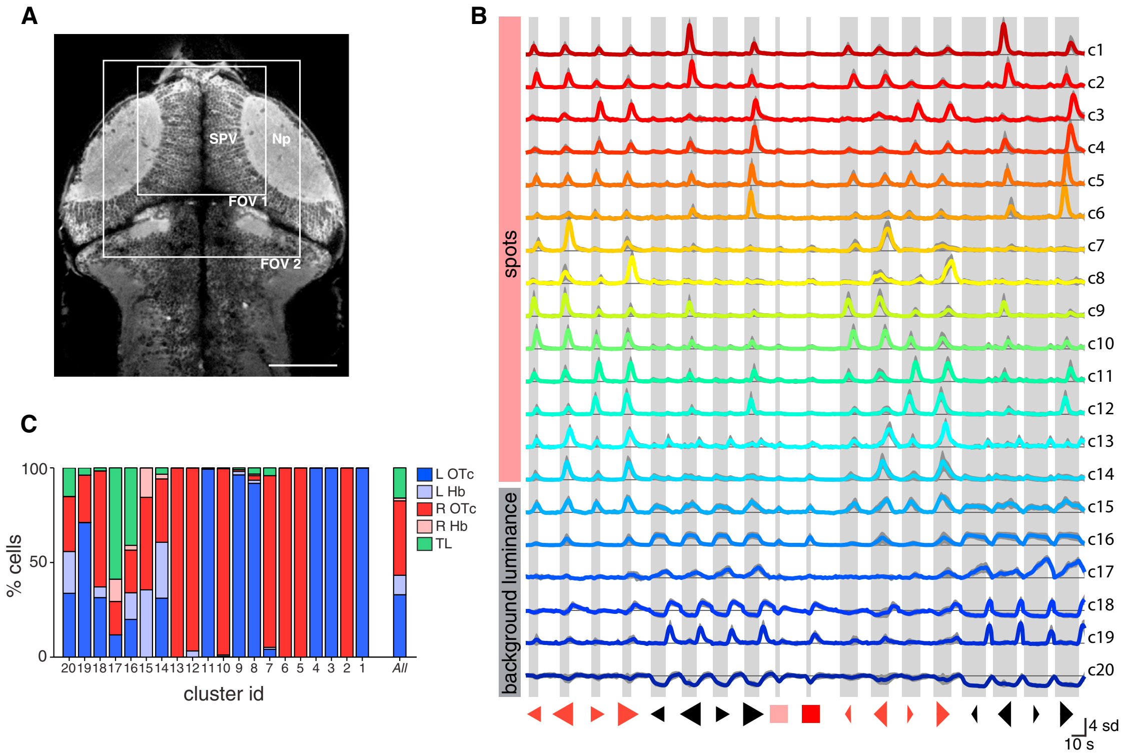

Figure 3. Clustering of Visual Response Properties

(A) 2P focal plane showing a dorsal view of the brain of a 5 dpf Tg(elavl3:GCaMP5G) larva. Boxes indicate size and approximate locations of fields of view for functional imaging of the anterior optic tecta. The stratum periventriculare and synaptic neuropil regions of the right OTc are labeled. Anterior top. Scale bar, 100 μm.

(B) Cluster centroids (mean visual response vectors) of 20 clusters of visually responsive neurons from 14 fish. For each cluster, the visual response vectors of constituent cells were divided by their SD (to normalize responses across cells with varying magnitudes of signal modulation), and the mean visual response vector was computed (colored lines). Thin black lines indicate zero ΔF/F. Gray shading indicates SD across cells. Numbers on the right are cluster IDs. Shaded bars indicate visual stimulus presentation periods.

(C) Anatomical distribution of cells from each cluster. L, left; R, right; Hb, habenula; OTc, optic tectum; SPV, stratum periventriculare; Np, tectal neuropil; TL, torus longitudinalis.

See also Figures S1–S3 and Table S1.

cells within the cluster) revealed that clusters respond differentially across the panel of 16 moving spot stimuli and show direction, size, and polarity selectivity (Figure 3B; Table S1). We quantified feature tuning by computing, for each cell, four selectivity indices (for direction, speed, size, and polarity) based on the maximal mean calcium signal across the panel of 16 stimuli (Experimental Procedures).

Mirror-symmetric clusters could be identified in the left and right tectal hemispheres, with similar feature tuning. For example, clusters 9–12 show size, polarity, and direction selectivity, with a net preference for large, bright spots moving either leftward or rightward (Figures 4B and 4C). Clusters 9 and 10 prefer right-left-moving spots. Despite otherwise similar tuning, clusters 9 and 10 were segregated because they respond at different times to spots sweeping across the visual field, from +100° (right) to −100° (left). The retinotectal projection is entirely crossed in larval zebrafish such that the left OTc is innervated by retinal ganglion cells deriving from the right eye and the right OTc receives input from the left eye. Accordingly, cluster 9 is exclusively located in the left OTc and responds earlier during presentation of right-left visual cues (moving tail-nose), whereas cluster 10 is confined to the right OTc and responds later, after the cue has crossed to the left visual hemifield (nose-tail motion; Figure 4B). Clusters 11 and 12 show the opposite direction selectivity (preferring left-right motion) and are similarly located in the left and right OTc, respectively (Figure 4C). Consequently, clusters 9 and 12 form a mirror-symmetric pair tuned to tail-nose motion, located on the left and right, respectively. Clusters 10 and 11 form a second pair tuned to nose-tail-moving spots.

Hunting responses were evoked most frequently by large, dark, fast-moving spots. Our unbiased clustering procedure identified tectal neurons tuned to large, dark spots, which additionally showed direction selectivity. Clusters 1 and 2 show a preference for leftward-moving large, dark spots and are located in the left and right OTc, respectively (Figure 4D). Clusters 3–6 show the opposite direction selectivity, preferring rightward motion. These four clusters have similar tuning and were divided not only by tectal laterality, but also by rostrocaudal tectal location, based on the differential timing of their calcium responses

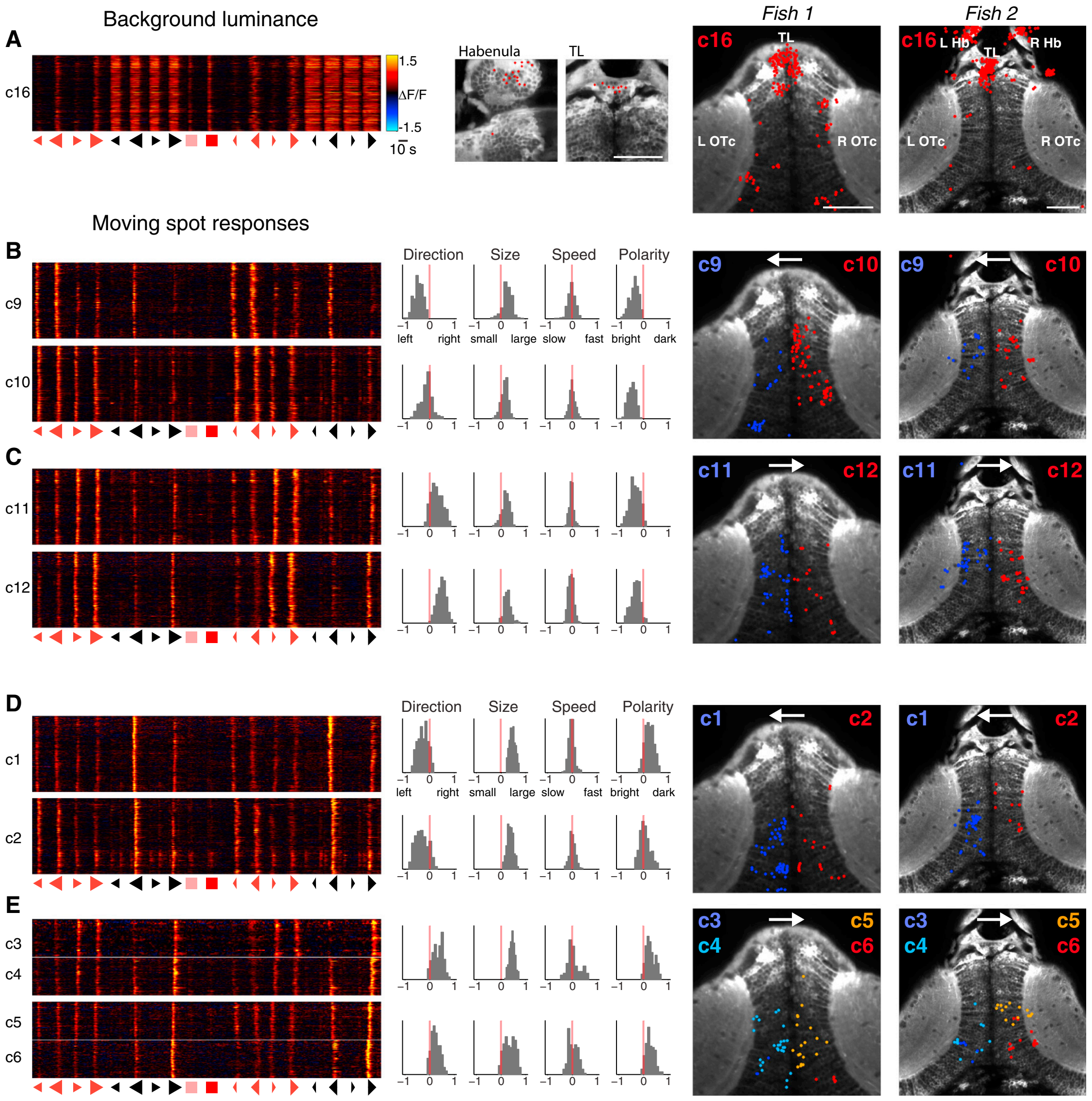

Figure 4. Clusters Respond to Background Luminance Changes and Show Mixed Selectivity to Multiple Features of Moving Spot Stimuli

(A) Cluster 16 shows positive modulation in response to increases in whole-field luminance. The left panel shows VRVs for every cell in the cluster (each row corresponds to one cell). Neurons show increased calcium signals in response to the increased background luminance that occurs in conjunction with presentation of dark, moving spots, as well as in response to large bright spots and whole-field light flashes. Middle panels show locations of cluster 16 cells in the habenula and torus longitudinalis, and right panels show anatomical locations of all detected cluster 16 neurons in two representative larvae. Neuron locations are marked as x-y centroids (colored spots) overlaid on a single anatomical image from the dorsoventral mid-point of the imaging volume. Many cluster 16 cells are located in the torus longitudinalis and the habenulae. Scale bars, 50 μm.

(B and C) Mirror-symmetric pairs of clusters showing a net preference for large, bright, moving spots. (B) Clusters 9 and 10 are tuned to leftward-moving stimuli and localize to the left and right optic tecta, respectively. (C) Clusters 11 and 12 are tuned to rightward-moving stimuli. Histograms show distributions of feature selectivity indices for cells in each cluster. Arrows on anatomical maps indicate preferred direction of motion.

(D and E) Clusters responding to large, dark, moving spots. (D) Clusters 1 and 2 are tuned to leftward-moving large, dark spots. (E) Clusters 3–6 prefer rightward-moving stimuli. These clusters were divided based on differential response times, corresponding to different spatial receptive field locations. Accordingly, they occupy different positions in the tectal space map.

(Figure 4E). In accordance with the retinotopic mapping of visual space, clusters at more caudal positions (clusters 3 and 6) responded when visual cues were at more peripheral locations. Our expectation is that other clusters (e.g., clusters 1 and 2) could be similarly subdivided if temporal resolution was higher or the correlation threshold of our clustering procedure was increased.

In conclusion, we find that tectal neurons show mixed selectivity and are sensitive to combinations of visual features (feature compounds). These include direction-selective cells with a preference for large, dark, moving spots that we found to be among the most effective stimuli in evoking hunting responses.

Non-linear Mixed Selectivity Neurons Are Tuned to Optimal Prey-like Visual Stimuli

Hunting responses displayed mixed feature selectivity and were most effectively triggered by large, dark, fast-moving spots. Based on these behavioral observations, we developed an approach to specifically search for neurons that could mediate detection of preferred prey-like visual targets. In addition, we sought to quantitatively describe neural activity as a function of the four visual features to determine if individual neurons are selective for the conjunction of multiple features in a similar way to whole animal behavior.

In the first part of our approach, we designed a panel of simple binary "regressors" to search for cells with visual tuning profiles similar to the behavioral tuning. All regressors were selective for large, dark stimuli. Although behavioral response rates do not show net direction selectivity, we recognized that this could result from the combined action of two or more populations of direction-selective neurons that mediate responses to prey moving in different directions. Importantly, we found the clusters with preference for large, dark spots to be direction selective (see above). Therefore, we included regressors that either did or did not include this property. In the logistic regression model describing response rate in terms of stimulus features, the interaction term involving speed had the smallest coefficient. For this reason, as well as the fact that the unbiased clustering did not isolate speed-tuned cells, we designed regressors with and without speed tuning. The six regressors were as follows: non-direction selective, non-speed selective (nDS-nSp); non-direction selective, speed selective (nDS-Sp); leftward selective, non-speed selective (R2L-nSp); leftward selective, speed selective (R2L-Sp); rightward selective, non-speed selective (L2R-nSp); and rightward selective, speed selective (L2R-Sp) (Figure 5A).

Next, we used these regressors to identify ROIs with similar stimulus tuning. We considered ROIs located in the tectal neuropil as well as those corresponding to cell bodies in the stratum periventriculare (SPV) and regions adjacent to the OTc. For every ROI, we computed the correlation coefficient between a vector describing the peak average response to each of the 16 moving spot stimuli and each of the regressors. ROIs were associated with the regressor that yielded the highest correlation coefficient when that coefficient was 0.75 or greater.

Figure 5A shows the VRVs for all of the ROIs that were associated with each of the six regressors, from ten fish. Visual inspection of the VRVs indicates that the isolated cells are tuned to large, dark, moving spots. More ROIs were isolated by the non-speed-selective regressors and the largest number was associated with the nDS-nSp regressor. However, an appreciable number of ROIs showed selectivity for fast motion, responding most strongly to the large, dark, fast-moving stimuli. Notably, these highly stimulus-selective tuning profiles were apparent for single voxels from such ROIs (Figure S4).

To quantitatively describe these tuning profiles, we used generalized linear regression to model the responses of each ROI as a function of stimulus features. We used cross-validation to compare two models: a linear mixed selectivity model and a non-linear mixed selectivity model with interaction terms designed to capture the responses of neurons tuned to large, dark, moving spots, which may or may not also display direction selectivity and/or speed tuning (Figure 5B). For the majority of ROIs, stimulus tuning was better described by the non-linear mixed selectivity model (for 86% ROIs, cross-validated R^2_{nlin} was greater than R^2_{lin}, $p \ll 0.0001$, Wilcoxon signed rank test). We further quantified this by computing an index, SI_{nlin}, based on the relative ability of the linear and non-linear models to explain the variance of the responses (Experimental Procedures). The superior performance of the non-linear model was true for the majority of ROIs associated with each of the six regressors (Figure S4B) as well as for the entire population pooled across regressors (Figure 5F). Responses to the 16 stimuli predicted by the non-linear model showed good agreement with measured values (Figure S4A).

Figure 5C shows the non-linear model coefficients obtained for ROIs associated with each regressor. Coefficients for the model terms that are expected to define the response properties of each group show non-zero values of the expected sign. For example, neurons selective for large, dark spots but which are neither speed nor direction selective, should be well described by the *size*polarity* interaction term. Indeed, for the population of nDS-nSp cells, only this coefficient shows a non-zero value. By comparison, for right-left-tuned cells that also show a preference for fast spots (R2L-Sp), the *speed*size*polarity* term should have a positive coefficient (such that the cells respond to fast (1), large (1), dark (1) stimuli that are encoded as 1's in our binary coding scheme; Figure 2E). So that direction selectivity can be conferred, the *direction*speed*size*polarity* term should also be non-zero, with a negative coefficient, to suppress responses to rightward-moving stimuli (rightward motion is coded as 1). This is what we observed. We conclude that our method identified ROIs showing non-linear mixed selectivity (NLMS), tuned to conjunctions of multiple feature values. Almost all of these ROIs (99.6%) were located in the tectal cell body layers or neuropil, with very few in adjacent structures (habenula, TL) (Figure 5G).

Selectivity indices, for the four individual stimulus features, confirmed that NLMS ROIs preferentially respond to large, dark, moving spots (Figure 5E), and ROIs associated with each regressor showed the expected pattern of speed- and direction selectivity (Figure 5D). After correcting for eye position, we estimated the spatial receptive fields (RFs) of individual NLMS neurons in the tectal SPV and found that they spanned the frontal region of visual space, with a high density of RF centers in the central region where visual cues evoked the highest proportion of hunting responses (Figures 5H and 5I).

In summary, the OTc contains highly tuned neurons displaying non-linear mixed selectivity for specific conjunctions of visual features. We identified NLMS neurons that preferentially respond to large, dark, fast-moving spots, which are the visual stimuli that were most effective in evoking hunting responses. These neurons are therefore candidates for mediating the perceptual recognition of optimal prey-like visual objects.

Tectal Activity Associated with Initiation of Hunting Behavior

What are the neural substrates linking sensory perception of prey-like cues to the release of prey-catching behavior?

We investigated whether tectal activity was associated with execution of the first motor output that defines initiation of hunting behavior, namely, a convergent saccade. To do this, we took

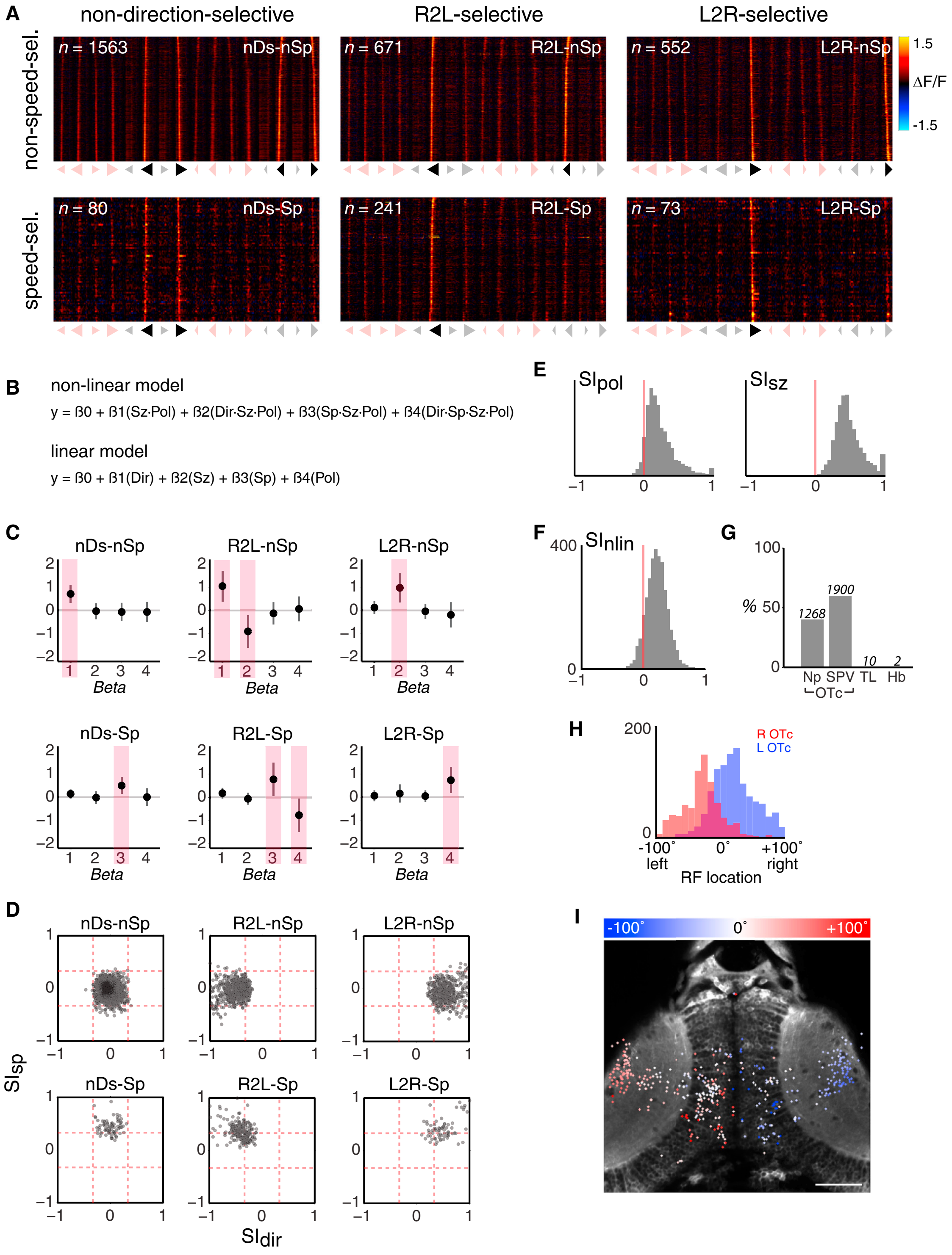

Figure 5. Tectal Neurons Show Non-linear Mixed Selectivity for Best Prey-like Stimuli

(A) Visual response vectors (VRVs) of ROIs isolated using six regressors that were designed based upon the stimulus tuning of behavioral response rates. Symbols below each panel indicate the stimuli coded 1 in the binary vector defining the regressor. Stimuli coded 0 are shown in light shading. ROIs were associated with the regressor that produced the highest correlation coefficient, when that coefficient was 0.75 or greater.

(B) Non-linear and linear models used to fit the response profiles of individual ROIs. *y* represents the fluorescence response (peak ΔF/F during stimulus presentation). Each stimulus is coded as a set of four binary feature values, as shown in Figure 2E.

(C) Coefficients (β's) derived from fitting the non-linear model in (B) to ROIs associated with each regressor. Shaded bars indicate coefficients expected to show significant non-zero values. Data are shown as mean ± SD.

(legend continued on next page)

advantage of our online behavioral data, which allowed us to track eye movements during 2P functional imaging (Figure 6A). We compared the activity of individual tectal neurons in response trials versus non-response trials to identify cells that showed a significant increase in fluorescence signal associated with the release of hunting responses (Figure 6B). Specifically, we considered two eye convergence-triggered time windows: the "pre-conv" window compared activity during 1.65 s immediately prior to the convergent saccade to activity at corresponding times in non-response trials. This window was designed to identify tectal neurons with premotor activity, which might be involved in initiating hunting responses. The second window, "peri-conv," compared activity during 2.75 s centered on the convergent saccade. This enables identification of neurons that modulate activity coincident with an eye convergence. For both windows, we used t tests to compare activity (ΔF/F) in response versus non-response trials. Furthermore, we evaluated the maximum signal-to-noise ratio (SNR) of the cell's response within the same window. We considered cells to show response modulation when $p < 0.05$ and SNR >3.

Tectal Assemblies Represent an Activity Motif Associated with Convergent Saccades

Maps of response-modulated cells revealed spatially grouped clusters, or *assemblies*, of tectal neurons located in discrete regions of the stratum periventriculare (SPV) and confined to either the left or right tectal hemisphere. Three examples of assemblies identified from the pre-conv analysis are shown in Figures 6C–6H (see also Figure S6). The assembly shown in Figures 6C and 6D comprises a spatially clustered population of neurons in the right OTc, which shows a burst of activity after visual stimulus onset and immediately preceding execution of a convergent saccade. Many of the neurons showed no detectable activity in response to the same visual stimulus in non-response trials (see examples in Figure 6D). However, all the constituent cells were classed as visually responsive, indicating response modulation to at least one of the 18 stimuli in our test set (Experimental Procedures). Cells within the assembly showed correlated patterns of activity during the response trial. We quantified this by calculating the average correlation of each cell's fluorescence time course with the mean of the assembly, r_{avg} = 0.93 [0.89, 0.95] (median, interquartile range).

We identified 30 pre-conv assemblies from a total of 304 convergence events in 19 larvae. Assemblies were automatically detected by fitting an ellipse to the spatial distribution of response-modulated cells and were defined as unilateral clusters containing a minimum of six cells at a minimal density of 533 μm^2/cell (Experimental Procedures). Only cells in the tectal SPV were considered for this analysis, and ROIs in the neuropil were excluded. Note, however, that response-modulated ROIs were often observed in the neuropil adjacent to active assemblies (Figures 6C–6J). At the population level, pre-conv assemblies increased activity in advance of convergent saccades by an average of 1.65 s [1.1, 2.2] (median, interquartile range) (Figure 6O). The average intra-assembly correlation of individual cells with the mean assembly response was 0.71 [0.65, 0.81] (median, interquartile range), and the percentage of constituent cells classified as visually responsive was 82% [71, 100] (median, interquartile range) (Figure S5). Notably, pre-conv assemblies contained few, if any, NLMS cells (median = 0, mean = 0.6, n = 223 cells in 14 assemblies) suggesting prey detection and the initiation of predatory responses are mediated by largely non-overlapping neuronal populations in the OTc.

The peri-conv analysis identified tectal neurons that showed significantly elevated activity during response trials within a time window centered on convergent saccades and revealed that such cells were also grouped into anatomically discrete assemblies (88 assemblies from 304 convergent events in 19 larvae). The example in Figures 6I and 6J shows such an assembly in the left OTc. Cells in this assembly predominantly increased activity in the same imaging frame as the convergent saccade, with activity peaking in the subsequent frame. Indeed, this periconv analysis allowed us to discover assemblies in which population activity started concurrent with, or subsequent to, the convergent saccade. By manually examining individual imaging movies, we were careful to ensure that this did not result from motion artifacts that escaped our registration procedure. Such post-saccadic activity might represent an efference copy of a saccadic motor command. For peri-conv assemblies, population activity preceded the convergence event by an average of 0.55 s [0, 1.1] (median, interquartile range), with many assemblies showing onset of activity concurrent with or in the first frame after eye convergence (Figure 6O). As with pre-conv assemblies, constituent neurons showed highly correlated activity during the response. The average intra-assembly correlation with the mean response was 0.76 [0.68, 0.82] (median, interquartile range). Again, the majority of constituent cells were classified as visually responsive: 89% [69, 100] (median, interquartile range).

To assess whether tectal assemblies are specifically associated with convergent saccades or could represent spontaneous ongoing activity that occasionally coincides with hunting responses, we estimated a false discovery rate. To do this, we constructed artificial response and non-response epochs by circularly permuting the fluorescence time-series data and detected assemblies using identical criteria to those used for the original data. From the average of five permutations, we estimate false discovery rate to be approximately 10%–20% (18.7% pre-conv and 8.9% peri-conv assemblies) (Figures 6K

(D) Direction- and speed-selectivity indices (SI_{dir}, SI_{sp}) for ROIs associated with each regressor.
(E) Polarity- and size-selectivity indices (SI_{pol}, SI_{sz}).
(F) Comparison of model fits (R^2) obtained with non-linear versus linear mixed selectivity models, quantified as a selectivity index, SI_{nlin}. The majority of ROIs show a positive index, indicating the non-linear model provides a more accurate description of the variance of their responses.
(G) Anatomical distribution of ROIs. Values above each bar indicate number of ROIs.
(H) Distribution of receptive field (RF) centers for NLMS neurons in the left (blue) and right (red) optic tecta. Only ROIs localized to the tectal SPV are shown.
(I) Anatomical map of all NLMS ROIs in one example fish. Each spot indicates the centroid of an ROI, color-coded according to estimated RF location. ROIs located in both the tectal SPV and neuropil regions are shown. Note that for presentation, ROIs from the entire imaging volume are overlaid on a single anatomical image from the dorsoventral mid-point of the volume.
Scale bar, 50 μm. See also Figure S4.

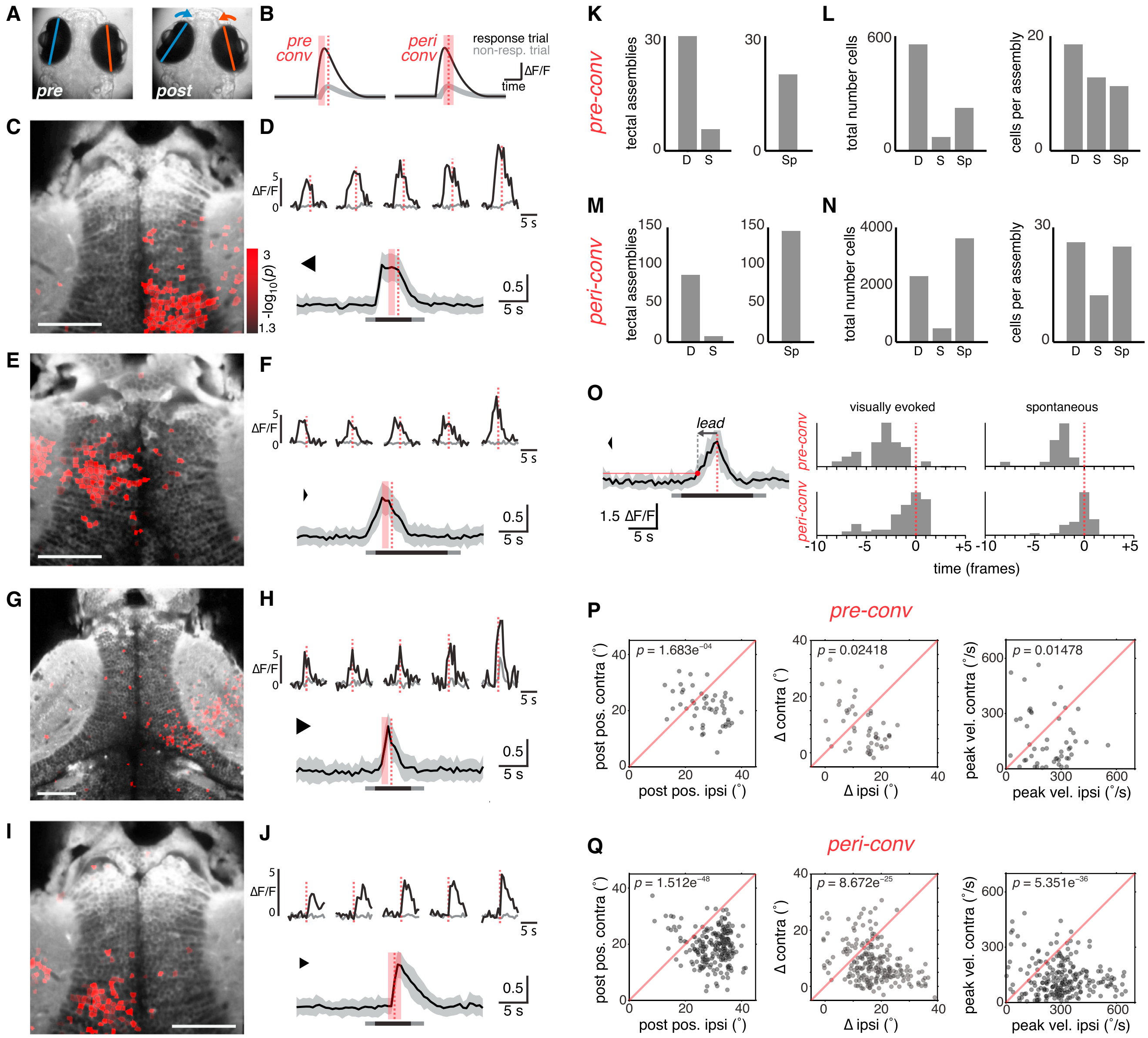

Figure 6. Assemblies of Tectal Neurons Show Premotor Activity Associated with Convergent Saccades

(A) Eye position before and after a convergent saccade.

(B) Schematic indicating time windows used to identify ROIs with a significantly greater GCaMP response immediately prior to a convergent saccade ("pre-conv", left) or around the time of the saccade ("peri-conv", right). Dashed red line indicates time of eye convergence in response trial. Shaded red bars indicate time windows during which activity (ΔF/F) was compared (t test) between response (black) versus non-response trials (gray).

(C) Example of right tectal assembly that was active in advance of a convergent saccade ("pre-conv"). Response-modulated ROIs (red, color map indicates log-transformed p value from t test) are overlaid on an anatomical projection (gray).

(D) Top: five cells from the assembly in (C). Activity in response trial in black, mean activity in non-response trials in gray. Bottom: activity of all cells in assembly in (C) during the response trial. To facilitate comparison of cells with different response amplitudes, we normalized ΔF/F time courses to the maximum value for each cell before computing the population average, shown as mean ± SD. Bar indicates visual stimulus presentation (light gray, change in background luminance; dark gray, moving spot presentation).

(E–H) Two more examples of "pre-conv" assemblies that were active in advance of convergent saccades. Assemblies are from two different fish that are also different from (C) and (D).

(I) Left tectal assembly that was active around the time of a convergent saccade ("peri-conv"). Same fish as (C) and (D).

(J) Responses of individual cells (top) and the whole assembly (bottom) shows activity coincident with, or immediately following, the convergent saccade.

(K) Left: number of pre-conv assemblies identified in original data, "D" and after circular permutation of the timebase (shuffling, "S"). We estimated false discovery rate to be 19%. Right: number of pre-conv assemblies associated with spontaneous convergent saccades, "Sp".

(L) Total number of cells (left) and mean number of cells per assembly (right) for pre-conv assemblies.

(M and N) Data for peri-conv assemblies, as per (K) and (L). False discovery rate was 9%.

(legend continued on next page)

and 6M). We conclude that the vast majority of tectal assemblies we detect are associated with convergent saccades.

Larval zebrafish perform "spontaneous" convergent saccades, in the absence of an obvious visual stimulus, at very low frequency (1.89 ± 0.46/hr, mean ± SEM, range, 0–7.1/hr). However, over the full course of our imaging experiments, we collected data for a number of spontaneous convergences. This allowed us to examine whether these events were also associated with activation of OTc assemblies, which might be expected if assembly activity represents premotor activity upstream of oculomotor outputs. Using the same convergence-triggered time windows (pre-conv and peri-conv), we compared the fluorescence response of individual cells within the window to the mean signal during the remainder of the 32 s epoch. Although this analysis differs to that used for visually evoked convergences (where we could compare activity within corresponding time windows in response versus non-response trials), we found that spontaneous events were also associated with tectal assemblies (20 pre-conv assemblies and 145 peri-conv assemblies from 278 spontaneous convergences in 19 larvae; Figures 6K–6N). These assemblies had similar properties to those identified for visually evoked hunting responses. For spontaneous convergences, pre-conv assemblies increased activity in advance of convergent saccades by 1.1 s [1.1, 1.65], contained 81% [72, 100] visually responsive cells and average intra-assembly activity correlation was 0.68 [0.56, 0.82]. For spontaneous peri-conv assemblies, activity increased 0 s [0, 0.55] before eye convergence, 89% [67, 100] were visually responsive and intra-assembly activity correlation was 0.72 [0.65, 0.78] (see also Figure S5). In summary, these data suggest that assembly activation represents motor-correlated activity that is not directly, or obligately, downstream of visual input.

As expected from the design of the windows, pre-conv and peri-conv assemblies tended to overlap. We detected a greater number of assemblies with the wider peri-conv window, and the majority of convergences for which we identified a pre-conv assembly were also associated with a peri-conv assembly (77% and 85% for visually evoked and spontaneous convergences, respectively). Moreover, for visually evoked convergences, there was substantial overlap in constituent neurons: 66% of cells in pre-conv assemblies were also components of peri-conv assemblies (Figure S5).

Locations of Tectal Assemblies Are Related to Motor Parameters of Convergent Saccades

During the convergent saccade that initiates hunting routines, eye movements are often asymmetric, with the eye contralateral to the stimulus showing a greater nasal rotation on average (Figure 2D). We examined whether the laterality of tectal assemblies was associated with asymmetries in oculomotor parameters. For both pre-conv and peri-conv assemblies, the laterality of the assembly (left or right tectal location) corresponded to the eye that showed the more nasal post-saccadic position, the larger change in eye position (nasal rotation), and the greater peak eye velocity (Figures 6P and 6Q). Thus, activation of a left tectal assembly is associated with the left (ipsilateral) eye showing a larger, faster rotation and adopting a more nasal eye position than the right (contralateral) eye. This result is compatible with tectal assemblies causing asymmetric activation of extraocular motoneurons, so as to produce greater convergence of the ipsilateral eye.

The alignment of sensory and motor maps in the OTc is a characteristic of all vertebrate species studied such that activation of distinct tectal sites can produce goal-directed movements toward spatially localized sensory cues [25]. Different points along the anterior-posterior axis of the OTc correspond to different points in visual azimuth, and so we predicted that assemblies at more caudal tectal locations, corresponding to peripheral target locations, might be associated with more asymmetric convergent saccades. Specifically, vergence of the left eye should be greatest when assembly activation occurs at caudal locations in the left OTc (corresponding to the peripheral right visual field) and decline in the sequence left-caudal > left-rostral > right-rostral > right-caudal. The opposite relationship is expected for the right eye. We estimated the location of each assembly by measuring the distance of its center of mass from the posterior commissure and observed the expected trends in oculomotor parameters as a function of assembly location (Figure S7). Independent straight line fits to data for each tectum usually showed the expected positive (right eye) or negative (left eye) slope, but in the majority of cases did not achieve statistical significance. This is likely due to variation between animals producing an additional source of unexplained variance; we did not detect sufficient assemblies to assess motor mapping within individual fish. However, these trends support the possibility that assemblies conform to a motor map that directs convergent saccades toward target locations.

In summary, by imaging neural activity during behavior we were able to identify a reproducible pattern of population activity in the OTc that is associated with the release of prey-catching behavior.

DISCUSSION

Model for Initiation of Zebrafish Hunting Behavior

Figure 7 shows a working model of the neural pathway that controls the initiation of zebrafish hunting behavior. We propose that

(O) Left: example illustrating detection of onset of assembly activity. Population activity of a pre-conv assembly is shown (mean ± SD). Horizontal red line indicates threshold. The population response crosses threshold at the time indicated by a red dot, which precedes the saccade by a certain lead time (arrow). Right: histograms of lead times for pre-conv (top) and peri-conv (bottom) assemblies associated with visually evoked (left) or spontaneous (right) convergences. x axis is marked in imaging frames, relative to time of saccade (frame zero).

(P and Q) Oculomotor parameters associated with assembly activity. Post-saccadic eye position (left), change in eye position (middle), and peak nasal eye velocity (right) are compared for the eye ipsilateral to the assembly (ipsi) versus the eye contralateral to the assembly (contra). Positive values correspond to more nasal eye positions/rotations. For both types of assembly, the eye ipsilateral to the tectal assembly shows a larger post-saccadic eye position, a larger change in horizontal position and a larger peak velocity. p values obtained by paired t tests comparing ipsilateral versus contralateral eye. Data for visually evoked and spontaneous convergences are combined.

See also Figures S5–S7.

CellPress

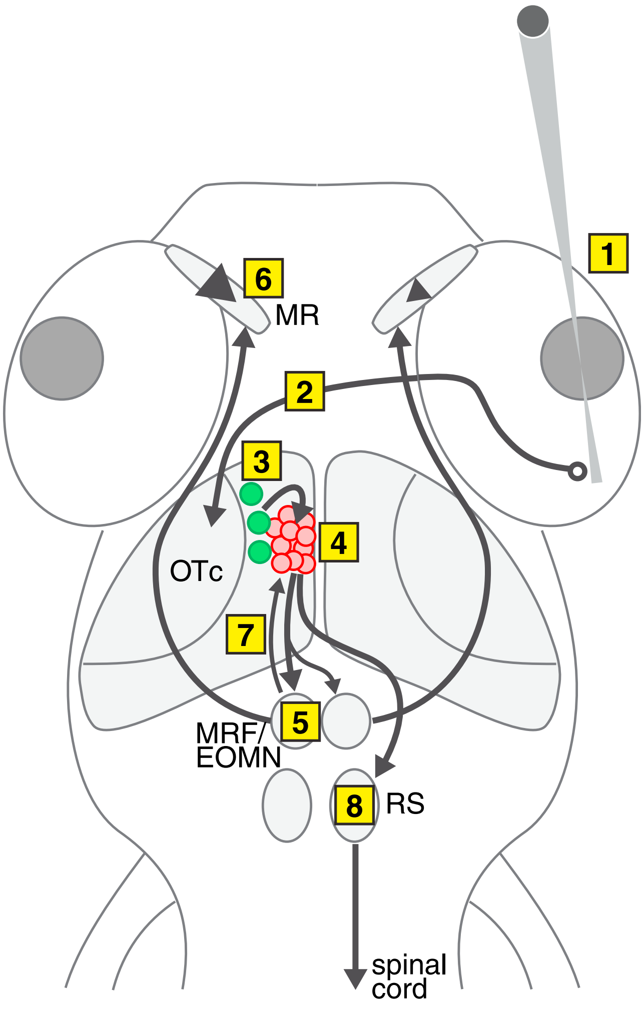

Figure 7. Model Circuit for Visual Prey Recognition and Release of Hunting Responses

(1) An image of a prey-like visual stimulus, in this case in the right visual hemifield, is cast on the right temporal retina. (2) Visual information is transmitted to retinal ganglion cell arborization fields in the diencephalon and midbrain of the contralateral (left) hemisphere. (3) Non-linear mixed selectivity neurons (green), selective for combinations of visual features that characterize optimal prey-like stimuli, are activated in the (left) rostral tectum. (4) NLMS neurons recruit the activity of tectal assemblies (red). (5) Correlated bursting of tectal assemblies activates circuits in the mesencephalic reticular formation (MRF), which control a saccadic motor program involving activation of extra-ocular medial rectus motoneurons (EOMNs) in the oculomotor nucleus. OTc efferents project to the MRF with an ipsilateral (in this case left) bias. (6) EOMNs control the ipsilateral medial rectus to produce a convergent saccade. The eye ipsilateral to the tectal assembly (Figure 6), and contralateral to the visual stimulus (Figure 2), shows the larger amplitude and velocity of eye rotation (in this case left eye). (7) Reciprocal connections from the MRF to the tectum may underlie peri-conv activity. (8) Tectal assemblies activate reticulospinal (RS) neurons, which, in turn, recruit spinal circuits to produce an orienting turn toward the visual target.

(1) hunting responses are evoked by visual objects characterized by conjunctions of visual features. Under our experimental conditions, size, speed, and contrast polarity interacted to trigger prey-catching behavior. (2) Visual information is transmitted to retinal ganglion cell arborization fields in the diencephalon and midbrain of the contralateral hemisphere [26, 27]. (3) Non-linear mixed selectivity (NLMS) neurons in the OTc function as feature-analyzing cells that mediate prey recognition. NLMS neurons may receive afferent input from retinal ganglion cells, tectal interneurons, and extra-tectal regions. (4) We propose that the activity of single, or multiple, NLMS neurons contributes to the activation of small populations of premotor tectal neurons (*assemblies*). Modulatory inputs to the tectum may also influence the recruitment of assemblies, providing a mechanism for gating the sensorimotor pathway that links prey recognition to behavioral output. Correlated bursting of a tectal assembly provides the premotor command for the release of a hunting response. Motor outputs are mediated by downstream circuits that control saccadic eye convergence and orienting turns/swims. (5) A key efferent target of premotor tectal assemblies is expected to be saccade-generating circuitry in the mesencephalic reticular formation (MRF) [28]. Tectal innervation of the MRF shows an ipsilateral bias [29], which might account for assembly activity being associated with larger, faster movements of the ipsilateral eye. (6) A circuit involving the anterior MRF and extraocular motoneurons (EOMNs) in the oculomotor nucleus, which innervate the medial rectus, would produce a convergent saccade. (7) Reciprocal projections from the MRF to the tectum [29] could underlie peri-conv assembly activity and function in feedback control of eye movements [30] or an efference copy mechanism that contributes to stable perception of prey during oculomotor and locomotor responses. (8) Tectal assemblies are also expected to establish efferent connections with reticulospinal (RS) neurons, which, in turn, control spinal cord circuits to produce goal-directed orienting turns.

Hunting Responses Are Triggered by Visual Feature Compounds

Using an assay in which prey-catching behavior can be evoked in tethered larvae using synthetic visual cues [13], we found that the features size, speed, and contrast polarity all modulated response rate, and appeared to interact. Our logistic regression model indicates that large stimuli increase the odds of a response by at least 3.8-fold compared to an otherwise identical small spot. This effect of size was unexpected because we previously showed that for freely swimming larvae, stimuli $\leq 5^\circ$ produced orienting responses, whereas those $\geq 10^\circ$ triggered aversive turns [13], which was subsequently confirmed in tethered larvae [24]. One possible explanation may relate to the absolute size and distance at which the cues are presented. In both previous studies, the screen was substantially further from the animal, and the absolute size of the aversive stimulus was 2- to 8-fold larger than the "large" spot used in this assay. Although it is not known whether larval zebrafish can evaluate absolute size of visual objects, size constancy has been reported in goldfish, including under conditions of monocular viewing [31], and tectal neurons, including those with monocular receptive fields, are sensitive to absolute object size [32]. In our assays, visual cues appear within, or move through, the binocular visual field, potentially enabling the animal to use horizontal disparity information to estimate target distance (and therefore size). Certainly after the onset of hunting, sustained eye convergence suggests larval zebrafish use a simple form of stereopsis for prey range

finding [13]. Notably, both angular sizes we tested fall within the range that can initiate natural hunting routines directed toward live prey.

Contrast polarity had the greatest effect on response rate of any of the features we tested, with dark spots increasing response odds by at least 6.5 times compared to bright spots. Furthermore, unbiased clustering identified tectal neurons that were highly selective for dark spots (and showed no detectable modulation to changes in background luminance). Many years ago, Horace Barlow suggested that retinal "off" units, concentrated in the posterior retina of the frog, are well suited to provide accurate information about the position of a fly [33], and RGCs that are responsive to small dark objects (but not bright ones) were proposed to function as "bug perceivers" [34]. In dragonflies, small target motion detector (STMD) neurons only respond to dark, negative-polarity objects [35], and it has been suggested that this selectivity is compatible with dragonflies swooping upward to capture prey that will appear dark against a bright sky. During their final capture swim, larval zebrafish also show dorsal flexion to strike their prey from below, and so selectivity for dark targets might represent an adaptive feature of the visual system for discriminating prey against a relatively bright background.

Larval zebrafish responded most strongly to moving spots that were large, dark, and fast. The interaction term in the model indicates that this conjunction of three characteristics more than doubled the odds of a response (2.5× increase). Thus, prey recognition in larval zebrafish is sensitive to feature compounds. In toads, prey-catching behavior is most effectively evoked by moving objects defined by the conjunction of multiple features, notably size, geometry, and orientation with respect to direction of motion (reviewed in [36]). This has led to the concept that computations that link visual features are central to the visual system's ability to derive the signal value of potential prey from visual input. It is worth noting that the interaction between size and speed that we observe for larval zebrafish is compatible with natural hunting responses directed toward proximal prey, which will possess similar angular size and velocity. A *Paramecium* 135 μm in size located 0.5 mm away and moving at 0.5 mm/s would appear as a 15° stimulus moving at 50°/s, in decent agreement with our large (13.2°), fast (30°/s) condition.

Visual Response Properties in the Tectum and Adjacent Regions

To examine how tectal circuits might represent visual features and feature compounds, we imaged neural activity using 2P calcium imaging and developed a clustering procedure that identified coherent groups of neurons with a range of visual tuning profiles.

Several clusters showed differing patterns of response to changes in luminance, including many cells in the habenula and torus longitudinalis (TL), in agreement with other recent imaging studies [7, 37]. In response to luminance increases, habenular cells showed sustained, excitatory responses (cluster 16) or sustained inhibition (cluster 20). In agreement with [37], clusters were lateralized, with more visually responsive neurons on the left.

In other fish species, neurons in the TL are primarily excited by dimming of the contralateral visual hemifield [38]. Surprisingly, the major visual response at the rostral pole of the TL in larval zebrafish was positive modulation after an increase in luminance. Notably, previous recordings in adult goldfish failed to detect visually evoked activity at the most rostral recording site in TL. Therefore, our observations might represent a developmental stage or species difference, or a specialized function of the anterior TL.

Visual response properties have been extensively studied in the superior colliculus/OTc, including in larval zebrafish. In agreement with previous studies [18, 19, 21, 39–42], our functional clusters exhibited direction and size selectivity. In addition, we identified neurons that preferentially responded to bright or dark spots. Although our dark spot condition included an increase in background luminance, neurons that preferred dark spots showed minimal modulation to changes in whole-field luminance and appeared to respond specifically to the dark moving spot. This suggests that neurons in the larval zebrafish tectum display contrast-polarity tuning.

By systematically varying four stimulus features, we discovered that tectal neurons show mixed selectivity to multiple features. We identified mirror-symmetric pairs of clusters with equivalent mixed feature tuning, including four clusters [9–12] that were direction selective with a net preference for large, bright spots and six direction-selective clusters [1–6] with an overall preference for large, dark stimuli. Our clustering procedure was sensitive to the timing of neural activity and consequently segregated cells on the basis of their spatial receptive fields (and anatomical locations in the retinotopic tectal map). The presence of symmetrical clusters having the same nose-tail or tail-nose tuning, but localized to left versus right tectum, implies similar perceptual sensitivity to stimuli moving in different directions in the left and right visual hemifields. This is compatible with our observations [13], and those of others [11], which show that larvae respond to live prey distributed throughout a frontally located cone of visual space. Notably, clusters 7 (right OTc) and 8 (left OTc) comprised a mirror-symmetric pair preferring large, bright spots moving nose-tail in the contralateral visual hemifield. This biased representation of stimuli moving "into" a visual hemifield is similar to observations in the posterior OTc, where a preference for tail-nose-moving spots has been reported [21]. These neurons may be involved in modulating visual processing to compensate for reafferent nose-tail motion signals produced by the animal's forward swimming movements.

Non-linear Mixed Selectivity Neurons May Underlie Visual Prey Recognition

Using a combined regression and modeling approach, we identified six groups of cells that were highly selective for the best prey-like stimuli. Their responses were better described by a non-linear model comprising interaction terms defining specific conjunctions of stimulus features as compared to a linear model with an equal number of free parameters. Therefore, we describe these highly selective cells as "non-linear mixed selectivity" (NLMS) neurons.

We suggest that NLMS neurons represent feature-analyzing cells that could underlie the ability of zebrafish to categorize visual objects as prey. How might the different types of NLMS neuron be involved in controlling hunting responses? The largest group was the non-direction-selective, non-speed-selective (nDS-nSp) type. This response property is well suited for

detection of large, dark, moving spots but fails to account for the additional preference for fast stimuli that was evident in the behavior. One possibility is that activity of multiple types of NLMS neuron is read out by downstream neurons that, in turn, trigger hunting responses. With appropriate synaptic weights, these downstream readout circuits would have a stimulus tuning profile that matches behavioral response rates. Alternatively, individual NLMS cells might be sufficient to trigger hunting responses. In this case, the overall behavioral tuning would represent the summed contribution of the NLMS population over repeated hunting episodes. Our observation of direction-selective NLMS cells would fit with this second hypothesis. These neurons could evoke hunting responses to prey moving in opposite directions, but their summed activity over time would produce a behavioral tuning profile with no net direction selectivity. Future experiments will be required to test whether the activation of NLMS cells is sufficient to trigger hunting responses and whether the different cell types evoke distinct motor outputs.

The response properties of NLMS cells indicate that the visual system performs logical operations on visual input features, but where do these computations occur? One possibility is that retinal ganglion cell (RGC) afferents already show mixed selectivity that is transmitted to post-synaptic tectal neurons. Alternatively, NLMS might be an emergent property of local tectal processing (e.g., [41]). We could not distinguish between these possibilities because we used a transgenic line in which the tectal neuropil contains a dense mix of labeled RGC axons as well as the dendritic arbors of tectal neurons. In support of the possibility that NLMS emerges in the tectum, it has long been recognized that different feature selective classes of RGCs arborize in different layers within the tectal neuropil [34] and in larval zebrafish, direction-, orientation-, and size-selective RGC axons show laminar segregation [42, 43]. Tectal periventricular neurons (PVNs) in the SPV are monopolar cells that extend their dendrites through the neuropil laminae [44] enabling them to potentially combine inputs from different feature-selective RGCs. The non-linearity inherent to the spike generation mechanism could mediate the non-linear integration of visual features that characterizes NLMS responses. Notably, ROIs in the tectal neuropil were associated with all six types of NLMS. Although this might simply correspond to the activity of tectal neuron neurites, we do not exclude the possibility that mixed selectivity is in part or whole computed in the retina.

In summary, tectal NLMS neurons represent feature detectors that are selective for the conjunction of visual features that define optimal prey-like visual objects. We suggest that NLMS cells are therefore good candidates for mediating the perceptual recognition of prey and triggering the initiation of hunting responses.

Tectal Assemblies Represent an Activity Motif Associated with Hunting Initiation

By imaging neural activity during behavior and comparing response and non-response trials, we could distinguish highly consistent visually evoked responses from activity specifically associated with the initiation of hunting.

We detected active tectal assemblies for ~10% of convergence events. This rate of detection might reflect the probability of coincidence between our imaging plane and the locus of tectal activity, although we cannot exclude the possibility that only a subset of responses are associated with active assemblies. Notwithstanding, several lines of evidence support a close relationship between assembly activity and the execution of saccadic eye convergence. (1) Assemblies with similar characteristics were detected for stimulus-evoked and spontaneous convergences. (2) The laterality and anterior-posterior location of tectal assemblies correlated with oculomotor parameters of convergent saccades. (3) We occasionally observed repeated assembly activation when the animal performed very similar saccadic responses toward the same visual stimulus, and we were imaging the same focal plane (Figure S6). (4) Our false discovery rate analysis indicated that assemblies are unlikely to be a result of background spontaneous activity.

Tectal assemblies were active immediately prior to convergent saccades (pre-conv), and in many cases population activity began more than one second before the behavior. We suggest that coordinated burst firing of these assemblies provides the premotor signal that releases hunting responses. In support of this possibility, tectal activity has a well-established role in controlling goal-directed behaviors and saccadic eye movements [45] and direct stimulation of the anterior-medial tectum in fish evokes eye convergence [46] and J-turns [22]. Projections from the OTc to the mesencephalic reticular formation (MRF) provide the efferent pathway by which tectal activity can control saccadic eye movements (reviewed in [28]). Notably, tectal loci project bilaterally to the MRF but form a greater number of synapses on the ipsilateral side [29]. This asymmetry might provide the anatomical basis for our finding that assemblies were located ipsilateral to the eye that produced the larger, faster nasal rotation.

How might the activation of tectal assemblies be controlled? Following our hypothesis that NLMS cells mediate prey recognition, we propose that they provide a key afferent input to premotor assemblies. Notably, assemblies themselves contained few, if any, NLMS cells, suggesting that perception of prey and the release of predatory responses are mediated by non-overlapping populations of tectal neurons. NLMS cells could directly or indirectly provide excitatory input onto one or more assembly neurons, with local recurrent connections within the assembly contributing to sustained and synchronized population activity. One speculative possibility is that activity in assembly networks "ramps up," or accumulates, during target viewing and triggers a behavioral response at a certain activity level corresponding to a perceptual threshold. Such a mechanism would be reminiscent of activity in cortical area LIP, where neurons show ramping activity that is thought to reflect the temporal integration of evidence during perceptual decision making [47]. Inter-hemispheric communication (for instance, via the tectal commissures) is likely to contribute to assembly recruitment because activity sometimes began while the visual cue was in the ipsilateral visual hemifield (with respect to the assembly), which is predominantly represented in the contralateral tectum. In addition to NLMS cells, other afferent inputs might modulate assembly activity (and thus response probability) in accordance with changes in internal state relating to arousal and motivation.

A significant fraction of peri-conv assemblies showed activation concurrent with, or subsequent to, convergent saccades. Although we cannot exclude the possibility of eye-movement associated activity (visual or proprioceptive sensory feedback,

which could be tested by paralyzing the eye muscles), this may represent a motor efference copy of the saccadic command, which could derive from the reciprocal connections between the OTc and MRF [29]. Efference copy signals are thought to mediate saccadic suppression (a reduction in visual acuity during saccadic eye movements), allowing retinal movement signals due to external stimuli to be distinguished from reafferent signals generated during gaze shifts [48]. Such a mechanism could contribute to accurate perception of prey during hunting by suppressing self-generated motion signals during rapid eye and body movements. In support of this, zebrafish appear to show reduced sensitivity to visual stimuli during swim bouts [24].

Conclusions

By combining functional calcium imaging with tethered virtual hunting behavior, we have functionally identified neuronal populations in the OTc that are likely key components in the sensorimotor transformations underlying a specific visually guided behavior. Our working model of the neural circuit for the initiation of hunting presents several testable hypotheses that could form the basis for future studies. In particular, determining how NLMS responses are generated and how NLMS neurons interface with premotor assemblies will be exciting challenges. High-speed volumetric imaging [23] presents the possibility to extend the analysis of neural activity from the OTc to the entire larval zebrafish brain and identify other regions that interact with and modulate core sensorimotor pathways. In addition, virtual reality hunting assays will allow circuit dynamics to be monitored during subsequent stages of hunting routines when larvae iteratively select goal-directed motor outputs to track, approach, and capture their prey.

EXPERIMENTAL PROCEDURES

Animals

Zebrafish (*Danio rerio*) larvae homozygous for both the Tg(elavl3:GCaMP5G) a4598 transgene [23] and *mitfa*$^{w2/w2}$ skin-pigmentation mutation [49] were used for all experiments. Larvae were raised in fish facility water on a 14/10-hr light/dark cycle and fed *Paramecia* from 4 days postfertilization (dpf). They were tested at 5–7 dpf. Animal handling and experimental procedures were approved by the Harvard University Standing Committee on the Use of Animals in Research and Training.

Virtual Hunting Assay

The hunting assay for tethered larval zebrafish was performed as described in [13] and the Supplemental Experimental Procedures. Visual stimuli consisted of moving spots that appeared at 100° to the left or right of the midline and then moved 200° right or left across the frontal region of visual space at constant speed along the surface of the curved screen. Bright spots were maximum contrast red stimuli (pixel value 255, Weber contrast, $C_w \sim 370$). For presentation of negative-polarity dark spots, there was first an increase in background luminance (red pixel value 25) and 2 s later a dark spot was presented (pixel value 0, $C_w = -0.97$). Whole-field light flashes were 3 s in duration at pixel values 15 (dim) or 25 (bright). Horizontal eye position was extracted at 60 Hz, and convergent saccades were detected as nasal rotations of both eyes within 150 ms of one another [13]. At each focal plane, we presented five to eight repetitions of each of the 18 visual stimuli (16 moving spots and two whole-field light-flashes) in pseudo-random order, with one stimulus presentation per 32 s “epoch.”

2P Functional Imaging

2P calcium imaging was performed using a custom-built microscope that included a 20× numerical aperture (NA) 0.95 Olympus objective and a Ti:Sapphire ultra-fast laser (Spectra-Physics MaiTai) tuned to 920 nm, with average laser power at sample of 5–10 mW. Images (500 × 500 pixels, pixel pitch 374 or 575 nm) were acquired by frame scanning at 1.8 Hz and for each larva, 10–15 focal planes were imaged with a z-spacing of 2 or 4 μm. Image acquisition, eye tracking, and visual stimulus presentation were controlled using software written in LabView and MATLAB.

Data Analysis

Data analysis was performed using scripts written in MATLAB as described in the Supplemental Experimental Procedures. For all statistical tests, two-tailed p values are reported.

SUPPLEMENTAL INFORMATION

Supplemental Information includes Supplemental Experimental Procedures, seven figures, and one table and can be found with this article online at http://dx.doi.org/10.1016/j.cub.2015.01.042.

AUTHOR CONTRIBUTIONS

I.H.B. performed the experiments and analyzed the data. I.H.B. and F.E. conceived the project, interpreted the results, and wrote the manuscript.

ACKNOWLEDGMENTS

The authors thank Martin Haesemeyer, Adam Kampff, Timothy Dunn, and the reviewers for helpful discussions and critical reading of the manuscript and Polina Kehayova for invaluable encouragement. I.H.B. was supported by a Sir Henry Wellcome Postdoctoral Fellowship from the Wellcome Trust. F.E. was supported by NIH grants 1R01DA030304-01 and DP1-NS082121-02.

Received: November 25, 2014
Revised: January 18, 2015
Accepted: January 19, 2015
Published: March 5, 2015

REFERENCES

1. Tinbergen, N. (1953). The Herring Gull’s World. (London: Collins).
2. Ingle, D., and Crews, D. (1985). Vertebrate neuroethology: definitions and paradigms. Annu. Rev. Neurosci. *8*, 457–494.
3. Laming, P.R., and Cairns, C. (1998). Effects of food, glucose, and water ingestion on feeding activity in the toad (Bufo bufo). Behav. Neurosci. *112*, 1266–1272.
4. Brzoska, J., and Schneider, H. (1978). Modification of prey-catching behavior by learning in the common toad (Bufo b. bufo [L], Anura, Amphibia): Changes in responses to visual objects and effects of auditory stimuli. Behav. Processes *3*, 125–136.
5. O’Malley, D.M., Kao, Y.H., and Fetcho, J.R. (1996). Imaging the functional organization of zebrafish hindbrain segments during escape behaviors. Neuron *17*, 1145–1155.
6. Ahrens, M.B., Li, J.M., Orger, M.B., Robson, D.N., Schier, A.F., Engert, F., and Portugues, R. (2012). Brain-wide neuronal dynamics during motor adaptation in zebrafish. Nature *485*, 471–477.
7. Portugues, R., Feierstein, C.E., Engert, F., and Orger, M.B. (2014). Whole-brain activity maps reveal stereotyped, distributed networks for visuomotor behavior. Neuron *81*, 1328–1343.
8. Borla, M.A., Palecek, B., Budick, S., and O’Malley, D.M. (2002). Prey capture by larval zebrafish: evidence for fine axial motor control. Brain Behav. Evol. *60*, 207–229.
9. McElligott, M.B., and O’malley, D.M. (2005). Prey tracking by larval zebrafish: axial kinematics and visual control. Brain Behav. Evol. *66*, 177–196.
10. Gahtan, E., Tanger, P., and Baier, H. (2005). Visual prey capture in larval zebrafish is controlled by identified reticulospinal neurons downstream of the tectum. J. Neurosci. *25*, 9294–9303.

11. Patterson, B.W., Abraham, A.O., MacIver, M.A., and McLean, D.L. (2013). Visually guided gradation of prey capture movements in larval zebrafish. J. Exp. Biol. *216*, 3071–3083.

12. McClenahan, P., Troup, M., and Scott, E.K. (2012). Fin-tail coordination during escape and predatory behavior in larval zebrafish. PLoS ONE *7*, e32295.

13. Bianco, I.H., Kampff, A.R., and Engert, F. (2011). Prey capture behavior evoked by simple visual stimuli in larval zebrafish. Front. Syst. Neurosci. *5*, 101.

14. Stuermer, C.A. (1988). Retinotopic organization of the developing retinotectal projection in the zebrafish embryo. J. Neurosci. *8*, 4513–4530.

15. Torres, B., Luque, M.A., Pérez-Pérez, M.P., and Herrero, L. (2005). Visual orienting response in goldfish: a multidisciplinary study. Brain Res. Bull. *66*, 376–380.

16. Klier, E.M., Wang, H., and Crawford, J.D. (2001). The superior colliculus encodes gaze commands in retinal coordinates. Nat. Neurosci. *4*, 627–632.

17. Chen, Q., Deng, H., Brauth, S.E., Ding, L., and Tang, Y. (2012). Reduced performance of prey targeting in pit vipers with contralaterally occluded infrared and visual senses. PLoS ONE *7*, e34989.

18. Muto, A., Ohkura, M., Abe, G., Nakai, J., and Kawakami, K. (2013). Real-time visualization of neuronal activity during perception. Curr. Biol. *23*, 307–311.

19. Del Bene, F., Wyart, C., Robles, E., Tran, A., Looger, L., Scott, E.K., Isacoff, E.Y., and Baier, H. (2010). Filtering of visual information in the tectum by an identified neural circuit. Science *330*, 669–673.

20. Smear, M.C., Tao, H.W., Staub, W., Orger, M.B., Gosse, N.J., Liu, Y., Takahashi, K., Poo, M.M., and Baier, H. (2007). Vesicular glutamate transport at a central synapse limits the acuity of visual perception in zebrafish. Neuron *53*, 65–77.

21. Niell, C.M., and Smith, S.J. (2005). Functional imaging reveals rapid development of visual response properties in the zebrafish tectum. Neuron *45*, 941–951.

22. Fajardo, O., Zhu, P., and Friedrich, R.W. (2013). Control of a specific motor program by a small brain area in zebrafish. Front Neural Circuits *7*, 67.

23. Ahrens, M.B., Orger, M.B., Robson, D.N., Li, J.M., and Keller, P.J. (2013). Whole-brain functional imaging at cellular resolution using light-sheet microscopy. Nat. Methods *10*, 413–420.

24. Trivedi, C.A., and Bollmann, J.H. (2013). Visually driven chaining of elementary swim patterns into a goal-directed motor sequence: a virtual reality study of zebrafish prey capture. Front Neural Circuits *7*, 86.

25. Masino, T. (1992). Brainstem control of orienting movements: intrinsic coordinate systems and underlying circuitry. Brain Behav. Evol. *40*, 98–111.

26. Burrill, J.D., and Easter, S.S., Jr. (1994). Development of the retinofugal projections in the embryonic and larval zebrafish (Brachydanio rerio). J. Comp. Neurol. *346*, 583–600.

27. Robles, E., Laurell, E., and Baier, H. (2014). The retinal projectome reveals brain-area-specific visual representations generated by ganglion cell diversity. Curr. Biol. *24*, 2085–2096.

28. Angeles Luque, M., Pilar Pérez-Pérez, M., Herrero, L., and Torres, B. (2005). Involvement of the optic tectum and mesencephalic reticular formation in the generation of saccadic eye movements in goldfish. Brain Res. Brain Res. Rev. *49*, 388–397.

29. Pérez-Pérez, M.P., Luque, M.A., Herrero, L., Nunez-Abades, P.A., and Torres, B. (2003). Connectivity of the goldfish optic tectum with the mesencephalic and rhombencephalic reticular formation. Exp. Brain Res. *151*, 123–135.

30. Soetedjo, R., Kaneko, C.R.S., and Fuchs, A.F. (2002). Evidence that the superior colliculus participates in the feedback control of saccadic eye movements. J. Neurophysiol. *87*, 679–695.

31. Douglas, R.H., Eva, J., and Guttridge, N. (1988). Size constancy in goldfish (Carassius auratus). Behav. Brain Res. *30*, 37–42.

32. Spreckelsen, C., Schürg-Pfeiffer, E., and Ewert, J.P. (1995). Responses of retinal and tectal neurons in non-paralyzed toads Bufo bufo and B. marinus to the real size versus angular size of objects moved at variable distance. Neurosci. Lett. *184*, 105–108.

33. Barlow, H.B. (1953). Summation and inhibition in the frog's retina. J. Physiol. *119*, 69–88.

34. Lettvin, J.Y., Maturana, H.R., McCulloch, W.S., and Pitts, W.H. (1959). What the frog's eye tells the frog's brain. Proc. Inst. Radio Engr. *47*, 1940–1951.

35. Wiederman, S.D., Shoemaker, P.A., and O'Carroll, D.C. (2013). Correlation between OFF and ON channels underlies dark target selectivity in an insect visual system. J. Neurosci. *33*, 13225–13232.

36. Ewert, J.P. (1997). Neural correlates of key stimulus and releasing mechanism: a case study and two concepts. Trends Neurosci. *20*, 332–339.

37. Dreosti, E., Vendrell Llopis, N., Carl, M., Yaksi, E., and Wilson, S.W. (2014). Left-right asymmetry is required for the habenulae to respond to both visual and olfactory stimuli. Curr. Biol. *24*, 440–445.

38. Northmore, D.P. (1984). Visual and saccadic activity in the goldfish torus longitudinalis. J. Comp. Physiol. A *155*, 333–340.

39. Grama, A., and Engert, F. (2012). Direction selectivity in the larval zebrafish tectum is mediated by asymmetric inhibition. Front. Neural Circuits *6*, 59.

40. Gabriel, J.P., Trivedi, C.A., Maurer, C.M., Ryu, S., and Bollmann, J.H. (2012). Layer-specific targeting of direction-selective neurons in the zebrafish optic tectum. Neuron *76*, 1147–1160.

41. Hunter, P.R., Lowe, A.S., Thompson, I.D., and Meyer, M.P. (2013). Emergent properties of the optic tectum revealed by population analysis of direction and orientation selectivity. J. Neurosci. *33*, 13940–13945.

42. Preuss, S.J., Trivedi, C.A., vom Berg-Maurer, C.M., Ryu, S., and Bollmann, J.H. (2014). Classification of object size in retinotectal microcircuits. Curr. Biol. *24*, 2376–2385.

43. Nikolaou, N., Lowe, A.S., Walker, A.S., Abbas, F., Hunter, P.R., Thompson, I.D., and Meyer, M.P. (2012). Parametric functional maps of visual inputs to the tectum. Neuron *76*, 317–324.

44. Meek, J., and Schellart, N.A. (1978). A Golgi study of goldfish optic tectum. J. Comp. Neurol. *182*, 89–122.

45. Sparks, D.L. (2002). The brainstem control of saccadic eye movements. Nat. Rev. Neurosci. *3*, 952–964.

46. Salas, C., Herrero, L., Rodriguez, F., and Torres, B. (1997). Tectal codification of eye movements in goldfish studied by electrical microstimulation. f. Neuroscience *78*, 271–288.

47. Huk, A.C., and Shadlen, M.N. (2005). Neural activity in macaque parietal cortex reflects temporal integration of visual motion signals during perceptual decision making. J. Neurosci. *25*, 10420–10436.

48. Johnstone, J.R., and Mark, R.F. (1969). Evidence for efference copy for eye movements in fish. Comp. Biochem. Physiol. *30*, 931–939.

49. Lister, J.A., Robertson, C.P., Lepage, T., Johnson, S.L., and Raible, D.W. (1999). nacre encodes a zebrafish microphthalmia-related protein that regulates neural-crest-derived pigment cell fate. Development *126*, 3757–3767.

Current Biology
Supplemental Information

Visuomotor Transformations Underlying Hunting Behavior in Zebrafish

Isaac H. Bianco and Florian Engert

FIGURE S1

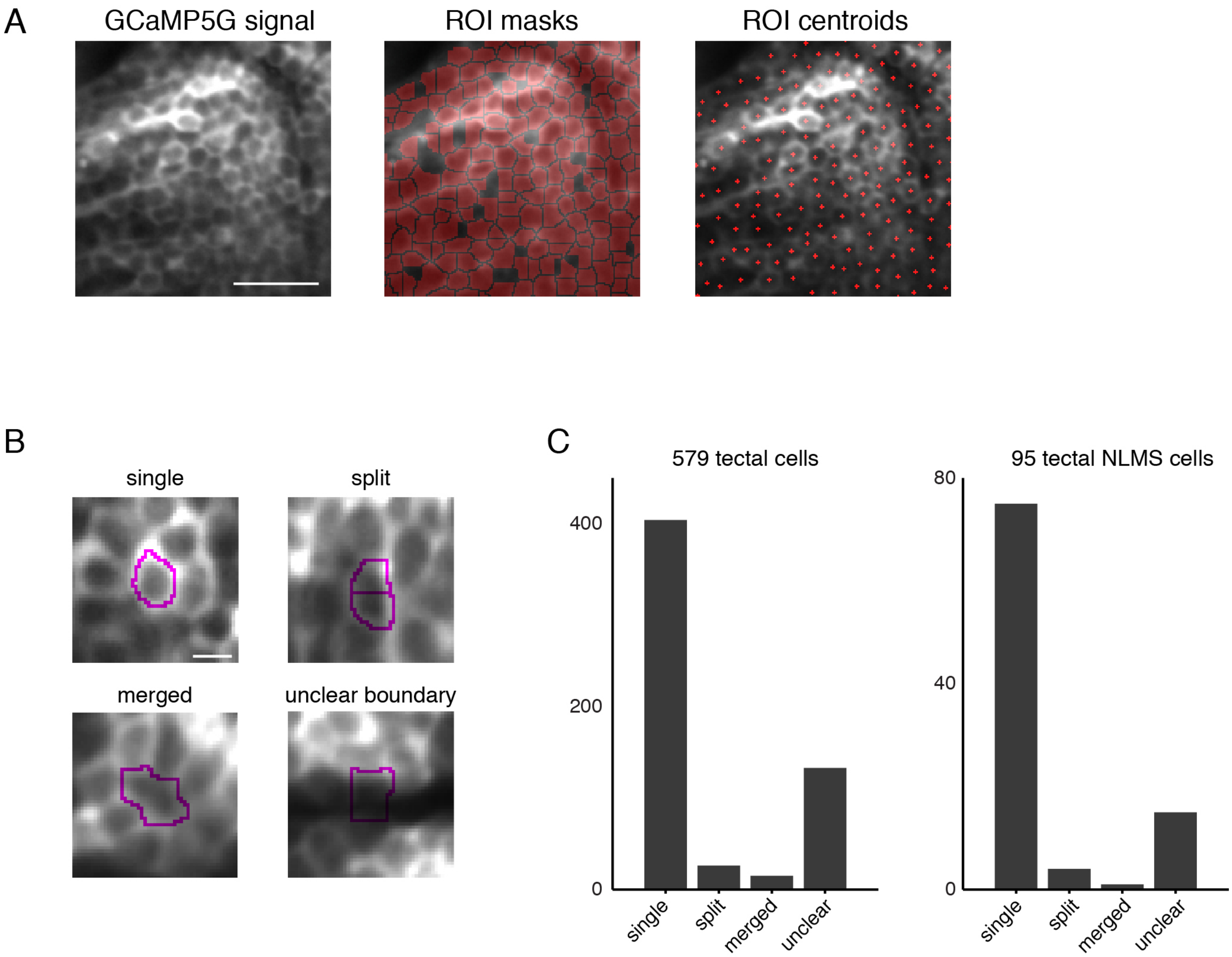

A
GCaMP5G signal
ROI masks
ROI centroids
B
single
split
merged
unclear boundary
C
579 tectal cells
95 tectal NLMS cells
400
200
0
80
40
0
single
split
merged
unclear

Figure S1 : Automatic identification of ROIs (related to Figure 3).

(A) Example of automated segmentation. Left panel shows 'anatomical' image obtained by taking the mean time-series projection of all motion-corrected images from a focal plane. Field-of-view shows a region of the anterior left OTc (SPV layer). Scale bar: 20 μm. Automatic segmentation was performed using a watershed-based algorithm [S1]. ROI masks (middle panel) or centroids (right panel) are shown overlaid on the anatomical image. The 'holes' in the ROI masks correspond to ROIs that were rejected based on morphological criteria (see Experimental Procedures). ROIs corresponded well to individual somata and similar sized ROIs were detected in neuropil regions (not visible in this field-of-view). We refer to the former as cells, although it is possible these ROIs may include neurites of other neurons within the densely packed SPV of the OTc. For this fish, the average area of all ROIs was 15.9 μm^2 [11.5–20.7] (median, interquartile range). For ROIs assigned to cells body regions (i.e. excluding neuropil ROIs), average area was 12.7 μm^2 [6.4–19], equivalent to a circle of diameter 4.0 μm [2.9–4.9]. (B,C) We estimated error rates for the automated segmentation procedure by manually inspecting 579 randomly selected ROIs and classifying them as 'single', 'split', 'merged' or 'unclear boundary'. (B) Examples of ROIs for each category. 'Single' are ROIs that clearly define a single cell soma. 'Split' ROIs correspond to part of a cell but where one or more additional ROIs are localized to other region(s) of the same cell. 'Merged' ROIs erroneously encompass more than one cell. 'Unclear boundary' ROIs did not show the typical 'doughnut-shaped' GCaMP signal localized to the cytoplasm/membrane surrounding a dark nucleus. These sometimes occurred at tissue boundaries (e.g. the midline as shown here) or corresponded to neurites. (C) Numbers of ROIs assigned to each category. Left panel: complete set of 579 ROIs. Right panel: 95 tectal NLMS cells, which were part of the random sample. Error rates for NLMS cells are comparable to the total sample.

FIGURE S2

A

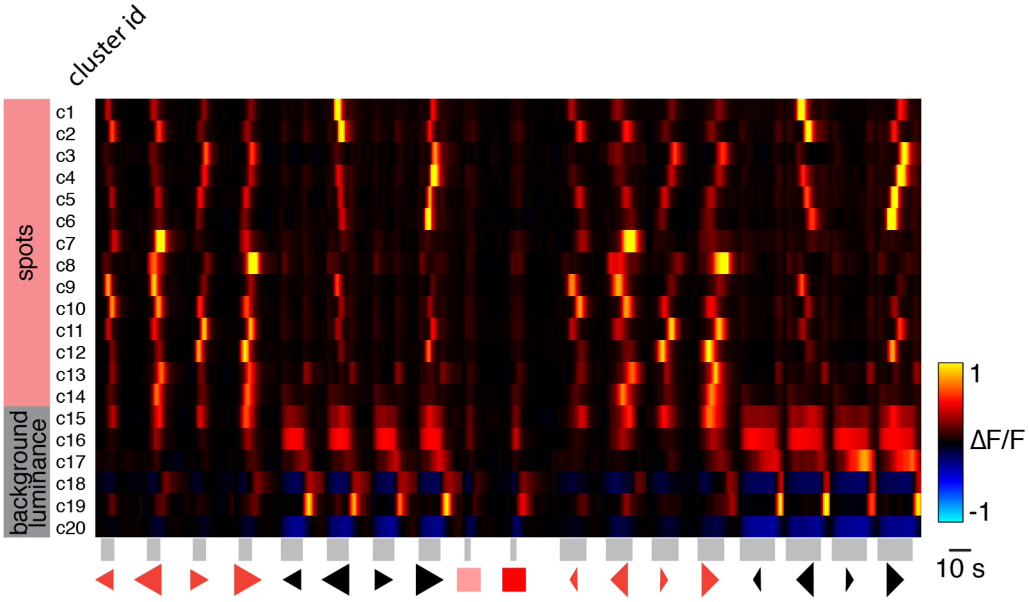
cluster id
spots
background luminance
c1
c2
c3
c4
c5
c6
c7
c8
c9
c10
c11
c12
c13
c14
c15
c16
c17
c18
c19
c20
1
ΔF/F
-1
10 s

B

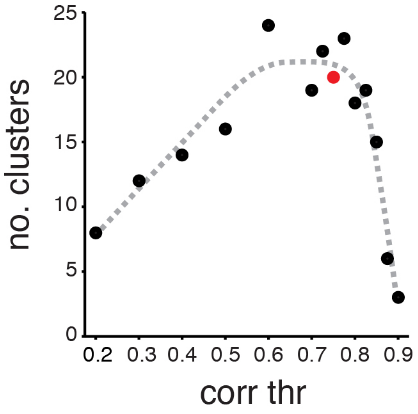
no. clusters
25
20
15
10
5
0
0.2
0.3
0.4
0.5
0.6
0.7
0.8
0.9
corr thr

Figure S2 : Additional cluster details (related to Figure 3).

(A) Heat-map representation of centroids of the 20 clusters of visually responsive neurons shown in Figure 3B. Centroids are shown as mean $\Delta F/F$ of all cells within each cluster. Gray bars and symbols indicate visual stimulus presentation periods. (B) Number of clusters identified by our clustering procedure as a function of correlation threshold. We chose a correlation threshold of 0.75, which produced 20 clusters (red datapoint, see also Experimental Procedures).

FIGURE S3

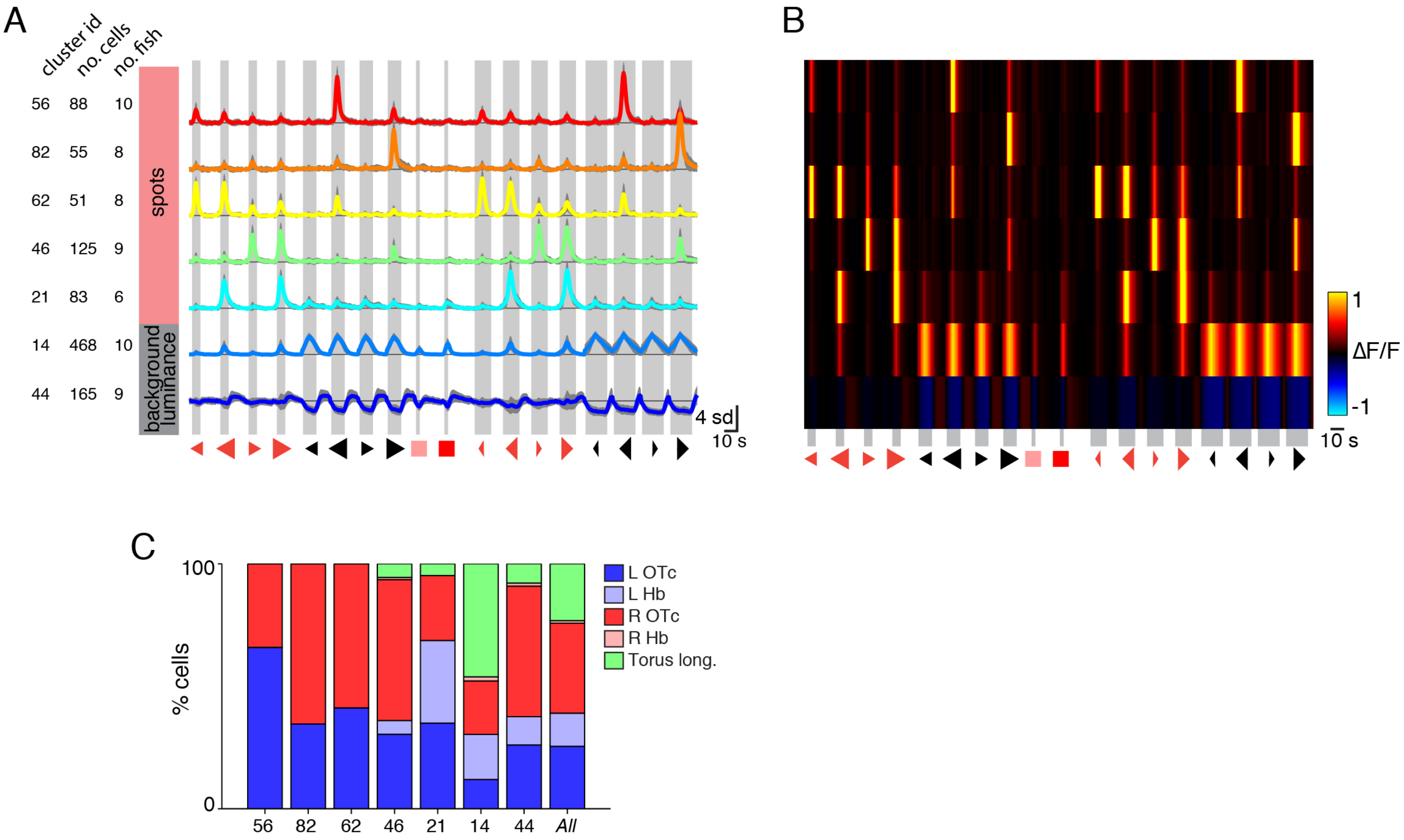
A
cluster id
no. cells
no. fish
56 88 10
82 55 8
62 51 8
46 125 9
21 83 6
14 468 10
44 165 9
spots
background luminance
4 sd
10 s
B
1
ΔF/F
-1
10 s
C
100
0
% cells
56
82
62
46
21
14
44
All
L OTc
L Hb
R OTc
R Hb
Torus long.

Figure S3 : Clustering using Gaussian Mixture Modeling (related to Figure 3).

(A,B) Neurons from 10 fish were clustered using a method based on Gaussian mixture modeling. The centroids of the 7 clusters that were identified are shown in (A) and alternatively as a heat-map representation in (B). Shading in (A) represents standard deviation across cells. Note that prior to clustering, the peak responses of all cells were aligned for each stimulus, such that they appear to respond maximally when the visual cues are at 0°. Consequently, cells are not segregated based on differential timing of their fluorescence responses and so fewer clusters are produced as compared to Figure 3B. However, clusters with comparable visual feature tuning are detected. Shaded bars indicate visual stimulus presentation. (C) Anatomical locations of the cells associated with each cluster.

FIGURE S4

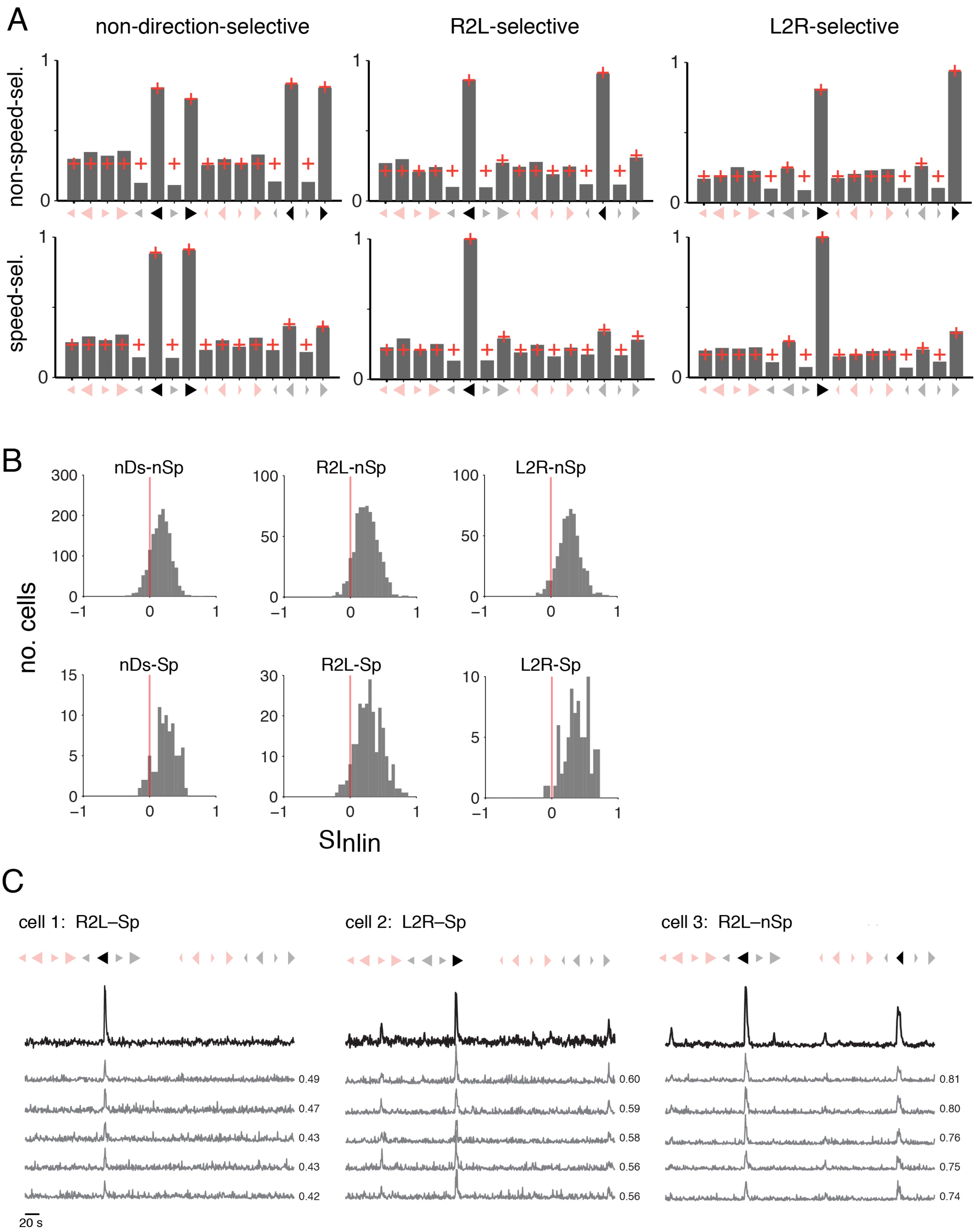
A
non-direction-selective
R2L-selective
L2R-selective
non-speed-sel.
speed-sel.
1
0
B
nDs-nSp
R2L-nSp
L2R-nSp
nDs-Sp
R2L-Sp
L2R-Sp
no. cells
SInlin
300
200
100
50
15
10
5
30
20
–1
0
1
C
cell 1: R2L–Sp
cell 2: L2R–Sp
cell 3: R2L–nSp
0.49
0.47
0.43
0.43
0.42
0.60
0.59
0.58
0.56
0.56
0.81
0.80
0.76
0.75
0.74
20 s

Figure S4 : Non-linear model fits and single NLMS voxels (related to Figure 5).

(A) Comparison of observed stimulus responses to those predicted by the non-linear model in Figure 5B. Gray bars indicate mean normalized responses and red crosses indicate mean normalized responses predicted by the model. Model predictions were computed for each ROI using the fitted model coefficients. To facilitate comparison of tuning profiles between ROIs, responses were normalized to the maximal stimulus response. (B) Non-linear selectivity index (SI_{nlin}) for ROIs associated with each regressor. (C) Responses of single voxels from NLMS ROIs. Three examples are shown of NLMS ROIs corresponding to neuronal somata in the optic tectum. Schematics indicate the regressor with which each ROI is associated. Top traces show the visual response vectors (VRVs) for the ROI (average of all component pixels, black) and lower traces show VRVs for single voxels belonging to the ROI (gray). Numbers on the right indicate the Pearson's correlation coefficient between the voxel VRV and ROI VRV. Although traces for single voxels show reduced signal-to-noise, they clearly display similar stimulus selectivity to the parent ROI. All traces shown as unfiltered raw pixel values (arbitrary units).

FIGURE S5

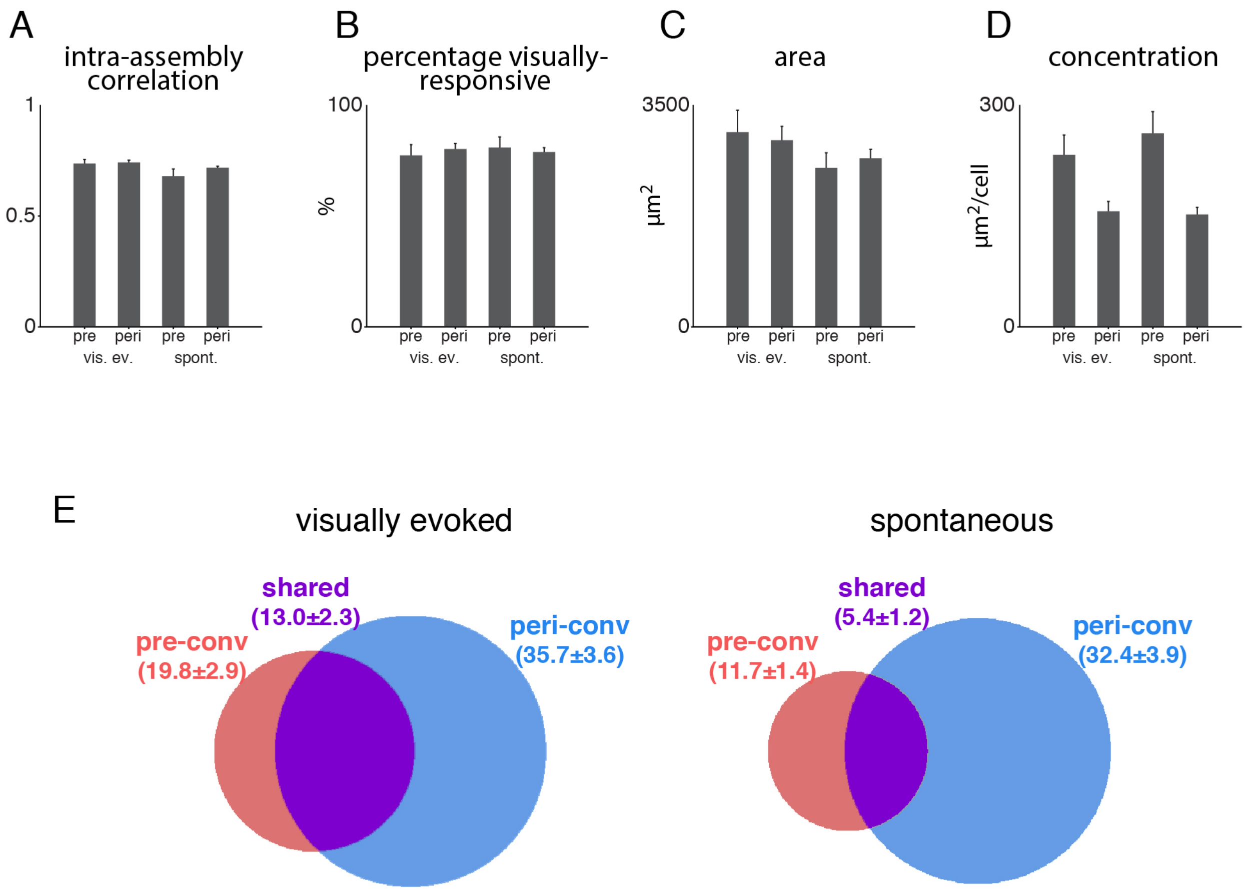

A
intra-assembly correlation
1
0.5
0
pre
peri
pre
peri
vis. ev.
spont.
B
percentage visually-responsive
100
%
0
C
area
3500
μm2
0
D
concentration
300
μm2/cell
0
E
visually evoked
shared
(13.0±2.3)
pre-conv
(19.8±2.9)
peri-conv
(35.7±3.6)
spontaneous
shared
(5.4±1.2)
pre-conv
(11.7±1.4)
peri-conv
(32.4±3.9)

Figure S5 : Additional characteristics of tectal cell assemblies (related to Figure 6).

(A) Intra-assembly correlation: Mean correlation of activity of each cell in an assembly with the mean activity of the assembly. (B) Percentage visually responsive cells: Percentage of cells in the assembly classified as responsive to at least one of the 18 visual stimuli. (C) Area: Area of ellipse fit to the spatial distribution of response-modulated cells. (D) Concentration: Area of ellipse divided by number of cells enclosed by ellipse (number of cells in assembly). (E) Venn diagram showing overlap between pre-conv and peri-conv assemblies. Numbers in parentheses indicate cells belonging to each type of assembly and shared by both assemblies. Data presented as mean $\pm$ sem. Abbreviations: pre, pre-conv assembly; peri, peri-conv assembly; vis. ev., visually evoked convergence; spont., spontaneous convergence.

FIGURE S6

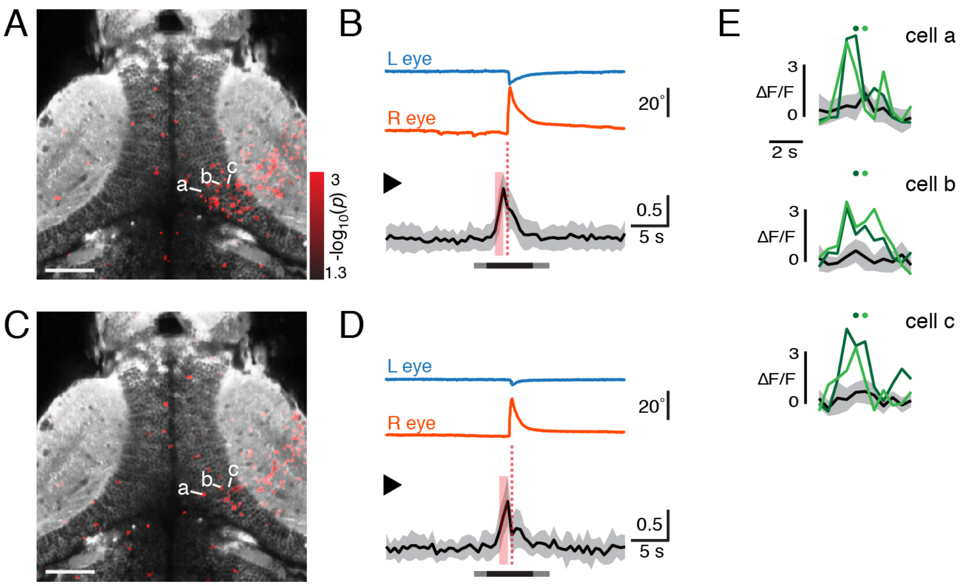
A
B
L eye
R eye
20°
0.5
5 s
C
D
E
cell a
cell b
cell c
ΔF/F
2 s
3
1.3
-log10(p)
a
b
c

Figure S6 : Repeated activation of a tectal assembly (related to Figure 6).

Example of two pre-conv assemblies that were detected in overlapping anatomical locations in the right OTc and associated with highly similar behavioral responses. (A,B) First pre-conv assembly (same example as Figure 6G,H). (A) Response-modulated ROIs (red) overlaid on an anatomical projection image (gray). Scale bar: 50 μm. (B) Top: Eye position traces. Bottom: Normalized activity of all cells within the assembly (mean $\pm$ sd). Notice that the right eye shows the greater nasal rotation and the assembly is located in the right OTc. (C,D) Second pre-conv assembly that was active approximately 1.5 min later in association with another prey-catching response. Notice that the larva performed a similar saccadic response to the same type of visual stimulus (large, dark, fast, left–right moving spot) and the assemblies occupy overlapping anatomical locations in the right OTc. (E) Three examples of response-modulated cells that were common to both assemblies (locations marked in (A,C)). Responses for the first and second convergence events are shown in dark and light green respectively. Dots above the traces indicate the imaging frame during which the convergent saccade occurred. Activity in non-response trials, for the same visual stimulus, is shown in gray (mean $\pm$ sd).

FIGURE S7

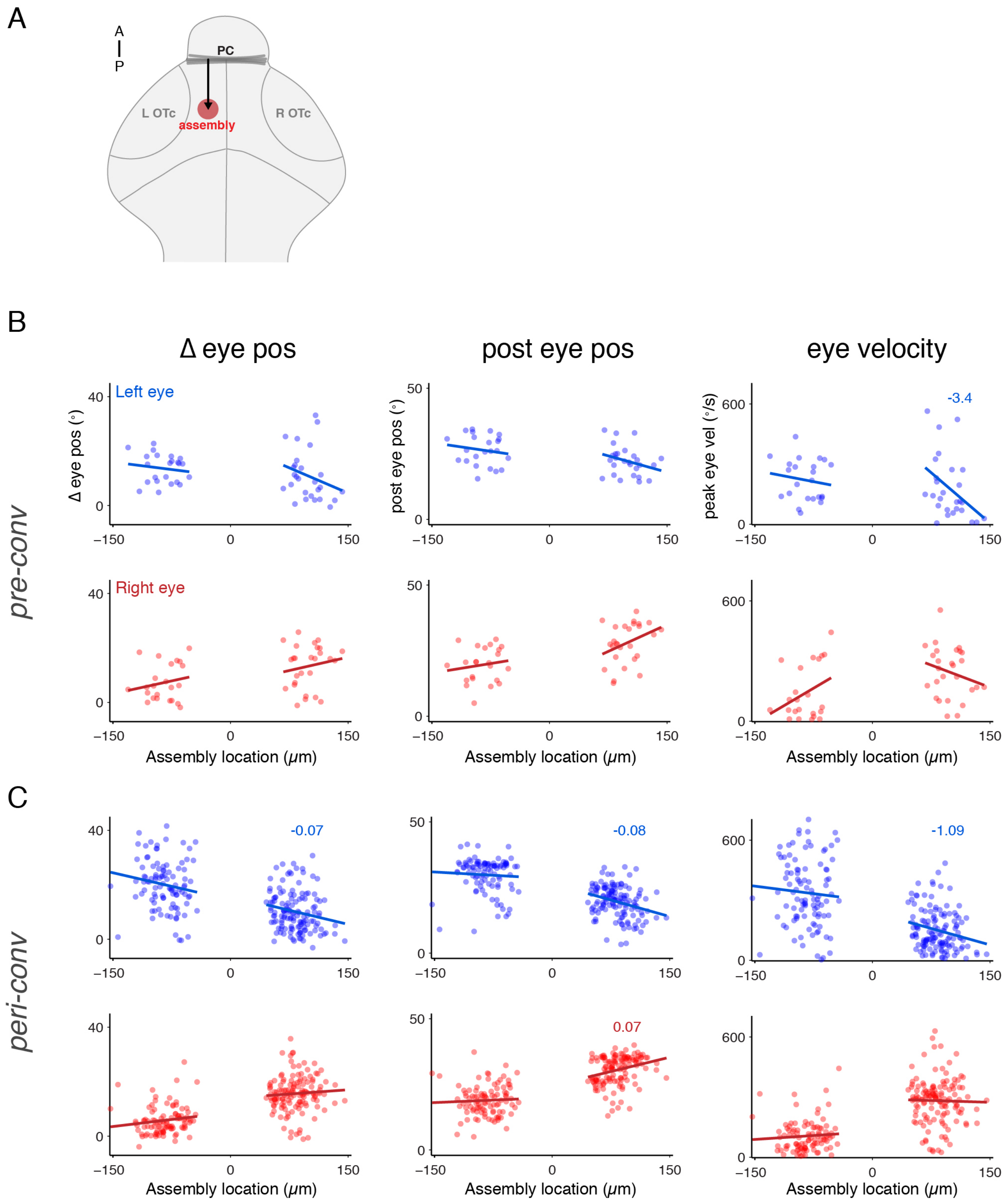

A
A
P
PC
L OTc
R OTc
assembly
B
Δ eye pos
post eye pos
eye velocity
Left eye
Right eye
Δ eye pos (°)
post eye pos (°)
peak eye vel (°/s)
-3.4
pre-conv
Assembly location (μm)
C
-0.07
-0.08
-1.09
0.07
peri-conv
Assembly location (μm)

Figure S7 : Comparison of oculomotor responses to anatomical location of tectal assemblies (related to Figure 6).

(A) Schematic illustrating how assembly location was measured. The distance from the center of the posterior commissure (PC) to the center-of-mass of the assembly was measured parallel to the anterior-posterior axis. The locations of left tectal assemblies are given as negative values (more negative values correspond to more caudal locations) whereas locations in the right tectum are given as positive values (greater values represent more caudal locations). (B) Comparison of oculomotor responses to locations of pre-conv assemblies. Upper row represents left eye, lower row is right eye. Left panels show change in eye position during convergent saccade, middle panels show eye position immediately following the convergent saccade and right panels show peak eye velocity during the saccade. For all parameters, positive values correspond to nasalward position or velocity. Lines indicate independent straight line fits for left or right tectal assemblies. Where the 95% confidence interval for the gradient of the line did not overlap with zero, the fit value of the gradient is shown. (C) Data for peri-conv assemblies, as per (B).

Table S1 : Details of visually responsive clusters

Cluster id	No. cells	No. fish	*r* mean ± sd	Type	SI Direction mean ± sd	SI Size mean ± sd	SI Speed mean ± sd	SI Polarity mean ± sd	Notes
1	216	11	0.78 ± 0.06	Spot	-0.27 ± 0.21	0.47 ± 0.12	-0.03 ± 0.12	0.23 ± 0.18	Tail–nose selective, L OTc
2	267	13	0.76 ± 0.07	Spot	-0.32 ± 0.26	0.38 ± 0.13	0.01 ± 0.12	0.05 ± 0.21	Nose–tail selective, R OTc
3	31	9	0.81 ± 0.04	Spot	0.48 ± 0.11	0.30 ± 0.11	-0.05 ± 0.07	-0.11 ± 0.13	Nose–tail selective, L OTc
4	135	9	0.77 ± 0.06	Spot	0.31 ± 0.21	0.45 ± 0.12	-0.04 ± 0.16	0.23 ± 0.16	Nose–tail selective, L OTc
5	74	8	0.79 ± 0.03	Spot	0.18 ± 0.13	0.36 ± 0.11	-0.12 ± 0.13	0.06 ± 0.15	Tail–nose selective, R OTc
6	71	9	0.79 ± 0.06	Spot	0.31 ± 0.19	0.55 ± 0.12	-0.11 ± 0.11	0.36 ± 0.16	Tail–nose selective, R OTc
7	108	11	0.77 ± 0.05	Spot	-0.37 ± 0.14	0.43 ± 0.13	0.01 ± 0.17	-0.49 ± 0.13	Nose–tail selective, R OTc
8	122	12	0.77 ± 0.06	Spot	0.24 ± 0.20	0.45 ± 0.12	-0.03 ± 0.14	-0.46 ± 0.16	Nose–tail selective, L OTc
9	215	12	0.75 ± 0.09	Spot	-0.45 ± 0.18	0.26 ± 0.19	-0.03 ± 0.15	-0.38 ± 0.18	Tail–nose selective, L OTc
10	429	13	0.76 ± 0.07	Spot	-0.13 ± 0.22	0.15 ± 0.14	0.00 ± 0.12	-0.47 ± 0.16	Nose–tail selective, R OTc
11	274	12	0.76 ± 0.06	Spot	0.29 ± 0.24	0.20 ± 0.16	-0.03 ± 0.09	-0.35 ± 0.21	Nose–tail selective, L OTc
12	244	13	0.78 ± 0.07	Spot	0.48 ± 0.18	0.26 ± 0.14	-0.05 ± 0.11	-0.32 ± 0.19	Tail–nose selective, R OTc
13	40	6	0.77 ± 0.06	Spot	0.03 ± 0.12	0.38 ± 0.14	-0.12 ± 0.11	-0.56 ± 0.12	Non-direction selective
14	701	14	0.71 ± 0.08	Spot	0.03 ± 0.17	0.45 ± 0.18	-0.06 ± 0.12	-0.48 ± 0.19	Non-direction selective
15	45	6	0.77 ± 0.05	Lum					Responsive to all stimuli
16	1839	14	0.77 ± 0.11	Lum					Positive modulation to luminance increase
17	17	7	0.81 ± 0.04	Lum					Slow positive modulation to luminance increase
18	126	10	0.82 ± 0.07	Lum					Negative modulation to luminance increase & positive modulation to luminance decrease
19	52	9	0.79 ± 0.06	Lum					Positive modulation to luminance decrease
20	86	9	0.78 ± 0.06	Lum					Negative modulation to luminance increase

Notes:

1. *r* : Pearson's correlation coefficient between each cell's visual response vector and the cluster centroid

2. Selectivity Indices (SI):
   Direction : Rightwards, positive; Leftwards, negative
   Size : Large, positive; Small, negative.
   Speed : Fast, positive; Slow, negative.
   Polarity : Dark, positive; Bright, negative.

3. Abbreviations:
   Spot : moving spot responsive
   Lum : background luminance responsive
   L : left
   R : right
   OTc : optic tectum

Supplemental Experimental Procedures

Virtual hunting assay

The virtual hunting assay was similar to that described in [S2]. Larval zebrafish were mounted in 2% low-melting temperature agarose (Invitrogen), dissolved in fish-facility water, in a 35 mm petri-dish lid. Once the agarose had gelled, an opthalmic scalpel was used to carefully remove the agarose anterior to the otic vesicle and caudal to the swim bladder. Consequently the animal was held around the head and body but was able to freely move its eyes and tail. A strip of diffusive filter (3026, Rosco Inc, CA) was fixed to the outside wall of the chamber and served as a screen. The mid-point between the fish's eyes was 7 mm from the screen. Visual stimuli were designed in Matlab (Mathworks, MA) using the Psychophysics toolbox [S3] and back-projected onto the screen using a laser pico-projector (SHOWWX, Microvision). A colored Wratten filter (no. 25, Kodak) was placed in front of the projector to minimize interference with GCaMP imaging; we detected no bleed-through of the visual stimulus to our imaging data. To monitor eye movements, a 720 nm LED was used to illuminate the larva from below through a diffuser and the eyes were imaged at 60 Hz using a charge-coupled device camera (Guppy F-033, Allied Vision Technology). Eye-tracking was performed using custom scripts written in LabView (National Instruments). Horizontal eye position was defined as the angle between the long axis of an ellipse fit to the eye and a line parallel to the midline of the head and convergent saccades were detected using custom scripts written in Matlab.

2P functional imaging

The resolution of the microscope was estimated as 370 nm laterally and 1.44 μm axially (FWHM) as per [S4].

Data analysis

We used logistic regression to model response rate as a function of four binary-coded stimulus features. To identify the model that best described the data, we used stepwise regression, which iteratively adds or removes model terms to optimize the model fit. Depending on starting conditions, the stepwise regression algorithm converged on one of the following two models:

Model A:

$$ln\left(\frac{R}{1-R}\right) = \beta_0 + \beta_1[Sz] + \beta_2[Pol] + \beta_3[Sp \cdot Sz]$$

Model B:

$$ln\left(\frac{R}{1-R}\right) = \beta_0 + \beta_1[Sz] + \beta_2[Pol] + \beta_3[Sp \cdot Sz \cdot Pol]$$

where R is response rate, $R/(1-R)$ is response odds and Dir, Sz, Sp and Pol are binary variables describing the features direction, size, speed and contrast-polarity with the coding scheme summarized in Figure 2E. As a principled means to compare these models we used cross-validation to evaluate model accuracy. We randomly segregated our dataset into two equal parts to produce a training and a test dataset. Both models were fit using the training dataset and the model predictions were then evaluated against the unseen test dataset by computing the proportion of the variance of the test dataset explained by the model (R^2). Model B had a slightly greater cross-validated $R^2 = 0.82$ compared to Model A, $R^2 = 0.79$. In Figure 2 we show the response rates predicted by Model B and the estimated model coefficients.

To correct for slow specimen drift and small movements of the animal, individual imaging frames were aligned to the mean image for that z-plane with sub-pixel precision [S5]. The typical displacement vector required to align an individual frame was less than one pixel-spacing. However, frames that required more than 3.7 μm correction (*ie.* approaching a cell body diameter, ~ 5 μm) were replaced with the mean image for the epoch and epochs were discarded if they included more than one such frame. Larvae occasionally perform strong motor behaviors ('struggles') and these criteria eliminated such epochs from our analysis. Motion-corrected images were used for all subsequent analysis.

Neurons in GCaMP larvae appear as bright rings of cytoplasm enclosing darker central nuclei ('doughnuts'). Images were segmented to define ROIs corresponding to individual neurons using a watershed-based algorithm [S1]. A high-contrast image was obtained by taking the mean of the (motion-corrected) image time-series. This 'anatomical' image was gaussian-smoothed followed by local histogram stretching and finally a watershed algorithm was used to identify putative somata. ROIs were removed if they had very small (< 5 μm^2) or large (> 100 μm^2) areas. ROIs were manually discarded if they were located in regions of auto-fluorescent skin or outside the brain. Remaining ROIs, corresponding to somata or neuropil regions, were then manually assigned to anatomical structures. By inspecting a random sample of automatically segmented ROIs, we found that error rates (*eg.* merging or splitting of individual somata) were low (Figure S1). Furthermore, we estimated contamination of the fluorescence signals of ROIs by adjacent cells/neuropil as follows: For single voxels, we computed the correlation of that voxel's complete fluorescence time-series with that of the parent ROI versus the highest correlation with any directly adjacent ROI (neighboring cells/neuropil). The percentage of voxels that were better correlated with an adjacent ROI was less than 1% (0.38%, $n = 368,585$ pixels). Inspection of maps of these correlation coefficients suggested this small number of errors most frequently arose when a cell had been erroneously subdivided into more than one ROI.

In our experiments fish were free viewing. To estimate spatial receptive fields (RFs) we corrected for changes in eye position by assessing the difference in the mean eye position mea-

sured during each imaging frame from the median eye position recorded over the course of the experiment. The angular deviation was used to correct the angular position of the moving spot for that frame, allowing us to estimate activity in eye-fixed coordinates. This procedure assumes that eye movements are not associated with spatiotemporal dynamics in RF structure.

The time-varying fluorescence signal $F(t)$ for each cell was extracted by computing the mean value of all pixels comprising an ROI at each time-point (imaging frame). The proportional change in fluorescence ($\Delta F/F$) at time t was calculated as

$$\Delta F/F = \frac{F(t) - F_0}{F_0}$$

where F_0 is a reference fluorescence value, taken as the mean of $F(t)$ during 10 frames prior to visual stimulus presentation. To compute visual response vectors, the mean $\Delta F/F$ time-series was computed across the set of repetitions for each visual stimulus. Mean profiles were then smoothed with a 3-frame box-car filter and the first 20 frames (corresponding to baseline period with no stimulus presentation) were removed. Average responses were then concatenated to produce the visual response vector (VRV) for the cell. VRVs comprised 684 time-points, representing the average response of the ROI to the 18 stimuli in succession.

As a preliminary means to classify ROIs as visually responsive or not, we used t-tests to compare the value of the VRV during stimulus presentation versus during baseline (10 frames prior to stimulus onset). Note that this analysis was performed using VRVs from which we did not remove the baseline frames. For each ROI we performed two t-tests for each of the 18 visual stimuli. The first compared VRV values during baseline versus during stimulus presentation and the second compared baseline to a 2.75 s period immediately following stimulus presentation (as some cells responded to the disappearance of the stimulus). ROIs were classified as visually responsive if any of the 36 tests produced a significant result, at a p-value threshold of 0.02.

We clustered cells from 14 fish based on their VRVs using a method similar to [S6]. The Pearson's correlation between all pairs of VRVs was computed. The two cells with the highest correlation coefficient were then joined into a cluster and the process was repeated by progressively joining cells and clusters until no pairwise correlation exceeded threshold. The centroid of a cluster was taken as the mean of the VRVs within the cluster. We discarded clusters if they did not contain at least 5 cells from 5 different fish as we considered this to be a reasonable standard for identifying reproducible visual response properties. In this procedure, correlation threshold is a free parameter. We found that the number of clusters identified as a function of correlation threshold followed an approximately bell-shaped curve, where few clusters were identified at low threshold (large, poorly tuned clusters) or high threshold (few cells with sufficiently similar VRVs) (Figure S2B). However, there was a range ($\sim$ 0.6–0.8) where a similar number of clusters were

identified, suggesting a consistent partitioning of the data. We selected a correlation threshold of 0.75, which fell within this range.

In Figure S3 we tested an alternative clustering procedure, based on Gaussian Mixture Modeling. First, for each visual stimulus, the peak response of each cell was aligned such that all cells appear to respond at the same time. Principal components analysis was applied to the dataset and the data were projected onto the first 18 principal components. Next, cells were clustered by fitting a Gaussian Mixture Model with 100 components and requiring that for a cell to be assigned to a cluster, the posterior probability must exceed 99%. We discarded clusters containing less than 25 cells, from 5 different fish. Very similar clusters were identified even if we varied the number of components in the model, but clusters became poorly tuned if we relaxed the posterior probability requirement and/or the minimum cell content requirement.

Selectivity Indices for each of the four stimulus features were calculated as:

$$SI_{feature} = \frac{R_1 - R_0}{R_1 + R_0}$$

where R_1 is the sum of the maximum responses (maximum $\Delta F/F$ during spot presentation) for stimuli with feature coded 1 and R_0 is the sum of the maximum responses to the moving spot stimuli with feature coded 0 (refer to binary coding scheme in Figure 2E). Thus, a cell that responds only to leftwards moving spots (leftwards motion is coded 0) will have $SI_{dir} = -1$.

To identify mixed-selectivity neurons, we first computed a vector for each cell describing the peak mean response to each of the 16 moving spot stimuli. We then evaluated the Pearson's correlation coefficient, r, between each neuron's vector and six 16-dimensional binary 'regressors'. The values of the regressors are indicated in Figure 5A. Cells were associated with the regressor that produced the highest r and where $r > 0.75$. Generalized linear regression was used to model the responses of each cell that was associated with a regressor as a function of the four binary stimulus features. The maximum responses to all the individual presentations of the 16 moving spot stimuli were computed (*ie.* 80–128 responses/cell), and this response vector (y) was used to fit the following two models.

Non-linear:

$$y = \beta_0 + \beta_1[Sz \cdot Pol] + \beta_2[Dir \cdot Sz \cdot Pol] + \beta_3[Sp \cdot Sz \cdot Pol] + \beta_4[Dir \cdot Sp \cdot Sz \cdot Pol]$$

Linear:

$$y = \beta_0 + \beta_1[Dir] + \beta_2[Sz] + \beta_3[Sp] + \beta_4[Pol]$$

As a principled means to compare which model provided a more accurate description of the cell's activity we computed a cross-validated R^2 using the 'leave-one-out' method. For each

iteration, a single data point was excluded and the remaining 'training dataset' was used to fit the model parameters. The resulting model was then used to predict the missing datapoint. This process was repeated to predict the full set of responses, which were then compared to the data by computing the proportion of variance explained by the model predictions (cross-validated R^2). We compared the cross-validated R^2 obtained with the linear and non-linear models by computing:

$$SI_{nlin} = \frac{R^2_{nlin} - R^2_{lin}}{R^2_{nlin} + R^2_{lin}}$$

where positive values would indicate the non-linear model provided a more accurate description of the data.

To detect neurons that showed activity modulation associated with hunting responses, we compared activity between response trials and non-response trials during two convergence-triggered 'windows'. The *pre-conv* window was from -3 frames to -1 frames relative to the 'convergence frame' (frame 0, during which the convergent saccade occurred). The *peri-conv* window was from -2 frames to +2 frames relative to the convergence frame. For each cell, we used t-tests to compare $\Delta F/F$ during these windows in response versus non-response trials. We also estimated the maximum signal-to-noise ratio (SNR) during each window in response trials, defined as:

$$SNR = \frac{F(t) - F_0}{\sigma}$$

where σ is the standard deviation of F during the 10 frames prior to stimulus presentation in all epochs corresponding to that visual stimulus. For each window type (pre-conv and peri-conv) we considered response-modulated cells as those with $p < 0.05$ and $SNR > 3$. Note that we applied the SNR criterion to improve the detection of response-modulated cells only after we had determined that we did not detect cells showing negative modulation. To detect tectal assemblies, we fit an ellipse to the spatial distribution of response-modulated cells. The ellipse was centered at the center-of-mass of the cell population with long and short axes equal to the eigenvectors of the covariance matrix of the x, y positions of all modulated cells. Cells were considered part of the assembly if their centroid fell inside this ellipse. Note that this conservative method will often underestimate the number of cells within an assembly. We defined assemblies as having a minimum of 6 cells, with a minimum density of 533 μm^2/cell (ratio of area of ellipse to number of cells) and where at least 70% of cells were located in the same tectal hemisphere. These criteria were established empirically by inspection of maps of response-modulated cells and prevented the detection of assemblies comprising widely scattered cells that were clearly distinct from the coherent clusters of active neurons exemplified in Figure 6. Only ROIs within the tectal SPV were included in our analysis. We estimated onset of assembly activity by finding the first time-point where the mean calcium signal ($\Delta F/F$) of cells within the assembly (population

response) exceeded a threshold (Figure 6P). Threshold was determined by first defining baseline frames as those outside of a 9.35 s window centered on the saccade. The mean and standard deviation of the population response during baseline was computed and threshold was set at mean+4·sd. To estimate false discovery rate, we constructed artificial response and non-response epochs by circularly permuting the activity time-courses of all neurons in a z-plane after removing periods corresponding to convergent saccades (-3 to +3 frames with respect to convergence frame) and visual stimulus presentations. The same permutation was applied to all cells to preserve correlations between neurons as well as the higher order statistics of a single cell's activity. We then evaluated the number and characteristics of assemblies using identical criteria to those applied to the original data. We report false discovery rate as the ratio of the mean number of assemblies detected from 5 permutations to the number detected in the original data.

For all statistical tests, two-tailed p-values are reported.

Supplemental References

S1. Panier, T., Romano, S. A., Olive, R., Pietri, T., Sumbre, G., Candelier, R., and Debrégeas, G. (2013). Fast functional imaging of multiple brain regions in intact zebrafish larvae using selective plane illumination microscopy. Front Neural Circuits *7*, 65.

S2. Bianco, I. H., Kampff, A. R., and Engert, F. (2011). Prey capture behavior evoked by simple visual stimuli in larval zebrafish. Front Syst Neurosci *5*, 101.

S3. Brainard, D. H. (1997). The Psychophysics Toolbox. Spat Vis *10*, 433–6.

S4. Zipfel, W. R., Williams, R. M., and Webb, W. W. (2003). Nonlinear magic: multiphoton microscopy in the biosciences. Nat Biotechnol *21*, 1369–77.

S5. Nestares, O. and Heeger, D. J. (2000). Robust multiresolution alignment of MRI brain volumes. Magn Reson Med *43*, 705–15.

S6. Niell, C. M. and Smith, S. J. (2005). Functional imaging reveals rapid development of visual response properties in the zebrafish tectum. Neuron *45*, 941–51.
